# Plastome Evolution and Phylogeny of Orchidaceae, With 24 New Sequences

**DOI:** 10.3389/fpls.2020.00022

**Published:** 2020-02-21

**Authors:** Young-Kee Kim, Sangjin Jo, Se-Hwan Cheon, Min-Jung Joo, Ja-Ram Hong, Myounghai Kwak, Ki-Joong Kim

**Affiliations:** ^1^ Division of Life Sciences, Korea University, Seoul, South Korea; ^2^ Department of Plant Resources, National Institute of Biological Resources, Incheon, South Korea

**Keywords:** Orchidaceae, plastome evolution, gene loss, IR contraction/expansion, genome rearrangement

## Abstract

In order to understand the evolution of the orchid plastome, we annotated and compared 124 complete plastomes of Orchidaceae representing all the major lineages in their structures, gene contents, gene rearrangements, and IR contractions/expansions. Forty-two of these plastomes were generated from the corresponding author's laboratory, and 24 plastomes—including nine genera (*Amitostigma*, *Bulbophyllum*, *Dactylorhiza*, *Dipodium, Galearis*, *Gymnadenia*, *Hetaeria*, *Oreorchis*, and *Sedirea*)—are new in this study. All orchid plastomes, except *Aphyllorchis montana, Epipogium aphyllum,* and *Gastrodia elata,* have a quadripartite structure consisting of a large single copy (LSC), two inverted repeats (IRs), and a small single copy (SSC) region. The IR region was completely lost in the *A. montana and G. elata* plastomes. The SSC is lost in the *E. aphyllum* plastome. The smallest plastome size was 19,047 bp, in *E. roseum,* and the largest plastome size was 178,131 bp, in *Cypripedium formosanum*. The small plastome sizes are primarily the result of gene losses associated with mycoheterotrophic habitats, while the large plastome sizes are due to the expansion of noncoding regions. The minimal number of common genes among orchid plastomes to maintain minimal plastome activity was 15, including the three subunits of *rpl* (14, 16, and 36), seven subunits of *rps* (2, 3, 4, 7, 8, 11, and 14), three subunits of *rrn* (5, 16, and 23), *trn*C-GCA, and *clp*P genes. Three stages of gene loss were observed among the orchid plastomes. The first was *ndh* gene loss, which is widespread in Apostasioideae, Vanilloideae, Cypripedioideae, and Epidendroideae, but rare in the Orchidoideae. The second stage was the loss of photosynthetic genes (*atp, pet*, *psa,* and *psb*) and *rpo* gene subunits, which are restricted to *Aphyllorchis, Hetaeria, Hexalectris,* and some species of *Corallorhiza* and *Neottia*. The third stage was gene loss related to prokaryotic gene expression (*rpl*, *rps*, *trn,* and others), which was observed in *Epipogium*, *Gastrodia*, *Lecanorchis,* and *Rhizanthella.* In addition, an intermediate stage between the second and third stage was observed in *Cyrtosia* (Vanilloideae). The majority of intron losses are associated with the loss of their corresponding genes. In some orchid taxa, however, introns have been lost in *rpl*16*, rps*16, and *clp*P(2) without their corresponding gene being lost. A total of 104 gene rearrangements were counted when comparing 116 orchid plastomes. Among them, many were concentrated near the IRa/b-SSC junction area. The plastome phylogeny of 124 orchid species confirmed the relationship of {Apostasioideae [Vanilloideae (Cypripedioideae (Orchidoideae, Epidendroideae))]} at the subfamily level and the phylogenetic relationships of 17 tribes were also established. Molecular clock analysis based on the whole plastome sequences suggested that Orchidaceae diverged from its sister family 99.2 mya, and the estimated divergence times of five subfamilies are as follows: Apostasioideae (79.91 mya), Vanilloideae (69.84 mya), Cypripedioideae (64.97 mya), Orchidoideae (59.16 mya), and Epidendroideae (59.16 mya). We also released the first nuclear ribosomal (nr) DNA unit (18S-ITS1-5.8S-ITS2-28S-NTS-ETS) sequences for the 42 species of Orchidaceae. Finally, the phylogenetic tree based on the nrDNA unit sequences is compared to the tree based on the 42 identical plastome sequences, and the differences between the two datasets are discussed in this paper.

## Introduction

Orchidaceae is one of the most flourishing flowering plants and contains about 736 known genera and 28,000 species worldwide (Christenhusz and Byng, 2016). Recent studies recognize five subfamilies within Orchidaceae (Apostasioideae, Vanilloideae, Cypripedioideae, Orchidoideae, and Epidendroideae) as a monophyletic group (Chase et al., 2015). The most recently differentiated subfamily, Epidendroideae, includes about 505 genera and 20,600 species, and accounts for most of Orchidaceae (Chase et al., 2015). Orchidaceae is widely distributed throughout the world, and most members in temperate regions have terrestrial life forms, but orchids in tropical rainforests are known to have mainly epiphyte life forms (Givnish et al., 2015). Non-photosynthetic mycoheterotrophic orchids are found in a total of 43 genera and belong to three subfamilies: Vanilloideae, Orchidoideae, and Epidendroideae (Merckx et al., 2013).

Complete Orchidaceae plastomes have been reported in 38 genera and 118 species (NCBI GenBank, June 30, 2019). There are only five genera (*Corallorhiza*, 10 spp.; *Cymbidium,* 9 spp.; *Dendrobium,* 40 spp.; *Holcoglossum*, 11 spp.; and *Neottia*, 7 spp.) in which at least five plastomes per genus have been decoded (Logacheva et al., 2011; Barrett and Davis, 2012; Yang et al., 2013; Barrett et al., 2014; Feng et al., 2016; Niu et al., 2017b; Barrett et al., 2018; Kim et al., 2018). Among them, *Corallorhiza* and *Neottia* have been subjected to extensive evolutionary studies of their plastomes because both photosynthetic and non-photosynthetic species occur in a congeneric group (Barrett and Davis, 2012; Barrett et al., 2014; Feng et al., 2016; Barrett et al., 2018). In addition to the two genera, evolutionary studies have been carried out on the plastomes of some species of other orchid genera. For examples, extensive gene losses have been reported in several independent mycoheterotrophic orchid lineages—i.e., *Aphyllorchis* (Feng et al., 2016), *Cyrtosia* (Kim et al., 2019), *Epipogium* (Schelkunov et al., 2015), *Gastrodia* (Yuan et al., 2018), *Hexalectris* (Barrett and Kennedy, 2018) and *Rhizanthella* (Delannoy et al., 2011). In addition, *ndh* deletion and pseudogenization are assumed to be phenomena that occur independently in many orchid lineages such as *Apostasia* (Lin et al., 2017; Niu et al., 2017a), *Calypso* (Barrett et al., 2018), *Cattleya* (da Rocha Perini et al., 2016), *Cephalanthera* (Feng et al., 2016), *Cremastra* (Dong et al., 2018), *Cymbidium* (Yang et al., 2013; Kim et al., 2018; Wang et al., 2018), *Dendrobium* (Niu et al., 2017b), *Epipactis* (Dong et al., 2018), *Eulophia* (Huo et al., 2017), *Holcoglossum* (Li et al., 2019), *Limodorum* (Lallemand et al., 2019), *Liparis* (Krawczyk et al., 2018), *Neuwiedia* (Niu et al., 2017a), *Oncidium* (Wu et al., 2010; Kim et al., 2015a), *Paphiopedilum* (Niu et al., 2017b; Hou et al., 2018), *Phalaenopsis* (Chang et al., 2006), *Phragmipedium* (Kim et al., 2015a), *Platanthera* (Dong et al., 2018), *Vanilla* (Lin et al., 2015), and *Vanda* (Li et al., 2019). On the other hand, full *ndh* genes have been reported in members of *Anoectochilus* (Yu et al., 2016), *Calanthe* (Dong et al., 2018), *Cypripedium* (Kim et al., 2015b; Lin et al., 2015), *Habenaria* (Lin et al., 2015; Kim et al., 2017b), *Masdevallia* (Kim et al., 2015a), *Ophrys* (Roma et al., 2018), *Pleione* (Shi et al., 2018), and *Sobralia* (Kim et al., 2015a). Gene relocations within a plastome often occur in the reduced plastome of orchids such as *Cyrtosia* (Kim et al., 2019), *Hexalectris* (Barrett and Kennedy, 2018), and *Rhizanthella* (Delannoy et al., 2011). Similar gene rearrangement events within a plastome have been reported in Campanulaceae (Cosner et al., 1997), conifers (Hirao et al., 2008), Fabaceae (Cai et al., 2008), Geraniaceae (Chumley et al., 2006), and Oleaceae (Lee et al., 2007). Contractions of the small single copy (SSC) region similar to that of Geraniaceae were also reported in *Paphiopedilum* (Kim et al., 2015a; Niu et al., 2017b; Hou et al., 2018) and *Vanilla* (Lin et al., 2015; Amiryousefi et al., 2017).

Phylogenetic studies of Orchidaceae using entire plastomes are in a relatively early stage because of limited available plastome sequences (Givnish et al., 2015; Kim et al., 2015a; Lin et al., 2015; Niu et al., 2017b; Dong et al., 2018). But the relationships among major orchid lineages determined using whole plastomes agree well to the large-scale phylogenetic studies of Orchidaceae using two or three genes (Górniak et al., 2010; Freudenstein and Chase, 2015). Therefore, several outstanding phylogenetic problems in Orchidaceae will be resolved if more plastome sequences are accumulated.

In this study we first completely decoded the plastomes of 24 taxonomic groups of Orchidaceae, including nine genera (*Amitostigma*, *Bulbophyllum*, *Dactylorhiza*, *Dipodium*, *Galearis*, *Gymnadenia*, *Hetaeria*, *Oreorchis*, and *Sedirea*) for which the plastomes had not yet been decoded previously. Second, this study re-annotated and compared the entire plastome sequences of 129 taxa comprising 124 Orchidaceae and five outgroups to investigate evolutionary directions in orchid plastomes, such as sizes, gene contents, gene losses, gene rearrangements, and inverted repeat (IR) expansions/contractions. Third, three stages of gene loss patterns were inferred from mycoheterotrophic orchids and the minimum genes required for plastid maintenance were inferred. Fourth, the phylogenetic trees of Orchidaceae were constructed using whole plastome sequences, and the times at which each taxonomic group differentiated were inferred. Among the 124 orchid plastomes completely decoded, 42 were produced by the NGS method in the laboratory of the corresponding author, and the nuclear ribosomal RNA gene unit (18S-ITS1-5.8S-ITS2-28S-NTS-ETS) was also annotated at the same time. Finally, the common features and differences among the 42 species were compared and explained by comparing their plastome trees and nrDNA gene unit trees.

## Materials and Methods

### Plant Materials and DNA Extraction

Plant leaf materials used in this study and their voucher information are given in **Table 1**. Fresh leaf samples were ground into fine powder with liquid nitrogen in a mortar. Ground samples were used to extract genomic DNA using a G-spin™ II Genomic DNA Extraction Kit (Intron, Seoul, Korea). The quality of DNA was checked by a UVVIS spectrophotometer. Extracted DNAs were deposited into the Plant DNA Bank in Korea (PDBK) and voucher specimens were deposited into the Korea University (KUS) herbarium and National Institute of Biological Resources (NIBR) herbarium.

**Table 1:** NGS data status and general features of the 24 newly sequenced Orchidaceae plastomes.

Subfamily	Tribe	Scientific Name	NGS Method	# of raw reads	# of trimmed reads	Coverages (X)	Length (bp)	Voucher specimen and/or DNA number
**Epidendroideae**	Collabieae	*Calanthe aristulifera*	MiSeq	10,977,664	555,650	851.4	158,204	NIBRVP0000709265
**Epidendroideae**	Collabieae	*Calanthe bicolor*	HiSeq	38,596,340	585,710	1,252.6	158,070	PDBK2012-1749
**Epidendroideae**	Cymbidieae	*Dipodium roseum*	MiSeq	23,515,448	871,246	1,733.7	141,209	PDBKTA2019-0006
**Epidendroideae**	Epidendreae	*Corallorhiza maculata* var. *maculata*	MiSeq	10,817,394	424,348	841.7	146,198	PDBK2017-1544
**Epidendroideae**	Epidendreae	*Cremastra unguiculata*	MiSeq	10,280,912	438,352	884.1	159,341	NIBRVP0000658615
**Epidendroideae**	Epidendreae	*Oreorchis patens*	MiSeq	8,530,614	553,290	363.8	158,542	PDBK2007-0151
**Epidendroideae**	Gastrodieae	*Gastrodia elata*	MiSeq	12,318,196	676,842	546.2	35,056	PDBK2017-1545
**Epidendroideae**	Malaxideae	*Bulbophyllum inconspicuum*	MiSeq	9,612,620	604,200	1,125.2	149,548	PDBK2012-0213
**Epidendroideae**	Malaxideae	*Dendrobium moniliforme*	HiSeq	8,745,236	614,130	869.0	151,711	PDBK2012-0008
**Epidendroideae**	Malaxideae	*Dendrobium moniliforme* ‘Royal Dream'	HiSeq	20,728,340	616,358	1,211.9	151,695	PDBK2014-0012
**Epidendroideae**	Malaxideae	*Dendrobium moniliforme* ‘Sangeum'	HiSeq	10,876,884	673,118	1277.4	151,711	PDBK2014-0009
**Epidendroideae**	Malaxideae	*Liparis auriculata*	MiSeq	8,360,832	686,628	1,353.2	153,460	NIBRVP0000703422
**Epidendroideae**	Malaxideae	*Liparis makinoana*	MiSeq	14,299,168	705,140	1,384.2	153,093	PDBK2012-0543
**Epidendroideae**	Neottieae	*Epipactis thunbergii*	MiSeq	21,093,888	897,388	706.4	159,279	PDBK2017-0509
**Epidendroideae**	Vandeae	*Sedirea japonica*	MiSeq	12,019,968	391,094	842.2	146,942	PDBK2019-0551
**Orchidoideae**	Cranichideae	*Goodyera rosulacea*	MiSeq	18,736,600	137,286	287.4	152,831	PDBK2012-0647
**Orchidoideae**	Cranichideae	*Hetaeria shikokiana*	MiSeq	10,227,090	105,074	540.2	130,934	PDBK2018-0672
**Orchidoideae**	Orchideae	*Amitostigma gracile*	MiSeq	8,563,894	137,432	220.4	156,120	PDBK2008-0404
**Orchidoideae**	Orchideae	*Dactylorhiza viridis* var. *coreana*	MiSeq	8,179,110	180,240	266.6	153,549	PDBK2011-0840
**Orchidoideae**	Orchideae	*Galearis cyclochila*	MiSeq	7,859,188	224,736	397.2	153,928	PDBK2000-0786
**Orchidoideae**	Orchideae	*Gymnadenia conopsea*	MiSeq	8,967,924	220,376	550.3	153,876	PDBK2011-0894
**Orchidoideae**	Orchideae	*Habenaria chejuensis*	MiSeq	8,865,896	359,614	576.0	153,896	PDBK2018-1246
**Orchidoideae**	Orchideae	*Habenaria flagellifera*	MiSeq	10,712,312	233,302	439.0	151,210	PDBK2018-1247
**Orchidoideae**	Orchideae	*Platanthera mandarinorum*	MiSeq	8,745,236	221,761	541.1	154,162	PDBK2013-0398

### NGS Sequencing, Plastome Assembly, and Annotation

Four samples—*Calanthe bicolor*, *Dendrobium moniliforme*, *D*. *moniliforme* “Royal Dream,” and *D*. *moniliforme* “Sangeum”—were sequenced by Illumina HiSeq 2000. Twenty other samples were sequenced by Illumina MiSeq. The resulting raw reads from HiSeq were trimmed by Geneious 6.1.8 (Kearse et al., 2012) with a 0.05 error probability limitation. *Dendrobium officinale* (NC024019) was used as a reference sequence to perform reference-guided assembly using a Geneious assembler. Contigs from the reference-guided assembly were used as reference sequences to perform reference-guided assembly repeatedly until complete plastome sequences were obtained.

The resulting raw reads from MiSeq were trimmed by BBDuk 37.64, implemented in Geneious 11.1.5 (length: 27 kmer). BBNorm 37.64 was used to normalize trimmed reads (target coverage level: 30; minimum depth: 12). Normalized reads were used to perform *de-novo* assembly by Geneious assembler. Resulting *de-novo* assembly contigs were used as reference sequences to perform reference-guided assembly to obtain complete plastome sequences when the *de-novo* assembly did not produce complete plastome contigs. All complete plastome sequences were used as reference sequences to gather chloroplast reads from trimmed read sets. Gathered reads were reused to perform *de-novo* assembly and validate the level of completeness. Due to its unusual plastome structure, *Gastrodia elata* was assembled again by SPAdes 3.10.0 (Bankevich et al., 2012) using an error correction tool and assembly module. NGS results are given in **Table 1**.

Complete plastome sequences were annotated with BLASTn, tRNAscan-SE 2.0 (Lowe and Chan, 2016), ORF finder, and find annotation function in Geneious 11.1.5 (*Habenaria radiata* plastome sequences, NC035834, used as a reference). Alternative start codons—such as ACG and TTG—were also included in the ORF finder. Genes with many stop codons in the middle of sequences, uncorrectable frame shift mutations, and several large abnormal indels, were judged to have a pseudogene. Gene sequences with complete CDS or few (five or fewer) internal stop codons and for which RNA editing was possible were analyzed to have a gene. These criteria were based on a character reconstructions study of the gene status of *ndh* in Orchidaceae (Kim et al., 2015a). ORFs from the plastome sequence of four mixotrophic orchids (*Corallorhiza maculata* var. *maculata*, *Dipodium roseum*, *Gastrodia elata,* and *Hetaeria shikokiana*) were translated into protein sequences. The translated sequences were exported in the fasta format files to perform psi-blast (Altschul et al., 1997) based on several Orchidaceae plastome databases. Circular plastome maps were constructed using the OGdraw web server (Lohse et al., 2007). All downloaded NCBI plastome data used in our study were re-annotated because the published data contained many annotation errors. Finally, we manually edited some ambiguous annotation area using our comparative alignments of orchid sequences.

### Phylogenetic Analysis

Ninety-nine plastome sequences were downloaded from the NCBI to perform phylogenetic analysis (**Table 2**; 94 Orchidaceae sequences, four Asparagales sequences, and one Liliales sequences). Eight to 79 CDS and two to four rRNA genes were extracted from plastome sequences. Each extracted region was aligned by MAFFT (Katoh et al., 2002) and all alignments were checked manually. Each aligned gene sequences were subjected to jModeltest (Darriba et al., 2012) in CIPRES Science Gateway (Miller et al., 2010) to obtain the best model. GTR-I-G-X or GTR-G-X were the best-fit models for all genes except *rrn*5 (short sequence), for which SYM-G was the best-fit model. Eighty-three alignments were concatenated to a length of 87,399 bp. Concatenated alignments were used to perform jModeltest (Darriba et al., 2012) in CIPRES Science Gateway (Miller et al., 2010) to obtain the best model. GTR+G+I was the best-fit model for concatenated data. Lost genes were treated as missing data because they do not affect the phylogenetic signals in the remaining genes (Lam et al., 2016). A maximum likelihood (ML) tree was constructed using RaxML-HPC2 on XSEDE in CIPRES Science Gateway with a GTR+G+I model and 100 bootstrap replicates (−672774.637133) of ML optimization likelihood. Concatenated alignment was also used to construct a Bayesian inference (BI) tree. MrBayes_CIPRES api (Miller et al., 2015) was used to construct a BI tree with a Markov chain Monte Carlo (MCMC) chain length of 1,000,000 and GTR+G+I model. The trees obtained were treated graphically using Treegraph2 (Stöver and Müller, 2010).

**Table 2:** Information on the sequences used in this study.

Subfamily	Tribe	Scientific name*	NCBI accession (nrDNA)	NCBI accession (Plastome)	Life style	Length (bp)	LSC (bp)	SSC (bp)	IR (bp)	GC %	# of coding genes	# of pseudo- genes	# of tRNAs
**Epidendroideae**	Arethuseae	*Bletilla ochracea*	*–*	NC029483	Terrestrial	157,431	85,810	17,949	26,836	37.3	74	9	30
**Epidendroideae**	Arethuseae	*Bletilla striata*	*–*	NC028422	Terrestrial	157,393	86,213	17,742	26,719	37.2	74	9	29
**Epidendroideae**	Arethuseae	*Pleione bulbocodioides*	*–*	NC036342	Terrestrial	159,269	87,121	18,712	26,718	37.2	83	0	30
**Epidendroideae**	Collabieae	*Calanthe aristulifera*	**MN221390**	**MN200378**	Terrestrial	158,204	86,889	18,369	26,473	36.7	83	0	30
**Epidendroideae**	Collabieae	*Calanthe bicolor*	**MN221391**	**MN200379**	Terrestrial	158,070	87,000	18,342	26,364	36.7	83	0	30
**Epidendroideae**	Collabieae	*Calanthe davidii*	*–*	NC037438	Terrestrial	153,629	86,045	15,672	25,956	36.9	83	0	30
**Epidendroideae**	Collabieae	*Calanthe triplicata*	*–*	NC024544	Terrestrial	158,759	87,305	18,460	26,497	36.7	83	0	30
**Epidendroideae**	Cymbidieae	*Cymbidium goeringii*	*–*	NC028524	Terrestrial	157,192	85,749	17,883	26,780	36.9	74	9	30
**Epidendroideae**	Cymbidieae	*Cymbidium lancifolium*	*–*	NC029712	Terrestrial	149,945	84,716	13,895	25,667	37.1	73	8	30
**Epidendroideae**	Cymbidieae	*Cymbidium macrorhizon*	MK333261	KY354040	Terrestrial	149,859	85,187	13,766	25,453	37.0	73	8	30
**Epidendroideae**	Cymbidieae	*Dipodium roseum*	**MN221399**	**MN200386**	Terrestrial	141,209	81,787	10,352	24,535	36.9	71	3	30
**Epidendroideae**	Cymbidieae	*Erycina pusilla*	*–*	NC018114	Epiphyte	143,164	83,733	11,675	23,878	36.7	71	6	29
**Epidendroideae**	Cymbidieae	*Eulophia zollingeri*	*–*	NC037212	Terrestrial	145,201	81,566	13,091	25,272	36.9	73	6	29
**Epidendroideae**	Cymbidieae	*Oncidium sphacelatum*	*–*	NC028148	Epiphyte	147,761	83,575	12,670	25,758	37.1	73	4	30
**Epidendroideae**	Epidendreae	*Calypso bulbosa* var. *occidentalis*	*–*	NC040980	Terrestrial	149,313	83,331	14,718	25,632	37.1	72	8	29
**Epidendroideae**	Epidendreae	*Cattleya crispata*	*–*	NC026568	Epiphyte	148,343	85,973	13,261	24,495	37.3	75	7	30
**Epidendroideae**	Epidendreae	*Cattleya liliputana*	*–*	NC032083	Epiphyte	147,092	85,945	13,149	24,304	37.4	75	7	30
**Epidendroideae**	Epidendreae	*Corallorhiza bulbosa*	*–*	NC025659	Terrestrial	148,643	82,851	12,368	26,712	37.1	72	7	29
**Epidendroideae**	Epidendreae	*Corallorhiza macrantha*	*–*	NC025660	Terrestrial	151,031	84,263	12,544	27,112	37.2	70	9	29
**Epidendroideae**	Epidendreae	*Corallorhiza maculata* var. *maculata* 1	**MN221392**	**MN200380**	Terrestrial	146,198	79,612	13,018	26,784	36.8	53	22	30
**Epidendroideae**	Epidendreae	*Corallorhiza maculata* var. *maculata* 2	*–*	KM390014	Terrestrial	146,886	80,395	12,885	26,803	36.8	55	25	29
**Epidendroideae**	Epidendreae	*Corallorhiza maculata* var. *mexicana*	*–*	KM390015	Terrestrial	151,506	84,347	12,671	27,244	37.1	71	8	29
**Epidendroideae**	Epidendreae	*Corallorhiza maculata* var. *occidentalis*	*–*	KM390016	Terrestrial	146,595	81,363	12,368	26,432	36.9	58	22	30
**Epidendroideae**	Epidendreae	*Corallorhiza mertensiana*	*–*	NC025661	Terrestrial	147,941	81,109	13,774	26,529	36.8	58	22	29
**Epidendroideae**	Epidendreae	*Corallorhiza odontorhiza*	*–*	NC025664	Terrestrial	147,317	82,257	13,508	25,776	37.0	71	8	29
**Epidendroideae**	Epidendreae	*Corallorhiza striata* var. *vreelandii*	*–*	JX087681	Terrestrial	137,505	72,631	12,388	26,243	36.4	46	30	29
**Epidendroideae**	Epidendreae	*Corallorhiza trifida*	*–*	NC025662	Terrestrial	149,384	83,092	14,420	25,936	37.2	72	9	29
**Epidendroideae**	Epidendreae	*Corallorhiza wisteriana*	*–*	NC025663	Terrestrial	146,437	76,350	17,743	26,172	37.1	71	8	29
**Epidendroideae**	Epidendreae	*Cremastra appendiculata*	*–*	NC037439	Terrestrial	155,320	87,098	15,478	26,372	37.2	76	5	30
**Epidendroideae**	Epidendreae	*Cremastra unguiculata*	**MN221393**	**MN200381**	Terrestrial	159,341	87,031	18,252	27,029	36.9	82	0	30
**Epidendroideae**	Epidendreae	*Hexalectris warnockii*	*–*	MH444822	Terrestrial	119,057	66,903	17,490	17,332	36.9	41	26	29
**Epidendroideae**	Epidendreae	*Masdevallia coccinea*	*–*	NC026541	Terrestrial	157,423	84,957	18,448	27,009	36.8	83	0	30
**Epidendroideae**	Epidendreae	*Masdevallia picturata*	*–*	NC026777	Epiphyte	156,045	84,948	18,029	26,534	36.9	83	0	29
**Epidendroideae**	Epidendreae	*Oreorchis patens*	**MN221415**	**MN200369**	Terrestrial	158,542	86,437	18,443	26,831	36.9	83	0	30
**Epidendroideae**	Gastrodieae	*Gastrodia elata* 2	*–*	NC037409	Terrestrial	35,304	(SC Only)	(SC Only)	-	34.2	23	2	5
**Epidendroideae**	Gastrodieae	*Gastrodia elata* 1	**MN221402**	**MN200389**	Terrestrial	35,056	(SC Only)	(SC Only)	-	26.7	23	2	5
**Epidendroideae**	Malaxideae	*Bulbophyllum inconspicuum*	**MN221389**	**MN200377**	Epiphyte	149,548	85,760	12,136	25,826	37.0	78	0	30
**Epidendroideae**	Malaxideae	*Dendrobium moniliforme* 2	*–*	NC035154	Epiphyte	148,778	84,867	11,945	25,983	37.5	70	5	30
**Epidendroideae**	Malaxideae	*Dendrobium moniliforme* 1	**MN221396**	**MN200384**	Epiphyte	151,711	84,773	14,352	26,293	37.5	73	7	30
**Epidendroideae**	Malaxideae	*Dendrobium moniliforme* ‘Royal Dream'	**MN221397**	**MN200385**	Epiphyte	151,695	84,758	14,351	26,293	37.5	73	7	30
**Epidendroideae**	Malaxideae	*Dendrobium moniliforme* ‘Sangeum'	**MN221398**	**MN200383**	Epiphyte	151,711	84,773	14,352	26,293	37.5	73	7	30
**Epidendroideae**	Malaxideae	*Dendrobium nobile*	*–*	NC029456	Epiphyte	153,660	85,686	14,654	26,660	37.5	73	7	30
**Epidendroideae**	Malaxideae	*Dendrobium officinale*	*–*	NC024019	Epiphyte	152,221	85,109	14,516	26,298	37.5	73	7	30
**Epidendroideae**	Malaxideae	*Liparis auriculata*	**MN221412**	**MN200365**	Terrestrial	153,460	83,785	17,731	25,972	36.9	83	0	30
**Epidendroideae**	Malaxideae	*Liparis loeselii*	*-*	MF374688	Terrestrial	153,687	84,596	17,673	25,709	36.9	74	9	29
**Epidendroideae**	Malaxideae	*Liparis makinoana*	**MN221413**	**MN200368**	Terrestrial	153,093	83,533	17,746	25,907	36.9	83	0	30
**Epidendroideae**	Malaxideae	*Oberonia japonica*	MN221414	NC035832	Epiphyte	142,996	81,669	10,969	25,179	37.4	72	2	30
**Epidendroideae**	Neottieae	*Aphyllorchis montana*	*–*	NC030703	Terrestrial	94,559	(SC Only)	(SC Only)	-	37.1	38	26	30
**Epidendroideae**	Neottieae	*Cephalanthera humilis*	*–*	NC030706	Terrestrial	157,011	86,908	15,133	27,485	37.3	71	10	30
**Epidendroideae**	Neottieae	*Cephalanthera longifolia*	*–*	NC030704	Terrestrial	161,877	88,806	19,187	26,942	37.2	83	0	30
**Epidendroideae**	Neottieae	*Epipactis mairei*	*–*	NC030705	Terrestrial	159,019	86,377	18,816	26,913	37.3	81	2	30
**Epidendroideae**	Neottieae	*Epipactis thunbergii*	**MN221400**	**MN200387**	Terrestrial	159,279	87,297	18,638	26,672	37.3	83	0	30
**Epidendroideae**	Neottieae	*Epipactis veratrifolia*	*–*	NC030708	Terrestrial	159,719	87,043	18,854	26,911	37.3	82	1	30
**Epidendroideae**	Neottieae	*Limodorum abortivum*	–	MH590355	Terrestrial	154,847	85,544	15,099	27,102	37.5	76	0	30
**Epidendroideae**	Neottieae	*Neottia acuminata*	*–*	NC030709	Terrestrial	83,190	51,145	5,371	13,337	36.6	31	20	30
**Epidendroideae**	Neottieae	*Neottia camtschatea*	*–*	NC030707	Terrestrial	106,385	52,960	9,273	22,076	37.2	38	29	30
**Epidendroideae**	Neottieae	*Neottia fugongensis*	*–*	NC030711	Terrestrial	156,536	85,357	18,311	26,434	31.6	83	0	30
**Epidendroideae**	Neottieae	*Neottia listeroides*	*–*	NC030713	Terrestrial	110,246	45,021	9,597	27,814	37.2	31	25	30
**Epidendroideae**	Neottieae	*Neottia nidus-avis*	*–*	NC016471	Terrestrial	92,060	36,422	7,822	23,908	34.4	29	8	27
**Epidendroideae**	Neottieae	*Neottia ovata*	*–*	NC030712	Terrestrial	156,978	85,433	18,071	26,737	37.6	83	0	30
**Epidendroideae**	Neottieae	*Neottia pinetorum*	*–*	NC030710	Terrestrial	155,959	84,449	18,104	26,703	37.5	82	1	30
**Epidendroideae**	Neottieae	*Palmorchis pabstii*	*–*	NC041190	Terrestrial	163,909	90,710	18,823	27,188	37.3	83	0	30
**Epidendroideae**	Nervilieae	*Epipogium aphyllum*	*–*	NC026449	Terrestrial	30,650	8,030	0	11,310	32.8	21	0	6
**Epidendroideae**	Nervilieae	*Epipogium roseum*	*–*	NC026448	Terrestrial	19,047	9,618	8,907	261	30.6	22	0	7
**Epidendroideae**	Sobralieae	*Elleanthus sodiroi*	*–*	NC027266	Terrestrial	161,511	88,425	18,880	27,103	37.1	83	0	30
**Epidendroideae**	Sobralieae	*Sobralia* aff. *bouchei* HTK-2015	*–*	NC028209	Terrestrial/Epiphyte	161,543	88,684	18,835	27,012	37.1	83	0	30
**Epidendroideae**	Sobralieae	*Sobralia callosa*	*–*	NC028147	Terrestrial	161,430	88,666	18,794	26,985	37.1	83	0	30
**Epidendroideae**	Vandeae	*Gastrochilus fuscopunctatus*	MK317970	NC035830	Epiphyte	146,183	83,125	11,146	25,956	36.8	72	2	30
**Epidendroideae**	Vandeae	*Gastrochilus japonicus*	MK317969	NC035833	Epiphyte	147,697	84,695	11,174	25,914	36.8	72	2	30
**Epidendroideae**	Vandeae	*Holcoglossum amesianum*	*–*	NC041511	Epiphyte	148,074	84,250	12,026	25,899	36.6	72	4	30
**Epidendroideae**	Vandeae	*Holcoglossum flavescens*	*–*	NC041512	Epiphyte	146,863	83,288	11,959	25,808	36.7	72	4	30
**Epidendroideae**	Vandeae	*Holcoglossum lingulatum*	*–*	NC041465	Epiphyte	146,525	83,762	11,274	25,769	36.8	72	6	30
**Epidendroideae**	Vandeae	*Neofinetia falcata*	MK317968	KT726909	Epiphyte	146,491	83,802	11,775	25,457	36.6	72	4	30
**Epidendroideae**	Vandeae	*Neofinetia falcata* ‘CheongSan'	MK317971	NC036372	Epiphyte	146,497	83,808	11,775	25,457	36.6	72	4	30
**Epidendroideae**	Vandeae	*Neofinetia richardsiana*	MK317967	NC036373	Epiphyte	146,498	83,809	11,775	25,457	36.6	72	4	30
**Epidendroideae**	Vandeae	*Pelatantheria scolopendrifolia*	MK317972	NC035829	Epiphyte	146,860	86,075	11,735	24,525	36.5	72	1	30
**Epidendroideae**	Vandeae	*Pendulorchis himalaica*	*–*	NC041513	Epiphyte	145,207	83,712	11,413	25,041	36.6	72	4	30
**Epidendroideae**	Vandeae	*Phalaenopsis aphrodite* subsp. *formosana*	*–*	NC007499	Epiphyte	148,964	85,957	11,543	25,732	36.7	71	3	30
**Epidendroideae**	Vandeae	*Phalaenopsis equestris*	*–*	NC017609	Epiphyte	148,959	85,967	11,300	25,846	36.7	71	8	30
**Epidendroideae**	Vandeae	*Phalaenopsis* ‘Tiny Star'	*–*	NC025593	Epiphyte	148,918	85,885	11,523	25,755	36.7	71	7	31
**Epidendroideae**	Vandeae	*Sedirea japonica*	**MN221419**	**MN200373**	Epiphyte	146,942	84,882	10,568	25,746	32.2	72	2	30
**Epidendroideae**	Vandeae	*Thrixspermum japonicum*	MK317966	NC035831	Epiphyte	149,220	85,301	11,546	26,187	36.1	71	1	30
**Epidendroideae**	Vandeae	*Vanda brunnea*	*–*	NC041522	Epiphyte	149,216	85,783	11,713	25,860	36.7	72	7	30
**Orchidoideae**	Cranichideae	*Anoectochilus emeiensis*	*–*	NC033895	Terrestrial	152,650	82,670	17,342	26,319	36.9	80	0	31
**Orchidoideae**	Cranichideae	*Goodyera fumata*	*–*	NC026773	Terrestrial	155,643	84,077	18,342	26,612	37.3	83	0	30
**Orchidoideae**	Cranichideae	*Goodyera procera*	*–*	NC029363	Terrestrial	153,240	82,032	18,406	26,401	37.6	76	0	31
**Orchidoideae**	Cranichideae	*Goodyera rosulacea*	**MN221403**	**MN200390**	Terrestrial	152,831	82,041	17,720	26,535	36.8	83	0	30
**Orchidoideae**	Cranichideae	*Goodyera schlechtendaliana*	*–*	NC029364	Terrestrial	154,348	83,215	18,051	26,541	37.2	79	0	31
**Orchidoideae**	Cranichideae	*Goodyera velutina*	*–*	NC029365	Terrestrial	152,692	82,443	17,247	26,501	36.9	76	0	31
**Orchidoideae**	Cranichideae	*Hetaeria shikokiana*	**MN221408**	**MN200367**	Terrestrial	130,934	64,694	2,320	31,960	36.3	44	19	30
**Orchidoideae**	Cranichideae	*Kuhlhasseltia nakaiana*	MN221409	KY354041	Terrestrial	147,614	81,617	13,673	26,162	39.5	73	0	30
**Orchidoideae**	Cranichideae	*Ludisia discolor*	*–*	NC030540	Terrestrial	153,054	82,675	17,233	26,573	37.0	82	0	30
**Orchidoideae**	Diurideae	*Rhizanthella gardneri*	*–*	NC014874	Terrestrial	59,190	26,360	13,295	9,767	34.2	24	4	9
**Orchidoideae**	Orchideae	*Amitostigma gracile*	**MN221388**	**MN200376**	Terrestrial	156,120	85,171	18,165	26,392	36.4	83	0	30
**Orchidoideae**	Orchideae	*Dactylorhiza viridis* var. *coreana*	**MN221395**	**MN200382**	Terrestrial	153,549	82,715	17,728	26,553	31.9	83	0	30
**Orchidoideae**	Orchideae	*Galearis cyclochila*	**MN221401**	**MN200388**	Terrestrial	153,928	83,308	17,772	26,424	36.9	83	0	30
**Orchidoideae**	Orchideae	*Gymnadenia conopsea*	**MN221404**	**MN200391**	Terrestrial	153,876	83,142	17,702	26,516	38.6	83	0	29
**Orchidoideae**	Orchideae	*Habenaria chejuensis*	**MN221405**	**MN200392**	Terrestrial	153,896	83,732	17,026	26,569	36.6	82	0	30
**Orchidoideae**	Orchideae	*Habenaria flagellifera*	**MN221406**	**MN200366**	Terrestrial	151,210	81,072	17,110	26,514	36.7	83	0	30
**Orchidoideae**	Orchideae	*Habenaria pantlingiana*	*–*	NC026775	Terrestrial	153,951	83,641	17,370	26,470	36.6	83	0	30
**Orchidoideae**	Orchideae	*Habenaria radiata*	MN221407	NC035834	Terrestrial	155,353	84,833	17,718	26,401	36.5	83	0	30
**Orchidoideae**	Orchideae	*Ophrys fusca* subsp. *iricolor*	*–*	AP018716	Terrestrial	150,177	80,541	16,940	26,348	36.8	83	0	22
**Orchidoideae**	Orchideae	*Ophrys sphegodes*	*–*	AP018717	Terrestrial	146,754	80,471	16,177	25,053	36.9	83	0	22
**Orchidoideae**	Orchideae	*Platanthera japonica*	*–*	NC037440	Terrestrial	154,995	85,979	13,664	27,676	37.0	82	0	30
**Orchidoideae**	Orchideae	*Platanthera mandarinorum*	**MN221416**	**MN200370**	Terrestrial	154,162	83,325	17,757	26,540	36.8	83	0	30
**Cypripedioideae**	Cypripedioideae	*Cypripedium formosanum*	*–*	NC026772	Terrestrial	178,131	102,188	21,921	27,011	33.9	83	0	31
**Cypripedioideae**	Cypripedioideae	*Cypripedium japonicum*	*–*	NC027227	Terrestrial	174,417	97,322	21,911	27,592	34.5	82	1	30
**Cypripedioideae**	Cypripedioideae	*Paphiopedilum armeniacum*	*–*	NC026779	Terrestrial	162,682	91,734	3,666	33,641	35.4	72	5	30
**Cypripedioideae**	Cypripedioideae	*Paphiopedilum dianthum*	*–*	NC036958	Epiphyte	154,699	86,861	2,416	32,711	35.9	71	3	30
**Cypripedioideae**	Cypripedioideae	*Paphiopedilum niveum*	*–*	NC026776	Terrestrial	159,108	89,856	5,194	32,029	35.7	71	5	30
**Cypripedioideae**	Cypripedioideae	*Phragmipedium longifolium*	*–*	NC028149	Terrestrial	151,157	88,367	13,066	24,862	36.1	71	4	30
**Vanilloideae**	Pogonieae	*Pogonia japonica*	MN221417	MN200371	Terrestrial	158,200	87,447	5,387	32,683	36.4	72	4	30
**Vanilloideae**	Pogonieae	*Pogonia minor*	MN221418	MN200372	Terrestrial	158,170	87,457	5,375	32,669	36.4	72	4	30
**Vanilloideae**	Vanilleae	*Cyrtosia septentrionalis*	MN221394	MH615835	Terrestrial	96,859	58,085	10,414	17,946	34.8	41	0	25
**Vanilloideae**	Vanilleae	*Lecanorchis japonica*	MN221410	MN200364	Terrestrial	70,498	28,197	14,493	13,904	30.4	25	3	7
**Vanilloideae**	Vanilleae	*Lecanorchis kiusiana*	MN221411	MN200363	Terrestrial	74,084	30,824	14,118	14,571	30.0	25	2	8
**Vanilloideae**	Vanilleae	*Vanilla aphylla*	*–*	NC035320	Epiphyte	150,184	87,379	2,131	30,337	35.0	65	7	29
**Vanilloideae**	Vanilleae	*Vanilla madagascariensis*	MN221420	MN200374	Epiphyte	151,552	87,490	1,254	31,404	34.6	71	1	29
**Vanilloideae**	Vanilleae	*Vanilla planifolia* 2	*–*	NC026778	Epiphyte	148,011	86,358	2,037	29,808	35.4	72	1	30
**Vanilloideae**	Vanilleae	*Vanilla planifolia* 1	MN221421	MN200375	Epiphyte	147,714	86,061	2,037	29,808	35.4	72	1	30
**Vanilloideae**	Vanilleae	*Vanilla pompona*	*–*	NC036809	Epiphyte	148,009	86,358	2,037	29,807	35.4	72	1	30
**Apostasioideae**	Apostasioideae	*Apostasia odorata*	*–*	NC030722	Terrestrial	159,285	86,172	18,765	27,174	35.7	82	1	30
**Apostasioideae**	Apostasioideae	*Apostasia wallichii*	*–*	NC036260	Terrestrial	156,126	83,031	20,187	26,454	36.1	73	10	30
**Apostasioideae**	Apostasioideae	*Neuwiedia zollingeri* var. *singapureana*	*–*	LC199503	Terrestrial	161,068	88,910	18,056	27,051	36.0	73	10	29
**Amarylidaceae (Outgroup)**	Amarylidaceae (Outgroup)	*Allium cepa*	*–*	KM088013	Terrestrial	153,529	82,662	17,931	26,468	36.8	82	0	30
**Asparagaceae (Outgroup)**	Asparagaceae (Outgroup)	*Eustrephus latifolius*	*–*	NC025305	Terrestrial	159,736	82,403	13,607	31,863	38.1	78	2	30
**Iridaceae (Outgroup)**	Iridaceae (Outgroup)	*Iris gatesii*	*–*	NC024936	Terrestrial	153,441	82,702	18,371	26,184	37.9	83	0	29
**Iridaceae (Outgroup)**	Iridaceae (Outgroup)	*Iris sanguinea*	*–*	NC029227	Terrestrial	152,408	82,340	18,016	26,026	38.0	83	0	30
**Liliales (Outgroup)**	Liliales (Outgroup)	*Fritillaria hupehensis*	*–*	NC024736	Terrestrial	152,145	81,894	17,553	26,349	37.0	80	2	30

*Red scientific name indicates that the species is mixotroph or obligate (full) mycoheterotroph throughout its life cycle. The bold NCBI accession numbers for nrDNAs and plastomes indicate that the species’ NGS data were generated in this study.

IRScope (Amiryousefi et al., 2018) was used to describe SC-IR junction regions among 41 plastome sequences generated in the laboratory (*Gastrodia elata* was excluded due to its lack of IR). The length information of 24 new plastome sequences was visualized by ggplot2 package in R (Wickham et al., 2016). Gene contents of 129 plastome sequences were displayed as a heatmap. The ProgressiveMauve algorithm (Darling et al., 2010) was used to check plastome rearrangements among 116 Orchidaceae plastome sequences, excluding non-photosynthetic orchids with an extremely short plastome (*Cyrtosia*, *Epipogium*, *Gastrodia*, *Lecanorchis,* and *Rhizanthella*). The resulting LCBs (locally collinear blocks) from ProgressiveMauve were extracted and numbered to visualize their features (**Tables S1** and **S2**).

The nuclear rDNA region—which contains 18S rRNA, internal transcribed spacers, 5.8S rRNA, 28S rRNA, NTS, and ETS—was generated in 42 Orchidaceae species (**Table 2**). Forty-two rDNA sequences were aligned by MAFFT (6,043 bp long). The alignment was tested with jModeltest in CIPRES Science Gateway. RaxML-HPC2 on XSEDE in CIPRES Science Gateway was used to construct an ML tree with a GTR+G+I model and 100 bootstrap replicates. The plastome sequence gene data of 42 species were also used to construct an ML tree. Eighty-three CDS and rRNA genes were extracted and aligned by MAFFT. Eighty-three alignments were concatenated into one (80,798 bp long). Concatenated alignments were used to perform jModeltest. An ML tree was constructed by RaxML-HPC2 on XSEDE with a GTR+G+I model with 100 bootstrap replicates. The trees obtained were treated by Treegraph2.

### Divergence Time Estimation

The alignments used in the ML tree construction were also used to estimate divergence time. The GTR estimated model was selected to build a time divergence tree, following the results of PartifionFinder v2.1.1 (Lanfear et al., 2012). An XML file was prepared by BEAUti 2.5.2 (Bouckaert et al., 2019). The XML file was submitted to the CIPRES Science Gateway to perform BEAST2-XSEDE (Bouckaert et al., 2019). A relaxed clock log normal model (Drummond et al., 2006) and Yule model were chosen to perform MCMC with a chain length of 300,000,000. Logs and trees were collected every 5,000 generations, and three independent runs were performed. Three fossil data (Asparagales, normal distribution, sigma 8.0, mean 105.3; *Dendrobium*, log-normal distribution, sigma 2.0, offset 23.2; and *Goodyera*, log-normal distribution, sigma 2.0, offset 15.0) were used to calibrate nodes (Ramírez et al., 2007; Conran et al., 2009; Gustafsson et al., 2010; Iles et al., 2015).

Three log and tree files were concatenated by Logcombiner v2.5.2 (Rambaut and Drummond, 2014) by discarding 20% of files. The concatenated log files were checked by Tracer v1.6 (Rambaut et al., 2014) to validate the effective sample size (ESS). Major parameters—including posterior, likelihood, and the prior—exceeded an ESS of 100, and all other parameters exceeded an ESS of 50. The concatenated tree files were treated by Treeannotator (Rambaut and Drummond, 2007) in CIPRES Science Gateway with an option of 0.95 posterior probability. The concatenated maximum clade credibility tree generated by Treeannotator was treated by FigTree v1.4 (Rambaut, 2012) and “phytools” and “ape” packages in R.

## Results

### General Features of 24 New Orchidaceae Plastomes

The taxonomic positions, NGS methods, raw read numbers, trimmed read numbers, plastome lengths, coverage depth, voucher information, etc. of the 24 new plastomes are summarized in **Table 1**. The coverage depths ranged from 220x (*Amitostigma gracile*) to 1,734x (*Dactylorhiza viridis* var. *coreana*), so that each plastome sequence was sequenced at least several hundred times.

**Figure 1:**
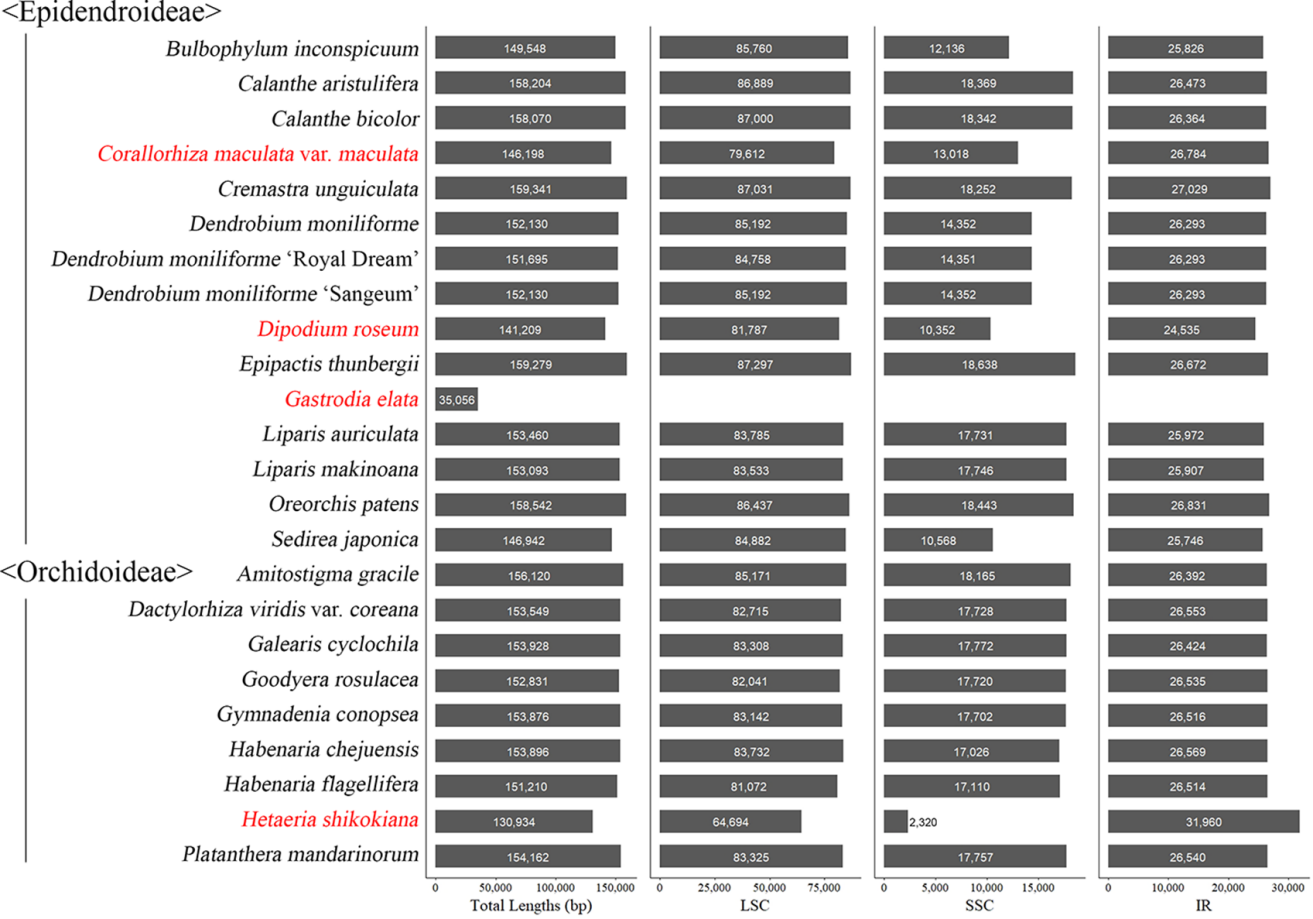
Plastome length variation in 24 newly sequenced orchid species. The plastome of *Gastrodia elata* is 35,056 bp long and consists of only one single copy region. The species names in red indicate mycoheterotrophic species.

Among the plastomes of the 24 newly decoded species, the length of the plastome of *Gastrodia elata*, a non-photosynthetic species, was the shortest at 35,056 bp and that of *Cremastra unguiculata*, a photosynthetic species, was the longest at 159,341 bp. *Gastrodia elata* is unique in that it only has a single copy of the plastome because the IR was lost. The plastomes of the remaining 23 species have quadripartite structures consisting of an large single copy (LSC), an SSC, and two IR regions. The total lengths of the plastomes and the relative lengths of the IR, SSC, and LSC of the 24 newly decoded species were compared, as shown in **Figure 1**.

Among the 24 species, the plastomes of 13 (*Amitostigma gracile*, *Calanthe aristulifera*, *C. bicolor, Dactylorhiza viridis* var. *coreana, Epipactis thunbergii, Galearis cyclochila, Goodyera rosulacea, Habenaria chejuensis, H. flagellifera, Liparis auriculata, L. makinoana, Oreorchis patens,* and *Platanthera mandarinorum*) had all the same genes as typical plant plastids. Various subunits of the *ndh* gene class were found to be pseudogenized or lost in eight species (*Bulbophyllum inconspicuum, Cremastra unguiculata, Dendrobium moniliforme, D. moniliforme* “Sangeum”, *D. moniliforme* “Royal Dream”, *Dipodium roseum, Gymnadenia conopsea*, and *Sedirea japonica*). In addition, *pet*L was lost in *Cremastra unguiculata, psb*D was pseudogenized in *Dipodium roseum*, and *trn*G-UCC was lost in *Gymnadenia conopsea*. In the case of the non-photosynthetic species *Corallorhiza maculata* var. *maculata*, *ccs*A, *cem*A, *ndh*B, *ndh*C, *ndh*D, *ndh*G, *ndh*H, *ndh*I, *ndh*J, *ndh*K, *pet*A, *pet*D, *pet*G, *psa*A, *psa*B, *psb*A, *psb*C, *rbc*L, *rpo*A, *rpo*B, *rpo*C1, and *ycf*1 existed as pseudogenes and *ndh*A, *ndh*E, *ndh*F, *psb*B, *psb*J, *psb*L, *psb*M, and *rpo*C2 were lost. In the case of *Hetaeria shikokiana*, another non-photosynthetic species, many genes were lost or pseudogenized. Among the genes, *cem*A, *ndh*A, *ndh*B, *ndh*C, *ndh*H, *pet*A, *pet*D, *pet*G, *pet*N, *psa*B, *psa*I, *psb*C, *psb*F, *psb*N, *rpo*A, *rpo*C1, *rpo*C2, *ycf*3, and *ycf*4 were pseudogenized, and *atp*A, *atp*B, *ccs*A, *ndh*D, *ndh*E, *ndh*F, *ndh*G, *ndh*I, *ndh*J, *pet*B, *psa*A, *psa*C, *psb*A, *psb*B, *psb*J, *psb*L, *psb*M, *rbc*L, *rpl*23, and *rpo*B were lost. *Gastrodia elata*, which has the shortest plastome of the 24 species examined, only had the following genes: *acc*D, *clp*P, *mat*K, *rpl*2, *rpl*14, *rpl*16, *rpl*20, *rpl*36, *rps*2, *rps*3, *rps*4, *rps*7, *rps*8, *rps*11, *rps*12, *rps*14, *rps*18, *rps*19, *rrn*5, *rrn*16, *rrn*23, *ycf*1, and *ycf*2. Two pseudogenes (*psa*I and *psb*K) remained, and all the other genes were lost.

### Comparative Analyses of Orchidaceae Plastomes

**Figure 2:**
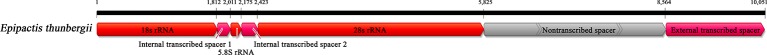
Diagram of nuclear ribosomal (nr) DNA repeat units consisting of 18S-ITS1-5.8S-ITS2-28S-NTS-ETS. The total length of the unit is approximately 10 kb, and it is arranged as a tandem repeat. The nrDNA repeat sequences of 42 orchid species were first reported in this paper.

The Orchidaceae plastomes that have been completely decoded and can be used in comparative studies comprise 60 genera, 142 species, and 146 accessions, including the 24 new plastomes (NCBI database, June 30, 2019). Since the main purpose of this study was to identify the evolutionary trends in plastomes of the entire Orchidaceae, there were three genera—*Cymbidium* (9 spp.), *Dendrobium* (40 spp.), and *Holcoglossum* (11 spp.)—for which the plastomes of many species were decoded, but only three species each were included in the comparative study. In the case of *Corallorhiza* (10 spp.) and *Neottia* (7 spp.), the plastomes of all species were included in the comparative study, even though the plastomes of many species were decoded, because these genera include both photosynthetic and non-photosynthetic species. In the case of the remaining genera, all available plastomes were included in the comparative study except for one species of *Cypripedium,* in which many problems were found in the re-annotation process. Therefore, five subfamilies, 17 tribes, 60 genera, 118 species, and 124 accessions were used in the comparative study in this paper. In addition, five outgroup plastomes were also used for comparison. Therefore, the GenBank accession numbers, taxonomic classification, habitats, plastome sizes, LSC lengths, IR lengths, SSC lengths, GC contents, and numbers of genes and pseudogenes of the 129 plastomes used in this study, along with whether or not their corresponding species is photosynthetic, are listed in **Table 2**. The plastomes of land plants usually have an AT-biased base composition. Furthermore, highly reduced plastomes have more AT-biased substitutions than typical plastomes because they experience relaxed selection. The average GC content of orchid plastomes in this study was 36.40 ± 1.71%. The three highly reduced orchid plastomes—*Epipogium aphyllum*, *E. roseum*, and *Rhizanthella*—had 32.8%, 30.6%, and 34.2% GC contents, respectively. The CDS of *E. aphyllum., E. roseum*, and *Rhizanthella* had 38.4%, 34.3%, and 38.1% GC contents, respectively. They showed 2–6% more AT-biased substitutions than general orchid plastomes. Extreme AT bias was reported in the plastome of *Rhopalocnemis phalloides*, which had a GC content of only 13.2% (Schelkunov et al., 2019). Among the 124 Orchidaceae plastomes used in the comparative study, 42 were decoded using the NGS method in the laboratory of the corresponding author. In addition to the plastomes, the base sequences of nuclear ribosomal RNA repeating unit DNA (nrDNA, 18S-ITS1-5.8S-ITS2-28S-NTS-ETS), which are present as tandem repeats, could also be assembled in these 42 species (**Figure 2**). Therefore, the GenBank accession numbers of these species are also published in **Table 2** for the first time. Consequently, these 42 species were used to compare nrDNA repeating unit-based trees and plastome-based trees.

**Figure 3:**
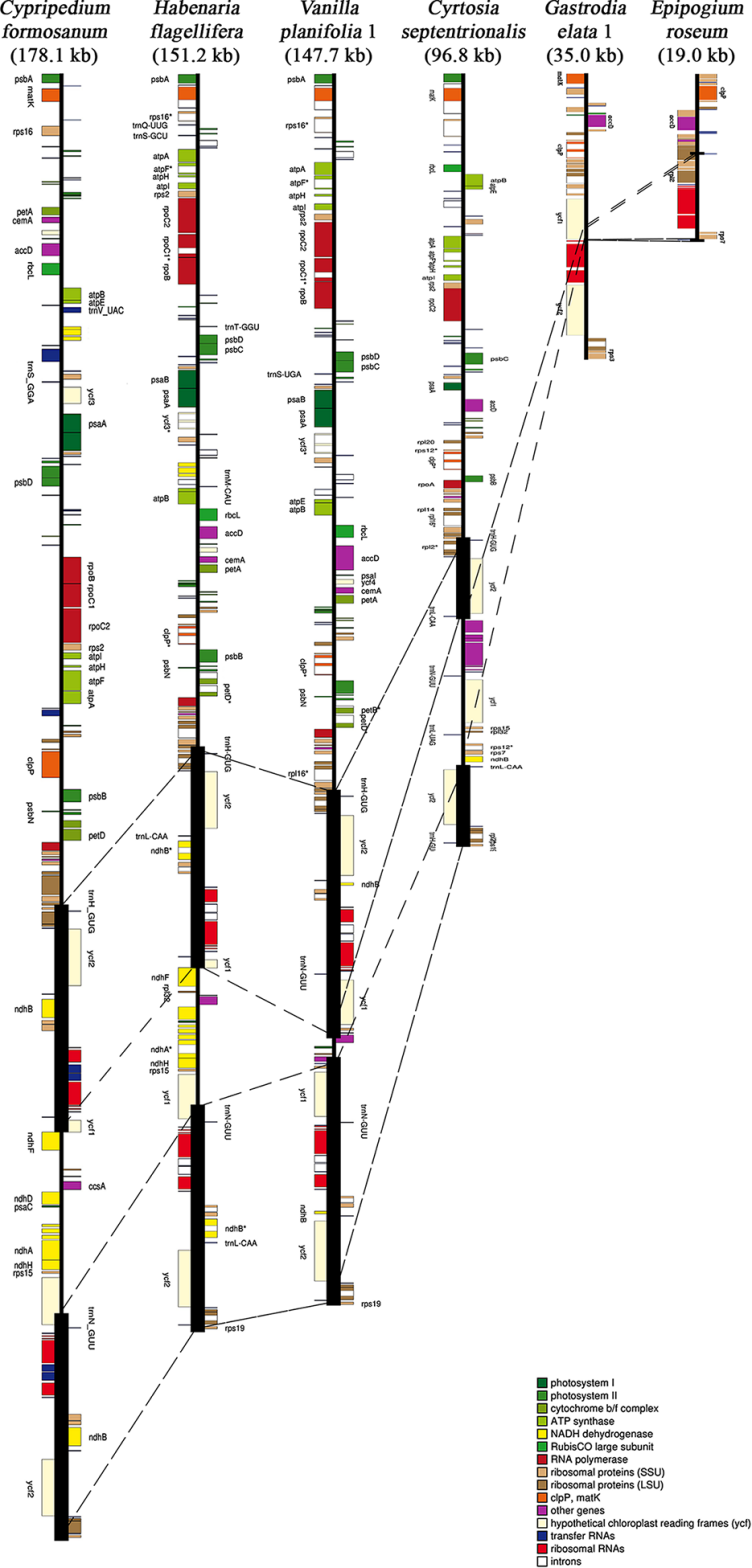
Six representative plastomes of Orchidaceae. *Cypripedium formosanum* has the longest plastome (178.1 kb), while *Epipogium roseum* has the shortest. *Gastrodia elata* and *Epipogium roseum* hold 27 genes in their plastome, even though they have substantially different plastome sizes. The heavy vertical bars indicate inverted repeat (IR) regions and the broken lines among plastomes indicate the boundaries of IR and single copy regions.

Among the 124 Orchidaceae plastomes, 121 had an IR region. In the case of *Aphyllorchis montana*, the IR region was lost and the plastome existed only as a single copy (**Table 2**), similarly to *Gastrodia elata* 1 and 2. The plastome of *Aphyllorchis montana* is small at 94,559 bp. Another example of the SSC region being lost and the plastome consisting of the LSC and two IRs was reported in *Epipogium aphyllum* (**Figure 3**). These three species are examples of the quadripartite structure of a plastome not being maintained. The quadripartite structure was maintained in all the remaining species. *Epipogium aphyllum* is the only example in which the IRs appear consecutively without any SSC. However, in the case of *Epipogium roseum*, which is a related species, an SSC region, although short, existed at 890 bp, and the IR region was shortened to 261 bp (**Table 2**, **Figure 3**).

**Figure 4:**
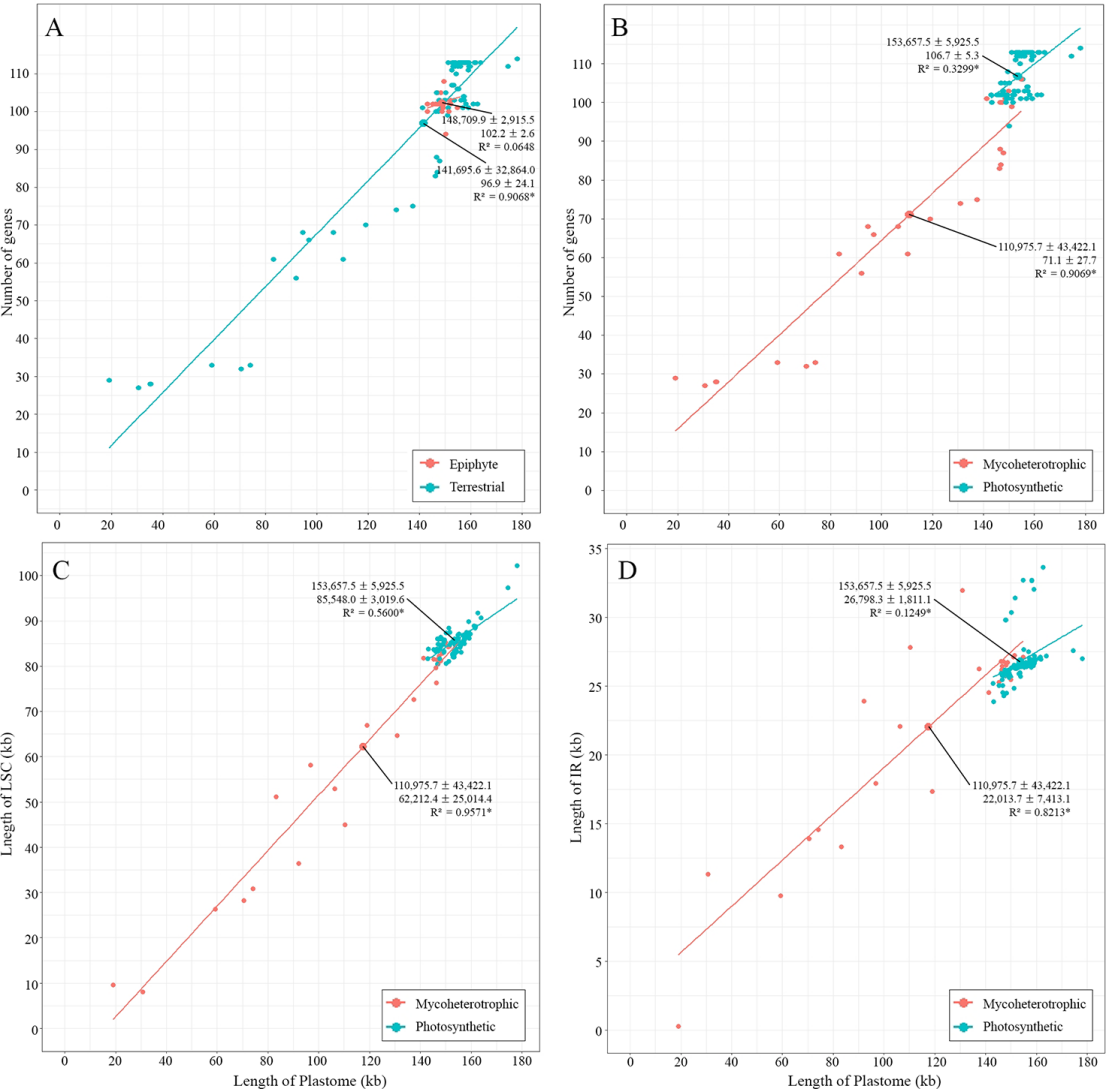
Relationships between plastome lengths and gene numbers. **(A)**: Terrestrial orchids show a wider range of variation than epiphytic orchids. **(B)**: Mycoheterotrophic orchids show a wider range of variation than photosynthetic orchids. **(C**, **D)**: Plastome lengths are more strongly correlated with LSC lengths than IR lengths in both mycoheterotrophic and photosynthetic orchids.

The largest plastome in Orchidaceae was 178,131 bp, in *Cypripedium formosanum,* and the smallest was 19,047 bp, in *Epipogium roseum* (**Figure 3**). The plastome sizes of all photosynthetic orchids were at least 140 kb. On the other hand, the plastome sizes of non-photosynthetic mycoheterotrophic orchids were mostly smaller than 150 kb and showed high positive correlations with gene numbers (**Figure 4A**). In addition, unlike the plastome sizes of epiphytic orchids, which were at least 140 kb, those of terrestrial orchids varied greatly because they include mycoheterotrophic species (**Figure 4B**). Although the plastome sizes of photosynthetic orchids mostly ranged from 145–160 kb, those of *Cypripedium* had a much larger derange; this was highly correlated with the expansion of the LSC region, and not correlated much with the expansion of IRs (**Figures 4C, D**). On the other hand, in the case of mycoheterotrophic species, plastome sizes showed high correlations with the size of both the LSC and IR.

### Gene Content Evolution of Orchidaceae

**Figure 5:**
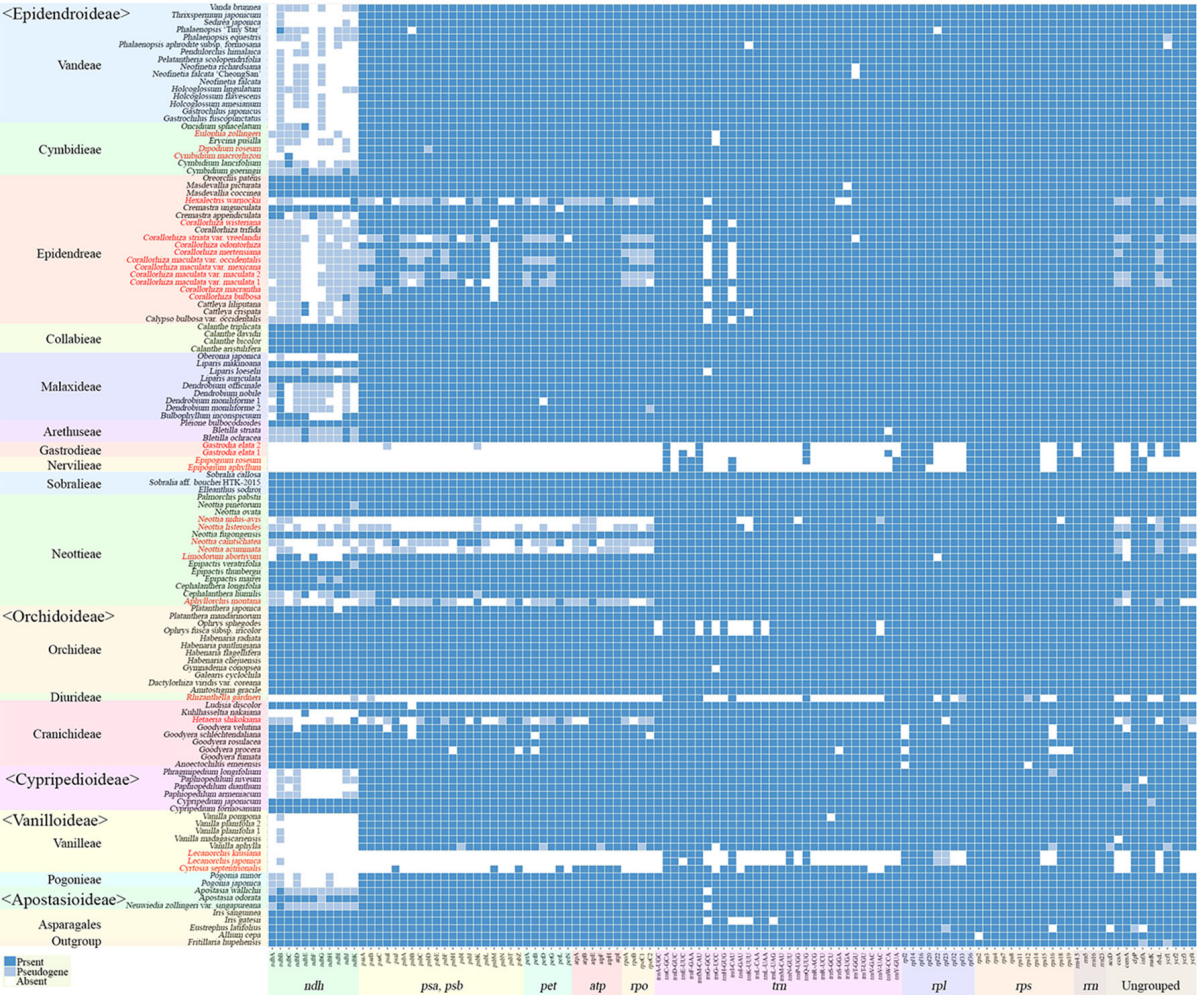
Distribution patterns of gene loss in Orchidaceae. The dark blue, light blue, and white blocks indicate presence, pseudogene, and absence of each gene, respectively. The non-functionalization of 11 *ndh* genes are distributed widely across all taxonomic groups of Orchidaceae. This frequently occurs in Epidendroideae and Vanilloideae. The non-functionalization of *psa*, *psb*, *pet*, and *rpo* gene classes are confined to mycoheterotrophic lineages. In addition, the loss of housekeeping genes such as the *rps*, *rpl*, or *trn* gene classes occur independently in four genera, *Epipogium*, *Gastrodia*, *Lecanorchis,* and *Rhizanthella*. The species names in red indicate mycoheterotrophic orchids.

The gene contents of the 124 orchid species showed high variation (**Figure 5**). A plastome usually contains a total of 113 genes, comprising 6 *atp*, 11 *ndh*, 6 *pet*, 9 *rpl*, 4 *rpo*, 12 *rps*, 4 *rrn*, 5 *psa*, 15 *psb*, 30 *trn*, and 11 ungrouped genes. Among the 124 species of Orchidaceae, 27 (22%) have all 113 genes existing in an active status. In eight species, one to three genes were pseudogenized or lost. These 35 species fall into the category of having plastomes with almost all their plastid genes, and they make up 28% of all 124 species in this study. There are 69 species where four to 11 of the 11 genes in the *ndh* gene class or all 11 *ndh* genes plus one or two other gene(s) do not function, indicating that the plastid *ndh* gene class does not function in 56% of all 124 species. Furthermore, *ndh* genes, photosynthesis light reaction genes (*pet, psa*, *psb*), and *rpo* gene were shown to be lost in 13 species. Such cases are also accompanied by the loss of *rpo* genes. Finally, in the case of *Lecanorchis* (two species), *Epipogium* (two species), *Gastrodia* (one species, two accs.), and *Rhizanthella* (one species), many housekeeping genes such as *rpl*, *rps*, and *trn* were also lost.

The presence of genes and pseudogenization in the 124 Orchidaceae and five outgroup species are set forth in **Figure 5** by subfamily, tribe, species, and gene class. In the case of photosynthetic orchids, up to 13 genes were lost. In most of these cases, 11 *ndh* genes and one or two other genes were lost. In an exceptional case with photosynthetic orchids, 19 genes were lost in *Vanilla aphylla* with epiphytic habitats. The lost genes seem to consist of 11 *ndh* genes and eight other pseudogenized genes. However, given that only 11 to 12 genes were lost from the plastids in other *Vanilla* species, it is inferred that the plastid gene annotation of this species is problematic. On the other hand, among the species known to be mycoheterotrophic species, the fewest genes were lost in *Limodorum abortivum*, where the loss of seven genes comprising *cem*A, five *ndh* genes, and *rpl*22 was observed. *Limodorum abortivum* was followed by *Cymbidium macrorhizon*, in which 10 *ndh* genes were lost, *Eulophia zollingeri*, where 11 *ndh* genes and *trn*G-UCC were lost, and *Dipodium roseum*, where 11 *ndh* genes and a *psb*D gene were lost. However, although these four species have the mycoheterotrophic nutritional mode, they also have chlorophyll, and are reported or observed to be orchids that carry out low levels of photosynthesis. In the case of *Corallorhiza*, both mycoheterotrophic and photosynthetic species exist, and the levels of photosynthesis vary according to variety or habitat, even in the same species. When seen based on the degree of gene loss, species in which 11 *ndh* genes and one to three other genes were lost are very likely to preserve photosynthetic activity. When the gene contents of the 124 orchid plastomes were compared, a large gap was found in the area where 14–25 genes were lost. Therefore, whether photosynthetic ability was lost or not can be divided around this range (**Figures 4** and **5**).

Among the plastomes that are classified into completely non-photosynthetic orchids because at least 25 genes were lost, two groups are recognized based on the numbers of lost genes and preserved genes. That is, they are a group of plastomes in which the number of preserved genes is similar to or larger than the number of lost genes (13 plastomes), and a group of plastomes in which the number of lost genes is overwhelmingly larger (seven plastomes). Among the 124 plastomes, the Orchidaceae species that belong to the latter are *Epipogium aphyllum* (86 lost, 27 preserved), *Epipogium roseum* (84 lost, 29 preserved), *Gastrodia elata* 1 and 2 (85 lost, 28 preserved), *Lecanorchis kiusiana* (80 lost, 33 preserved), *Lecanorchis japonica* (81 lost, 32 preserved), and *Rhizanthella gardneri* (80 lost, 33 preserved). These seven plastomes have 27–33 genes preserved, and among them, 20 genes are common (**Table 3**). These 20 genes are inferred to be the minimum common plastid genes necessary to maintain non-photosynthetic plastids. However, among these 20 plastid genes, five are missing in any of the remaining 117 Orchidaceae plastomes. Therefore, 15 genes are common to all orchid plastids. The 15 genes comprise 14 housekeeping genes (*rpl*14, *rpl*16, *rpl*26, *rps*2, *rps*3, *rps*4, *rps*7, *rps*8, *rps*11, *rps*14, *rrn*5, *rrn*16, *rrn*23, and *trn*C-GGA) and *clp*P.

**Table 3:** Minimum genes required for seven orchid species with extremely degraded plastomes (*Epipogium aphyllum*, *E*. *roseum*, two *Gastrodia elata*, *Lecanorchis japonica*, *L*. *kiusiana,* and *Rhizanthella gardneri*).

Scientific Name Gene	*Epipogium aphyllum*	*Epipogium roseum*	*Gastrodia elata*	*Gastrodia elata*	*Lecanorchis japonica*	*Lecanorchis kiusiana*	*Rhizanthella gardneri*	Other Orchids	Notes
***acc*D**	+	+	+	+	+	+	+	116/117	*Vanilla aphylla* (Pseudogene)
*clp*P*	**+**	**+**	**+**	**+**	**+**	**+**	**+**	117	
***rpl*2**	+	+	+	+	+	+	+	113/117	*Anoectochilus emeiensis* (Absent), *Goodyera procera* (Absent), *G. schlechtendaliana* (Absent), *G. velutina* (Absent)
*rpl*14*	**+**	**+**	**+**	**+**	**+**	**+**	**+**	117	
*rpl*16*	**+**	**+**	**+**	**+**	**+**	**+**	**+**	117	
*rpl*36*	**+**	**+**	**+**	**+**	**+**	**+**	**+**	117	
*rps*2*	**+**	**+**	**+**	**+**	**+**	**+**	**+**	117	
*rps*3*	**+**	**+**	**+**	**+**	**+**	**+**	**+**	117	
*rps*4*	**+**	**+**	**+**	**+**	**+**	**+**	**+**	117	
*rps*7*	**+**	**+**	**+**	**+**	**+**	**+**	**+**	117	
*rps*8*	**+**	**+**	**+**	**+**	**+**	**+**	**+**	117	
*rps*11*	**+**	**+**	**+**	**+**	**+**	**+**	**+**	117	
*rps*14*	**+**	**+**	**+**	**+**	**+**	**+**	**+**	117	
***rps*18**	+	+	+	+	+	+	+	115/117	*Neottia nidus-avis* (Absent), *Goodyera procera* (Absent)
***rps*19**	+	+	+	+	+	+	+	116/117	*Goodyera procera* (Absent)
*rrn*5*	**+**	**+**	**+**	**+**	**+**	**+**	**+**	117	
*rrn*16*	**+**	**+**	**+**	**+**	**+**	**+**	**+**	117	
*rrn*23*	**+**	**+**	**+**	**+**	**+**	**+**	**+**	117	
*trn*C-GCA*	**+**	**+**	**+**	**+**	**+**	**+**	**+**	117	
***trn*fM-CAU**	**+**	**+**	**+**	**+**	**+**	**+**	**+**	115/117	*Ophrys fusca* subsp. *iricolor* (Absent) *Ophrys sphegodes* (Absent)

Bold gene name with an asterisk indicates that all 124 orchid species share the gene.

**Figure 6:**
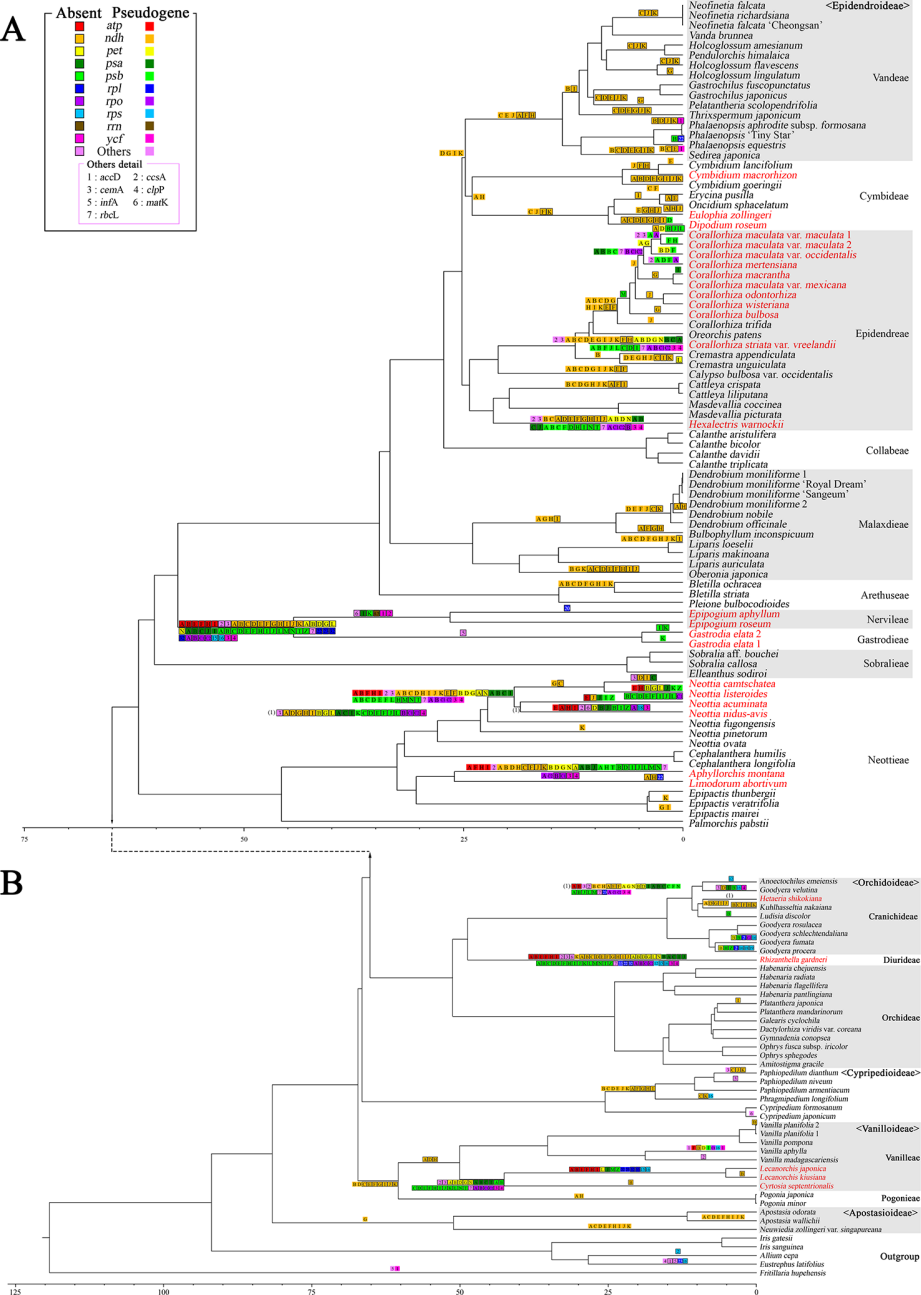
Evolution of gene losses in the phylogenetic tree of Orchidaceae. A total of 129 taxa—124 Orchidaceae and five outgroup taxa—were the subject of tree reconstruction. The sequences of 83 protein coding genes were concatenated to a length of 87,399 bp. A maximum likelihood (ML) tree was constructed using RaxML-HPC2 with a GTR+G+I model (ML = −672774.637133 of ML optimization likelihood). All the genes were then plotted on the tree node using parsimony criteria under the condition of no parallel gains of the same gene. The species names in red indicate mycoheterotrophic orchids. **(A)**: The basal portion of the tree showing the subfamilies Apostasioideae, Vanilloideae, Cypripedioideae, and Orchidoideae. **(B)**: The upper portions of the tree showing the subfamily Epidendroideae.

To determine the patterns of plastome gene loss over Orchidaceae evolution, we plotted the gene loss patterns on the ML phylogenetic trees using Bayesian estimation approaches (**Figure 6**). Apostasioideae, Vanilloideae, Cypripedioideae, and Orchidoideae are shown in **Figure 6A**, and Epidendroideae is shown in **Figure 6B**. In the case of the *ndh* gene class, gene loss or pseudogenization occurred independently in almost all lineages of Epidendroideae. On the other hand, in the case of the four remaining subfamilies, the loss of *ndh* genes, although relatively rare, was observed independently in at least five lineages. The loss of genes directly involved in the photosynthetic light reaction—such as *psa*, *psb*, and *pet*—was observed in six lineages of Epidendroideae, two lineages of Orchidoideae, and one lineage of Vanilloideae. The loss of housekeeping genes such as *rpl*, *rps*, and *trn* occurred independently in three lineages: *Gastrodia*-*Epipogium* of Epidendroideae, *Lecanorchis* of Vanilloideae, and *Rhizanthella* of Orchidoideae.

**Figure 7:**
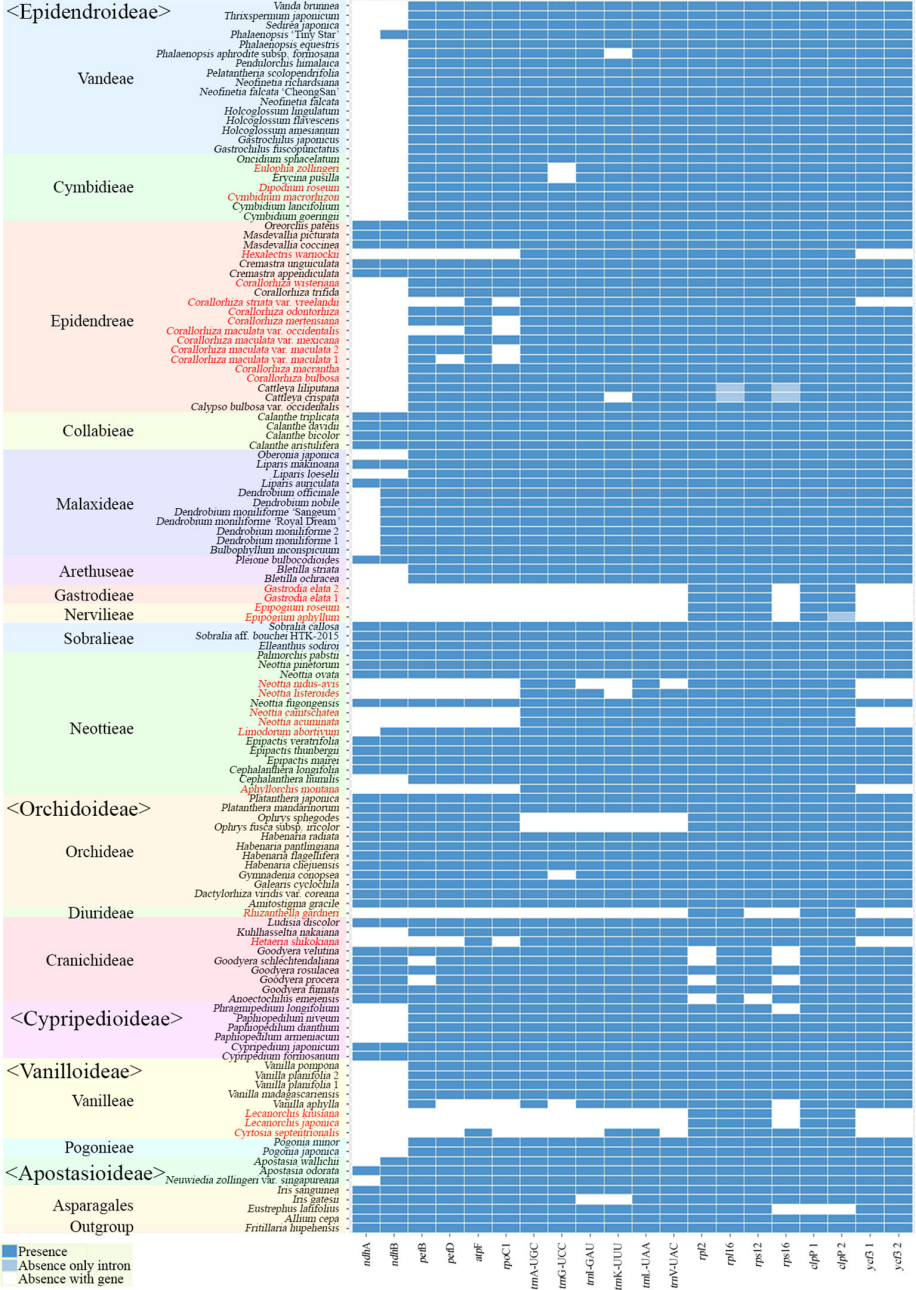
Distribution patterns of the loss of 20 introns in Orchidaceae. The dark blue, light blue, and white blocks indicate intron presence, absence without corresponding gene loss, and absence with corresponding gene loss, respectively.

Sixteen genes in the typical land plant plastomes (*atp*F, *ndh*A, *ndh*B, *pet*B, *pet*D, *rpl*2, *rpl*16, *rpo*C1, *rps*12, *rps*16, *trn*A-UGC, *trn*G-UCC, *trn*I-GAU, *trn*K-UUU, *trn*L-UAA, and *trn*V-UAC) generally have one intron, and two genes (*clpP* and *ycf3*) have two introns. The absence or presence of these 20 introns are summarized (**Figure 7**). The majority of intron losses are associated with the loss of their corresponding gene; the exceptions are *rpl*16*, rps*16, and *clp*P(2), in which the introns were lost but the corresponding genes were not in some terminal clade of the tree.

**Figure 8:**
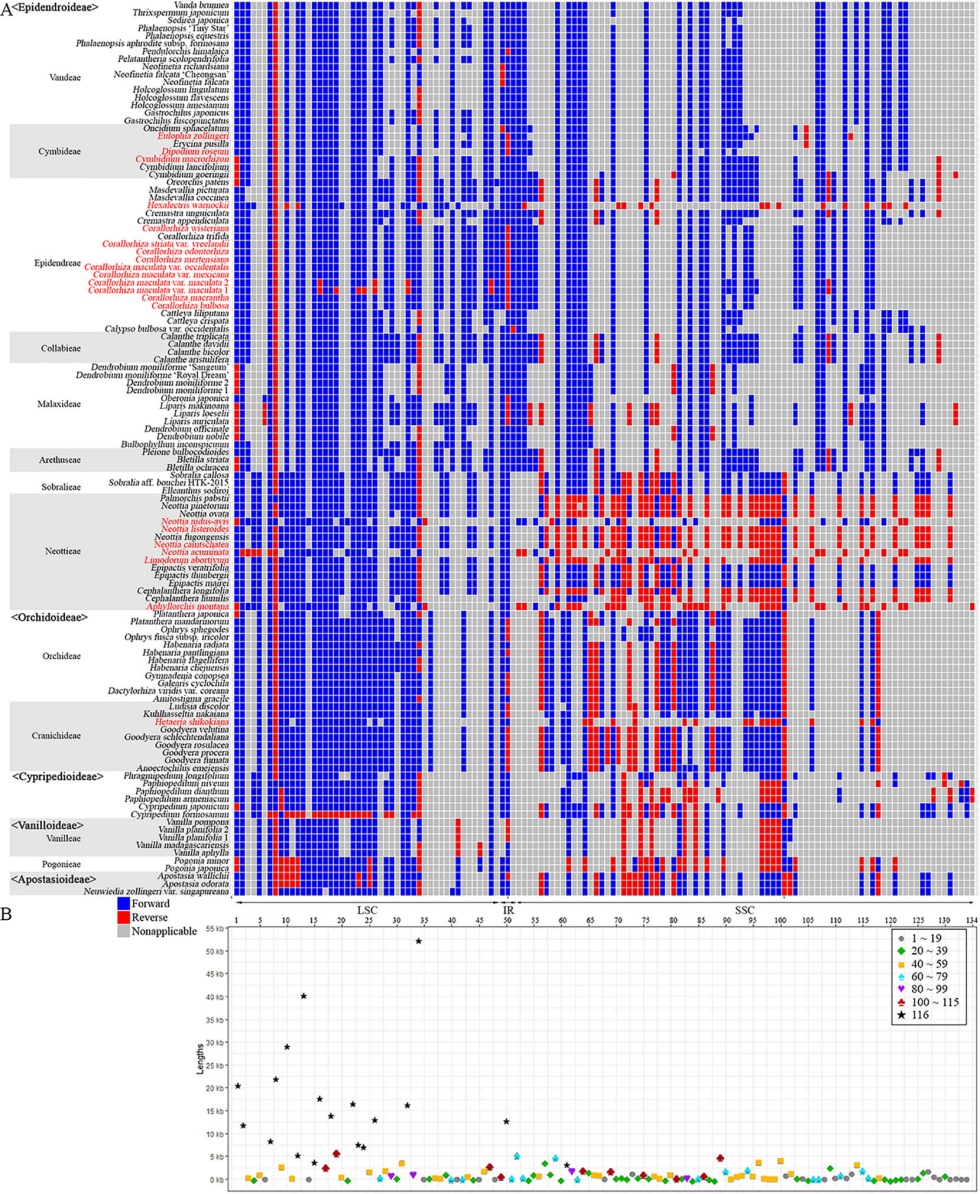
Distribution of gene blocks. A total of 134 gene blocks were recognized in the 124 orchid plastomes. Thirty were constant blocks. 104 blocks show both forward (dark blue) and reverse (red) orientations and confirmed the block rearrangements. Blocks that are non-applicable because of gene losses or shifts are indicated in gray. All large rearrangements longer than 15 kb are located on the LSC. The species names in red indicate mycoheterotrophic orchids. **(A)**: Forward and reverse orientations of each gene block in the orchid species. **(B)**: Locations, lengths, and frequencies of 134 gene blocks.

To identify gene rearrangements among Orchidaceae plastomes, gene block analysis was carried out using MAUVE. Among the 124 Orchidaceae plastomes, only 116 taxonomic groups were analyzed; eight were excluded—*Cyrtosia septentrionalis*, *Epipogium roseum*, *Epipogium aphyllum*, *Gastrodia elata* 1, *G. elata* 2, *Lecanorchis kiusiana*, *L. japonica*, and *Rhizanthella gardneri*—because they had severe gene losses and were therefore meaningless in gene block analysis. A total of 134 gene blocks were identified. The aligned lengths (kb), gene regions, forward/reverse orientations, and names of taxa distributed in individual gene blocks are listed in **Tables S1** and **S2**. In addition, the orientations of the 134 gene block and distribution patterns by taxonomic group (**Figure 8A**), and the lengths of individual blocks and the number of shared taxonomic groups (**Figure 8B**), were set forth. Among the 134 blocks, 104 were identified as genome rearrangement blocks because they showed variations, and 30 were identified as constant blocks. In terms of plastome regions, 48 blocks were found in the LSC region and among them, 34 were identified as rearrangement blocks and 14 were identified as constant blocks. Only three blocks were found in the IR region, and they were rearranged blocks. Eighty-three blocks (~60%) were found in the SSC region and 24 small blocks were identified in the *ycf*1 gene region existing at the IR/SSC boundary.

Based on block length, 81 blocks shorter than 1 kb, 35 blocks 1 to 5 kb long, six blocks 5 to 10 kb long, and 12 blocks longer than 10 kb were found. The longest gene block is 52.63 kb in length, located between *pet*D-*rps*12 in the LSC region that has rearranged. This block was followed by *trn*E-UUC-*ycf*3 (40.6 kb, rearrangement), *rpo*C2-*rpo*B (29.49 kb, rearrangement), *trn*G-UUC-*rpo*C2 (22.42 kb, rearrangement), *psb*A-*trn*K-UUU (21.00 kb, rearrangement), *cem*A-trnP-UGG (18.15 kb, rearrangement), *trn*V-UAC-*atp*B (16.92 kb, rearrangement), *psb*B-*pet*D (16.64 kb, rearrangement), *trn*L-UAA-*ndh*J (14.42 kb rearrangement), *psa*J-*clp*P (14.42 kb, rearrangement), *rrn*16-*rrn*5 (13.19 kb, rearrangement), and *rps*16 (12.32 kb, rearrangement). Among the 12 gene blocks longer than 10 kb long, 10 are distributed in the LSC region and two in the IR region (**Figure 8B**).

In the gene block analysis, to compare structures in the vicinity of the IR/SSC junctions where many small rearrangements are concentrated, the SC-IR junctions of 42 species of Orchidaceae (including 24 new plastome sequences) decoded in this laboratory were schematized by taxonomic group and expressed as shown in **Figure S2**. Here, the IR of *Gastrodia elata* was deleted. All the LSC-IRb junctions of 19 taxonomic groups, not including non-photosynthetic taxonomic groups (*Cyrtosia*, *Hetaeria*, *Lecanorchis*), were formed in the vicinity of *rpl*22. The LSC-IRb junction of *Cyrtosia septentrionalis* was found in the vicinity of *rps*19, that of *Lecanorchis* was found in the vicinity of *rpl*2, and that of *Hetaeria shikokiana* was found in the vicinity of *rpl*2. As with LSC-IRb junctions, LSC-IRa junctions were identified to have been formed in the IGS before *psb*A in all taxonomic groups except for three non-photosynthetic taxonomic groups (*Hetaeria shikokiana, Lecanorchis kiusiana,* and *L*. *japonica*). The IRa-SSC and IRb-SSC junctions of the remaining taxonomic groups—except non-photosynthetic taxonomic groups, seven species of Vanilloideae (in *Cyrtosia*, *Lecanorchis*, *Pogonia*, and *Vanilla*) and one species of Orchidoideae (*Hetaeria*)—were located on *ndh*F and *ycf*1, respectively. Seven taxonomic groups belonging to Vanilloideae commonly showed a phenomenon of a shorter SSC and among them, in the case of non-photosynthetic taxonomic groups *Cyrtosia* and *Lecanorchis*, the rRNA genes generally present in the IR were relocated to the SSC. Although *Hetaeria shikokiana* belongs to Orchidoideae, it has a shortened SSC (2,320 bp), similar to the taxonomic groups of Vanilloideae (**Figure 1**).

### Phylogenetic Relationships Among the Major Lineages of Orchidaceae

The phylogenetic relationships connected to Apostasioideae {Vanilloideae [Cypripedioideae (Orchidoideae, Epidendroideae)]} were identified based on a ML tree made using 83 genes (**Figure S3**). Although bootstrap values were high at most nodes, those of the nodes of *Anoectochilus emeiensis* and *Goodyera velutina*, and *G*. *procera* and *G*. *fumata* of Cranichideae, were shown to be relatively low at 69.4% and 77.6%, respectively, as was the bootstrap value of the node dividing *Neottia camtschatea*, *N*. *listeroides,* and *N*. *nidus*-*avis* at 63.3%. The results of a BI tree made using the same data matrix are shown in **Figure S4**. The BI value of Cranichideae, which had low bootstrap values in the ML tree, showed a high degree of support at 1.0, but the BI value of *Corallorhiza*'s branches showed a degree of support of 0.5. Despite the bootstrap and BI support problems in several sub-taxonomic groups, the relationships among the tribes (Pogonieae, Vanilleae, Cranichideae, Diurideae, Orchideae, Neottieae, Sobralieae, Nervilieae, Gastrodieae, Arethuseae, Malaxideae, Collabieae, Epidendreae, Cymbidieae, and Vandeae) represented by the data matrix used were identified to be strongly supported in both the ML and BI trees. In addition, both ML and BI trees had very long branch lengths leading to the taxa with highly reduced plastomes, such as *Epipogium*, *Gastrodia*, and *Rhizanthella* (**Figures S3** and **S4**).

### Comparing Chloroplast and Nuclear Datasets

**Figure 9:**
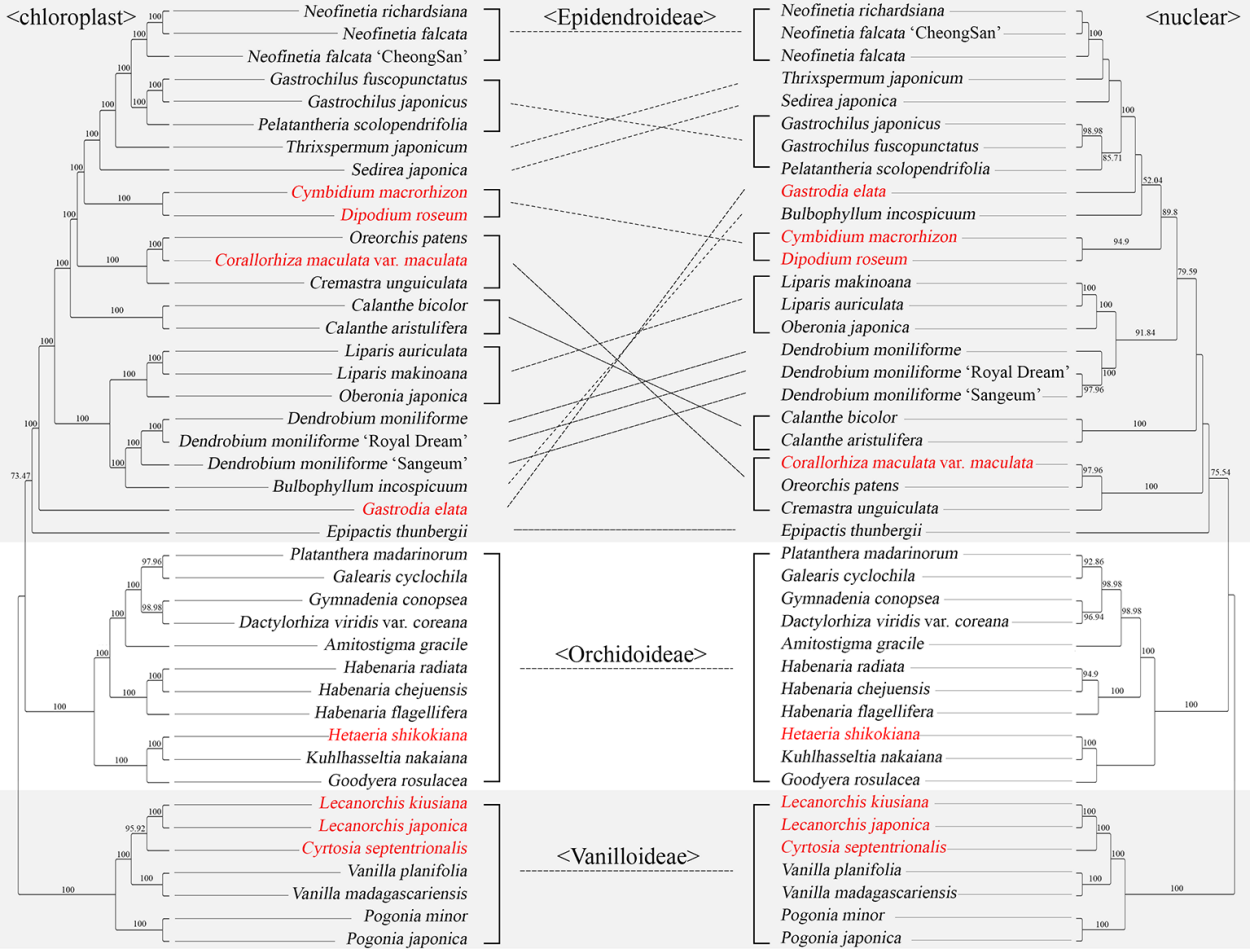
Comparison of a plastid tree and nrDNA tree for the same 42 orchid species. The 83 aligned protein coding genes were 80,798 bp long. A maximum likelihood (ML) tree was constructed using RaxML-HPC2 with a GTR+G+I model with 100 bootstrap replicates. The best plastid tree was obtained with ML = −332414.394814. The nrDNA unit (18S-ITS1-5.8S-ITS2-28S) was approximately 10 kb long. The tree reconstruction methods for nrDNA were identical to those of the plastid tree. The species names in red indicate mycoheterotrophic orchids. The lines between the two trees indicate the topological differences between them.

Among the 42 Orchidaceae species decoded in this laboratory, both plastome sequences and nrDNA units (18S-ITS1-5.8S-ITS2-28S) about 10 kb long exist (**Table 2**). Therefore, phylogenetic trees using the nrDNA regions and phylogenetic trees using the base sequences of the 83 plastid genes were made using the same method and compared (**Figure 9**). The best nrDNA tree was obtained at ML = −35673.363572 and the best plastid tree was obtained at ML = −332414.394814. These phylogenetic trees included only three subfamilies—Vanillioideae, Orchidoideae, and Epidendroideae—and the mutual relationships among the three subfamilies and the tree topologies in Vanilloideae and Orchidoideae were identical. However, differences between the two trees were found in the tree topologies in Epidendroideae. In particular, the phylogenetic positions of *Gastrodia elata*, *Bulbophyllum inconspicuum*, *Calanthe bicolor*, *C. aristulifera*, *Cremastra unguiculata*, *Corallorhiza maculata* var. *maculata*, *Oreorchis patens*, *Sedirea japonica*, and *Thrixspermum japonicum* differed between the two trees (**Figure 9**).

### Time Estimation

**Figure 10:**
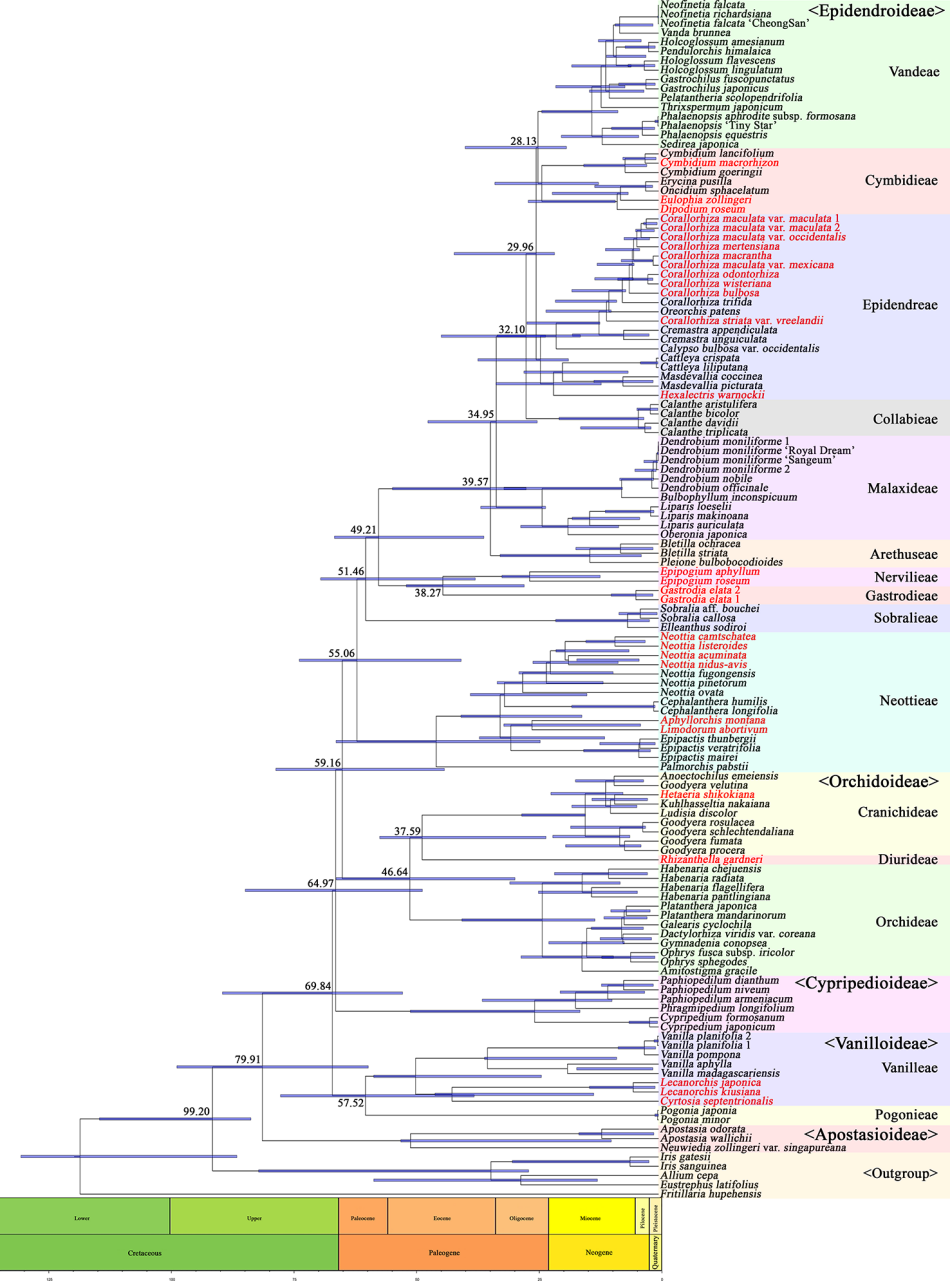
Fossil data showing the estimated divergence time of each node. Three fossil data were used to calibrate nodes (Asparagales—mean 105.3 mya, *Dendrobium*—23.2 mya, and *Goodyera*—15.0 mya). Orchidaceae diverged from its sister family at 99.20 mya, and then five subfamilies subsequently diverged in the order of Apostasioideae (79.91 mya), Vanilloideae (69.84 mya), Cypripedioideae (64.97 mya), Orchidoideae (59.16 mya), and Epidendroideae (59.16 mya). However, several specious subtribes within the Epidendroideae diverged in relatively short time periods (39.57–28.13 mya). The species names in red indicate mycoheterotrophic orchids.

A phylogenetic tree was constructed using 83 plastid genes in 124 Orchidaceae species and five outgroup species, and the divergence times of individual tree nodes were estimated using BEAST2. The results are shown in **Figure 10**. The inferred branching times of 21 main clades are set forth in **Table 4**. For easy comparison with existing studies, literature materials are also presented in **Table 4**. In this study, Orchidaceae was inferred to have diverged from other Asparagales at 99.20 (83.78–114.92) mya; Apostasioideae, which is a basal subfamily, was inferred to have diverged from other subfamilies at 79.91 (59.65–99.00) mya; and Vanilloideae was inferred to have diverged at 69.84 (52.59–89.63) mya. It was inferred that Pogonieae and Vanilleae of Vanilloideae diverged at 57.52 (37.88–77.68) mya, Cypripedioideae diverged at 64.97 (48.54–84.93) mya, and Orchidoideae diverged from Epidendroideae at 59.16 (43.99–78.66) mya. Cranichideae of Orchidoideae diverged from Orchideae at 46.64 (29.49–66.26) mya and Diurideae diverged from Cranichideae at 37.59 (23.13–57.30) mya. Neottieae diverged at 55.06 (40.55–73.82) mya and other taxonomic groups of Epidendroideae and Sobralieae diverged at 51.46 (37.65–69.44) mya. In addition, Gastrodieae and Nervilleae diverged from other Epidendroideae at 49.21 (35.89–66.60) mya, and the two tribes diverged from each other at 38.27 (27.59–51.84) mya. Arethuseae diverged at 39.57 (27.20–54.68) mya, Malaxideae at 34.95 (24.93–47.40) mya, Collabieae at 32.10 (23.20–44.64) mya, Epidendreae at 29.96 (21.36–41.98) mya, and Cymbideae and Vandeae at 28.13 (18.92–39.72) mya (**Figure 10**, **Table 4**).

**Table 4:** Comparing time estimation results with two previous studies that used two and three genes for analysis.

Node		This study (mya)	Gustafsson et al., 2010 (Two genes, stem age, mya)	Givnish et al., 2015 (Three genes, stem age, mya)
**Orchidaceae**		99.20 (83.78–114.92)	104	111.38
**Apostasioideae**		79.91 (59.65–99.00)	77	89.46
**Vanilloideae**	Vanilloideae	69.84 (52.59–89.63)	69	83.6
	Pogonieae	57.52 (37.88–77.68)	57	77.81
	Vanilleae	57.52 (37.88–77.68)	57	77.81
**Cypripedioideae**		64.97 (48.54–84.93)	71	76.43
**Orchidoideae**	Orchidoideae	59.16 (43.99–78.66)	61	63.99
	Orchideae	46.64 (29.49–66.26)	33	54.39
	Diurideae	37.59 (23.13–57.30)	48	48.24
	Cranichideae	37.59 (23.13–57.30)	48	48.24
**Epidendroideae**	Epidendroideae	59.16 (43.99–78.66)	61	63.99
	Neottieae	55.06 (40.55–73.82)	49	48.05
	Sobralieae	51.46 (37.65–69.44)	43	43.33
	Nervilieae	38.27 (27.59–51.84)	43	40.69
	Gastrodieae	38.27 (27.59–51.84)	N/A	N/A
	Arethuseae	39.57 (27.20–54.68)	33	37.86
	Malaxideae	34.95 (24.93–47.40)	35	36.56
	Collabieae	32.10 (23.20–44.64)	33	33.76
	Epidendreae	29.96 (21.36–41.98)	35	34.08
	Cymbideae	28.13 (18.92–39.72)	35	32.67
	Vandeae	28.13 (18.92–39.72)	35	32.67

## Discussion

### Evolution of the Plastome Structure

Most plastomes of flowering plants have quadripartite structures consisting of LSC and SSC regions with two IR regions between the two SCs (Bock, 2007). The IR regions are generally known to play a role in the structural stability of plastomes (Palmer and Thompson, 1982), and Orchidaceae plastomes are no exception. When the 124 Orchidaceae plastomes were compared, all but *Aphyllorchis montana*, *Epipogium aphyllum,* and *Gastrodia elata* (2 accessions) had quadripartite structures (**Table 2**). *Gastrodia elata* (Epidendroideae-Gastrodieae) is a special case in which the plastome exists only as one single copy because the IR region was lost when the plastome contracted to about 35.1 kb (**Figure 1**). In the case of *Aphyllorchis montana* (Epidendroideae-Neottieae), the plastome also exists as only one single copy region because the IR region was lost when the plastome size decreased to 94.6 kb (Feng et al., 2016). In the case of *Epipogium aphyllum* (Epidendroideae-Nervilieae), the plastome has a tripartite structure consists of the SC and IR because the two IR regions were combined to head-to-head formation when the SSC region disappeared and the plastome contracted to 30.6 kb (Schelkunov et al., 2015). However, in the case of *Epipogium roseum* in the same genus, the SSC region exists at 890 bp (shorter than average) and the IR decreased to 261 bp (**Figure 3**). Although these structural variations are related to gene losses associated with mycoheterotrophic habitats, the phenomenon of broken quadripartite structures appears only in the three species mentioned above of the 28 mycoheterotrophic species compared. Plastomes exist as only SC regions due to the loss of IR in several gymnosperms (Wu et al., 2011), *Trifolium* (Fabaceae), *Medicago* (Fabaceae), and *Cicer* (Fabaceae) (Cai et al., 2008). However, all these are photosynthetic species, which are different from the Orchidaceae mentioned above.

Another feature that can be found frequently in Orchidaceae plastomes is IR expansion/shift toward the SSC region. Therefore, the SSC size has been greatly reduced. Due to this feature, which can be found in *Pogonia* and *Vanilla* of Vanilloideae, the lengths of IR became 29,808–32,683 bp because both IRa and IRb expanded toward the SSC (Lin et al., 2015; Amiryousefi et al., 2017). In contrast, the lengths of the SSC were shortened by as much as 1,254–5,387 bp. The similar SSC contractions were also found in three species of Cypripedioideae: *Paphiopedilum armeniacum*, *P*. *dianthum*, and *P*. *niveum* (**Table 2**). The same phenomenon was observed in the newly decoded plastome of *Hetaeria shikokiana*, and the length of the IR and the SSC were found to be 31,960 bp and 2,320 bp, respectively (**Figure 1**). Among the four genera in which SSC contraction occurred, *Paphiopedilum*, *Pogonia,* and *Vanilla* carry out photosynthesis, while *Hetaeria* does not. As for life forms, three genera (*Hetaeria*, *Paphiopedilum*, and *Pogonia*) have terrestrial life forms, while one genus (*Vanilla*) has an epiphytic life form. In all four genera, *ndh* genes were deleted in most cases. This is thought to be a widely occurring phenomenon in orchids due to parallel evolution and is not considered attributable to the direct effect of SSC contraction. Since *ndh* genes exist in the vicinity of IR-SSC—such as *ndh*A, *ndh*B, and *ndh*H in *Pogonia* and *Hetaeria*, which are pseudogenes—*ndh* genes are not a direct cause of SSC contraction. However, many small sequence block rearrangements appear in many Orchidaceae lineages near the IR-SSC boundary, indicating that the *ndh*F and *ycf*1 genes at the IR-SSC boundary may affected the stability of Orchidaceae plastomes. This is a common phenomenon, especially in species that have lost their *ndh* gene class or the capacity for photosynthesis.

On the other hand, no IR shift toward the IR-LSC is observed in the Orchidaceae plastomes (**Figure S2**). The largest plastome size in Orchidaceae was 178,131 bp in *Cypripedium formosanum*, but there was no IR expansion toward the LSC in this case either. The increase in plastome sizes in photosynthetic plant species is mainly related to IR expansion (Chumley et al., 2006; Weng et al., 2014; Blazier et al., 2016). However, in the case of *Cypripedium*, the genome size increased due to scattered AT-rich repeats among IGS in the LSC region without IR expansion (**Figure S2**) (Kim et al., 2015b). Mycoheterotrophic orchids have plastome sizes below 150 kb and show high correlations between length of the LSC region and the IR length (R^2^ = 0.9571 and R^2^ = 0.8213, respectively). On the other hand, the plastome sizes of all photosynthetic orchids were at least 140 kb and showed a weak correlation with the length of the LSC region (R^2^ = 0.5606), but little correlation with the IR length (R^2^ = 0.1251) (**Figures 4C, D**). This means that, in mycoheterotrophic orchids, genome contraction occurs regardless of region, but genome size expansion is affected more by the expansion of the LSC region than the expansion of the IR region.

Gene block analysis was conducted using MAUVE to identify gene rearrangements among Orchidaceae plastomes and, according to the results, 104 out of 134 blocks had rearrangements in at least one taxonomic group (**Figure 8**, **Tables S1** and **S2**). Of the 104 rearrangements, 34 were distributed in the LSC region, three were distributed in the IR region, and the remaining 67 were concentrated on the SSC region. Among the LSC rearrangements, 11 were at least 10 kb long. On the other hand, the SSC rearrangements were mostly concentrated on *ndh*F and *ycf*1 in the IR/SSC boundary region, and most were short rearrangements that did not exceed 1 kb (**Figure 8**, **Table S2**). Many of the IRa-SSC junction rearrangements are assumed to be attributable to the unequal crossing over among repeats during the processes of pseudogenization and gene loss of *ndh*F and *ycf*1. In the IR region, IR shift occurred as the genes in the SSC region were lost and became smaller, and the region containing the *rrn*4.5-*rrn*23 gene moved to the SSC region. Their position changes have been reported in mycoheterotrophs with large gene losses, such as *Aphyllorchis montana*, *Cyrtosia septentrionalis*, *Hexalectris warnockii*, *Lecanorchis japonica*, *L*. *kiusiana,* and *Rhizanthella gardneri* (Delannoy et al., 2011; Feng et al., 2016; Barrett and Kennedy, 2018; Kim et al., 2019). Given that the relevant shift did not occur in other mycoheterotrophs (*Corallorhiza*, *Dipodium*, *Hetaeria*, or *Neottia*), this is considered to be an independent evolutionary phenomenon associated with gene loss.

Based on taxonomic groupings, although some species in the genera that include both photosynthetic and non-photosynthetic nutritional modes such as *Cephalanthera*, *Cymbidium*, *Epipactis*, and *Platanthera* (Merckx et al., 2013) shared rearrangements, species of other genera in the same category (e.g., *Cremastra*) did not share any rearrangements, indicating that the gene relocation in the plastome occurred after development of non-photosynthetic mycoheterotrophy. Therefore, the plastid gene rearrangements are judged to have developed independently in many orchid lineages, and this is supported by the fact that each of photosynthetic groups (*Apostasia*, *Bulbophyllum*, *Dendrobium*, *Elleanthus*, *Oberonia*, *Ophrys*, *Palmorchis*, *Pleione*, *Pogonia*, *Sobralia*, and *Vanilla*) has multiple rearrangements. Furthermore, since a number of rearrangements have been reported in *Aphyllorchis montana*, *Cyrtosia septentrionalis*, *Hexalectris warnockii*, *Lecanorchis japonica*, *L. kiusiana,* and *Rhizanthella gardneri*, which are mycoheterotrophs that reached stage 2 of gene loss, it is assumed that rearrangements played some role in gene loss. However, given that there is no gene rearrangement in mycoheterotrophs (*Corallorhiza*, *Hetaeria*, and *Neottia*) (Delannoy et al., 2011; Feng et al., 2016; Barrett and Kennedy, 2018; Kim et al., 2019), whose gene losses reached stage 1 or 2, further comparative studies are necessary to determine the relationships between gene relocation and gene loss.

### Evolution of Mycoheterotrophy and Gene Loss in Orchidaceae

Almost all orchid species are initially mycoheterotrophs at an early stage of development, then subsequently develop into full autotrophs. Some orchids exhibit both autotrophic and mycoheterotrophic (mixotrophic) nutritional modes, even at adult stages. Finally, several orchid species maintain an obligate (full) mycoheterotrophic (non-photosynthetic) nutritional mode throughout their life cycle. Forty-three genera in Orchidaceae are known to include non-photosynthetic mycoheterotrophs (Merckx et al., 2013). Among them, 17 were used in this study (*Aphyllorchis*, *Cephalanthera*, *Corallorhiza*, *Cremastra*, *Cymbidium*, *Cyrtosia*, *Dipodium*, *Epipogium*, *Eulophia*, *Gastrodia*, *Hetaeria*, *Hexalectris*, *Lecanorchis*, *Limodorum*, *Neottia*, *Platanthera*, and *Rhizanthella*), and only photosynthetic species were analyzed in the case of three of these genera (*Cephalanthera*, *Cremastra*, and *Platanthera*). However, when the plastomes were checked, it was found that *Cymbidium macrorhizon*, *Eulophia zollingeri*, *Dipodium roseum*, and *Limodorum abortivum* only lost the *ndh* gene (**Figure 5**, **Figure S1**). In addition, after many literature reviews and firsthand observation by these researchers, it was found that, regarding these four species, chlorophylls exist in stems and flowers and low-level photosynthesis is carried out during a certain period in the life cycle (Blumenfeld, 1935; Girlanda et al., 2006; Kim et al., 2017a; Suetsugu et al., 2018). Therefore, it is reasonable to treat these four species as mixotrophs rather than obligate mycoheterotrophs. Among the species thought to be non-photosynthetic, *Corallorhiza*, which was studied the most extensively, is very likely to have photosynthetic capability if only *ndh* genes had been lost. In addition, cases have been reported in which members of the same species of *Corallorhiza* had different, mixotrophic nutritional modes depending on living environments (Barrett and Davis, 2012; Barrett et al., 2014; Barrett et al., 2018). The estimated divergence times of these mixotrophs are 7.44 (2.41–15.37) mya (*Cymbidium*), 17.34 (8.87–26.76) mya (*Dipodium* and *Eulophia*), and 17.77 (3.68–31.79) mya (*Limodorum*), and the evolution of these genera are thought to be relatively recent events. Furthermore, the divergence times of the obligate mycoheterotrophic groups were also estimated as follows: 23.61 (16.05–33.12) mya (*Corallorhiza* and *Cremastra*), 21.56 (11.77–33.40) mya (*Hexalectris*), 38.27 (27.60–51.84) mya (*Gastrodia* and *Epipogium*), 28.11 (15.75–40.61) mya (*Aphyllorchis*, *Cephalanthera*, *Epipactis,* and *Neottia*), 5.32 (1.71–9.80) mya (*Platanthera*), 37.59 (23.13–57.30) mya (*Rhizanthella*), 7.48 (2.26–13.66) mya (*Hetaeria*), and 27.82 (13.31–45.95) mya (*Cyrtosia* and *Lecanorchis*) (**Figure 10**). It can be seen that, except for Gastrodieae (*Gastrodia*), Nervilleae (*Epipogium*), and Diurideae (*Rhizanthella*), all these species diverged more recently than 30 mya (**Figure 10**). When estimated based on the foregoing, there are two possibilities: the obligate mycoheterotrophic taxa are relatively recently evolved taxonomic groups within Orchidaceae, and information about the older obligate mycoheterotrophic taxonomic groups is not yet available. To test these, further studies should be conducted on other species in Gastrodieae and Nervilleae, whom are estimated to have diverged for long times ago.

While epiphytic orchids consisted only of photosynthetic species and all had genomes at least 140 kb long, terrestrial orchids showed a large variation in plastome sizes and had much different numbers of functional genes because they included non-photosynthetic species (**Figure 4A**). In addition, the correlation between genome size and the number of functional genes was high (R^2^ = 0.9069) in mycoheterotrophs, and relatively low (R^2^ = 0.3320) in photosynthetic species (**Figure 4B**). Also, mycoheterotrophs are distributed into three clusters: one with about 100 functional genes, one with 55–90 functional genes, and one with 25–30 functional genes (**Figures 4B** and **5**). This means that in the evolutionary process of mycoheterotrophic species, gene loss occurred step by step in three stages rather than on a continuous spectrum.

The first stage of gene loss was the pseudogenization or loss of the *ndh* gene class. There are 11 *ndh* gene subunits in each plastome. *ndh* gene losses are observed in all five subfamilies of Orchidaceae, and appear at especially high frequencies in Cypripedioideae, Epidendroideae, and Vanilloideae. On the other hand, the frequencies of non-functionalization are low in Apostasioideae and Orchidoideae. The *ndh* genes of the epiphytic orchids (*Bulbophyllum*, *Cattleya*, *Dendrobium*, *Erycina*, *Gastrochilus*, *Holcoglossum*, *Neofinetia*, *Oberonia*, *Phalaenopsis*, *Pelatantheria*, *Pendulorchis*, *Sedirea*, *Thrixspermum*, *Vanda*, and *Vanilla*) used in the analysis were lost or pseudogenized (**Figures 5** and **6**). Although the loss or pseudogenization of *ndh* genes was also observed in terrestrial taxonomic groups (*Apostasia*, *Calypso*, *Cephalanthera*, *Cremastra*, *Cymbidium*, *Epipactis*, *Goodyera*, *Kuhlhasseltia*, *Limodorum*, *Liparis*, *Neuwiedia*, *Oncidium*, *Paphiopedilum*, *Phragmipedium*, *Platanthera*, and *Pogonia*), the frequencies are low compared to epiphytes. Although the loss or pseudogenization of *ccs*A, *cem*A, and *inf*A is often observed in addition to *ndh* gene non-functionalization, but no clear trend has been found (Barrett et al., 2014; Kim et al., 2015a; Feng et al., 2016; Niu et al., 2017b; Hou et al., 2018). In the phylogenetic tree, the non-functionalization of *ndh* appears to occur gradually and independently in the process of pseudogenization in many independent lineages (**Figure 6**). However, given that most orchid species in which *ndh* gene non-functionalization occurred retain photosynthetic capacity, it is inferred that the function of this gene class may be affected by the nuclear or mitochondrial genomes. Of course, the gene function may be maintained by RNA editing after pseudogenization, but the possibility is low because most pseudogenization entail not only base changes but also indels. However, given that *ndh* genes were lost in all non-photosynthetic species, the loss of the *ndh* gene class is considered to be a precondition for the development of mycoheterotrophs.

The second stage of gene loss is the loss of functions of genes involved in the photosynthetic light reaction, such as *pet* (six genes), *psa* (five genes), and *psb* (15 genes). This includes the *rpo* (four genes) gene class, which includes housekeeping genes. Gene losses at this stage are shown to have progressed independently in at least 10 clades including six independent lineages of Epidendroideae (*Aphyllorchis, Corallorhiza*, *Epipogium*, *Gastrodia*, *Hexalectris,* and *Neottia*), two Orchidoideae clades (*Hetaeria* and *Rhizanthella*), and one Vanilloideae clade (*Cyrtosia* and *Lecanorchis*) (**Figure 6**). In particular, in the case of *Corallorhiza* and *Neottia*, gene losses progressed independently depending on species, even within the same genus, indicating that these are important taxonomic groups for understanding second stage gene losses. In addition, the plastomes of *Cyrtosia* and *Lecanorchis* of Vanilloideae degraded significantly so that *ccs*A, *cem*A, *rbc*L, *ycf*3, and *ycf*4 were commonly deleted in addition to *ndh*, *pet*, *psa*, *psb*, and *rpo*, and it was identified that some subunits of the housekeeping genes, such as *rpl* and *rps,* were also deleted from *Lecanorchis* (**Figure 6A**). The degradation of *Cyrtosia* and *Lecanorchis* of Vanilloideae is thought to be part of the transition process from plastome degradation stage 2–4 (Wicke et al., 2016). On the other hand, *Hetaeria shikokiana* of Orchidoideae and *Corallorhiza maculata* var. *maculata* of Epidendroideae correspond to plastome degradation stage 2 because some of their *ndh*, *psa*, *psb*, *pet,* and *rpo* genes were deleted or pseudogenized. However, since plastome degradation stages 2 and 3 (Wicke et al., 2013; Wicke et al., 2016) appeared to occur simultaneously in Orchidaceae, this stage was defined as the second stage of gene loss.

The third stage of gene loss in Orchidaceae includes cases where only 27 to 33 out of 113 unique plastome genes remain, meaning that further gene loss has occurred than in stage 2 cases. Since at least 55 genes are preserved in stage 2 and fewer than 33 genes are preserved in stage 3, a large gap exists between the two stages (**Figure 4B**). In stage 3, all the photosynthesis-related gene functions were lost and most housekeeping genes—such as *trn*, *rpl,* and *rps*—were also lost. In addition, the fact that pseudogenes do not exist or are limited to three or fewer is also a characteristic of stage 3. This stage is observed in a total of four lineages: two lineages of Epidendroideae (*Epipogium*, 27 to 29 conserved genes; *Gastrodia*, 28 conserved genes), one of Orchidoideae (*Rhizanthella*, 33 conserved genes), and one of Vanilloideae (*Lecanorchis*, 32–33 conserved genes) (**Figures 5** and **6**, **Table 2**). Regarding the number of genes, the 27 in *Epipogium aphyllum* are the minimum number of genes found in Orchidaceae plastomes. Furthermore, 20 genes that commonly exist in the four lineages of seven plastomes, which are in gene loss stage 3, were identified, comprising *rpl* (12, 14, 16, 36), *rps* (2, 3, 4, 7, 8, 11, 14, 18, 19), *rrn* (5, 16, 23), *trn*C-GCA, *trn*fM-CAU, *acc*D, and *clp*P (**Table 3**). The fact that the same 20 genes have been preserved even though the extremely contracted orchid plastomes evolved from four independent mycoheterotrophic lineages means that those genes selectively remained because they perform the minimum functions necessary to maintain the plastomes. Since five (*rpl*12, *rps*18, *rps*19, *trn*fM-CAU, and *acc*D) of these 20 genes were lost in one to four other orchid species (**Table 3**), it was concluded that there are 15 common orchid plastome genes.

### Comparisons of Plastome and nrDNA Trees

Thus far, phylogenetic studies using a portion of the plastome genes have been common (Cameron et al., 1999; Givnish et al., 2015), and phylogenetic studies using the entire plastome genes have also progressed thanks to the development of NGS technology (Yang et al., 2013; Kim et al., 2015a; Feng et al., 2016). The use of chloroplast genes in phylogenetic studies has many advantages such as ease of use, but this has been pointed out to be vulnerable to lineage sorting and problems such as plastome capture due to hybridization because it only tracks maternal lineages. Although NGS technology and transcriptome analysis are developing, there are still cost limitations to using the entire nuclear genome for phylogenetic studies. Therefore, nuclear ribosomal ITS regions have been extensively used in studies of the relationships between species or allied genera. An advantage of the genome skimming NGS is that, for plastome sequences with high depths of coverage (see **Table 1**), it can decode about 10 kb of regions that exist as tandem repeats (18S-ITS1-5.8S-ITS2-28S-NTS-ETS) (**Figure 2**). Although it has difficulties in recovering entire NTS-ETS when the plastome coverage depth is below then 500x, genome skimming for NGS can easily recover the 6 kb region of the 18S-ITS1-5.8S-ITS2-28S region. Therefore, in this study, an attempt was made to compare phylogenetic trees constructed using all the same plastome genes and phylogenetic trees constructed using the nrDNA unit sequences. Orchid plastomes from 42 species, representing three subfamilies of Orchidaceae, were produced in the laboratory of the corresponding author and directly compared (**Figure 9**).

According to the results, the relationships among the three subfamilies were the same as (Vanilloideae (Orchidoideae, Epidendroideae)). Furthermore, the associations among the tribes and species in Vanilloideae and Orchidoideae were completely identical. However, the topologies of the two trees were different for the relationships among Epidendroideae taxa. In the Epidendroideae, there was no difference in generic or tribal grouping, but the positions of *Gastrodia,* in which the plastome reduced greatly, and *Bulbophyllum*, which is a photosynthetic species, were very different. In addition, clades with bootstrap support values of less than 50% had different topologies. However, it is not clear from this comparative analysis alone whether these differences are due to lineage sorting, ancient plastome capture, other evolutionary histories, or sampling issues. Although plastome data for diverse orchid species are currently known, the sequence data for nrDNA repeats for the 42 species in this study are presented for the first time. Although some nodes in Epidendroideae show discrepancies, major parts of the trees are generally identical to each other. In addition, these data are thought to be easily obtainable because nrDNA repeats can be recovered by simply carrying out additional data mining while conducting plastome sequencing using the genome skimming NGS method. If more data are accumulated for the same sample, then the evolution of orchids can be more easily understood.

### Diversification of Major Lineages of Orchidaceae

The results of estimating the divergence times of major clades of Orchidaceae using all 83 genes in the plastome were similar to the findings of previous studies conducted using two or three genes at some points, and different at others (**Figure 10**, **Table 4**). First, in this study, the time of origin of orchids was estimated to be 99.20 (83.78–114.92) mya, which is similar to the previous estimate of 104 mya using two genes, and not significantly different from the 111.38 mya estimated using three genes (Gustafsson et al., 2010; Givnish et al., 2015). However, the finding in this study shows the divergence time of the basal taxonomic groups ranging from Orchidoideae and Cypripedioideae to Epidendroideae to be a little earlier than the previous finding estimated using two genes (1.84–6.03 mya differences), and a little more recent than the result using three genes. That is, the times estimated in the two previous studies are very different and the one in this study fell between the other two. However, the origin time of terminal tribes in Epidendroideae was inferred to be more recent by this study than the previous studies, which only used two or three genes.

Orchidoideae is estimated to consist of 198 genera and 4,931 species, and the Epidendroideae is estimated to consist of 505 genera and 20,606 species (Chase et al., 2015; Christenhusz and Byng, 2016). These two clades account for most of Orchidaceae and diverged 71.85 mya (**Figure 10**, **Table 4**). The divergence times of Arethuseae (25 genera, 723 species), Collabieae (20 genera, 453 species), Cymbideae (165 genera, 3,997 species), Epidendreae (99 genera, 6,935 species), Malaxideae (16 genera, 4,631 species), Podochileae (27 genera, 1,292 species), and Vandeae (136 genera, 2,340 species)—tribes of Epidendroideae consisting of many species—are later than 39.57 (27.20–54.68) mya, which is relatively recent. These data mean that many species differentiated in a short time. In studies that analyzed diversification rates, the relevant tribes had higher rates compared to the basal lineage in most cases, thereby supporting the hypothesis that many species underwent evolutionary radiation in a short time (Givnish et al., 2015).

### Perspective

Sixty genera, 140 species, 146 accessions of Orchidaceae plastomes have been completed decoded and can be used in comparative studies of plastomes, including the 24 plastomes newly reported in this study (NCBI database, June 30, 2019). In the present study, the evolutionary trends of plastomes were compared and analyzed to understand the evolutionary processes of orchid plastomes and mycoheterotrophic orchids. Only three different types of species were selected in the case of *Cymbidium* (9 spp.), *Dendrobium* (40 spp.), and *Holcoglossum* (11 spp.) because many species in these genera have been studied. Therefore, the plastomes of 60 genera, 118 species, and 124 accessions were analyzed. In addition, for the 42 species decoded in the corresponding author's laboratory, a plastome tree and nrDNA tandem repeat tree were compared to discuss evolution. Orchidaceae is known to include about 736 genera and 28,000 species. Therefore, those genera for which their plastomes have been decoded thus far make up approximately 8% of all the known genera of the Orchidaceae, and those species whose plastomes have been decoded thus far comprise less than 1% of all the known species of the Orchidaceae. There are 43 genera known to include non-photosynthetic mycoheterotrophs in Orchidaceae. In this study, plastome gene loss patterns of 17 genera that correspond to 40% of the foregoing genera were compared and analyzed to derive third-stage gene losses and 15 common genes. However, if further studies are conducted with more genera and species, Orchidaceae plastome gene losses can be understood better. In particular, genera such as *Corallorhiza* and *Neottia*, in which photosynthetic species coexist with non-photosynthetic mycoheterotrophs, should be intensively studied with all species, including several populations per species. Studies of the taxonomic groups in which many species were recently differentiated should be continuously conducted to elucidate the mechanism of evolutionary radiation. In this regard, the use of both plastid and nuclear genomes using NGS technology will increase for orchid evolution studies. If high-quality NGS data are accumulated by 50% at the genus level and 10% at the species level, then the evolution of orchids can be better understood.

## Data Availability Statement

The datasets generated for this study can be found in the National Center for Biotechnology Information (NCBI), please find accession numbers in **Table 2**.

## Author Contributions

K-JK and MK designed research. Y-KK, SJ, S-HC, J-RH, and MK collected the research materials. Y-KK and SJ performed research. Y-KK, SJ S-HC, J-RH, and M-JJ analyzed data and deposited the data to data libraries. Y-KK and K-JK wrote the manuscript. MK and K-JK secured the research funds.

## Funding

This work was supported by the National Research Foundation of Korea (NRF) under grant no. NRF-2015M3A9B8030588 to K-JK and by the National Institute of Biological Resources (NIBR) under the genetic evaluation of vascular plants IV-1 (2018, grant no. NIBR201803102) to MK and K-JK.

## Conflict of Interest

The authors declare that the research was conducted in the absence of any commercial or financial relationships that could be construed as a potential conflict of interest.
