# Corrigendum: Plastome Evolution and Phylogeny of Orchidaceae, With 24 New Sequences

**DOI:** 10.3389/fpls.2020.00322

**Published:** 2020-03-20

**Authors:** Young-Kee Kim, Sangjin Jo, Se-Hwan Cheon, Min-Jung Joo, Ja-Ram Hong, Myounghai Kwak, Ki-Joong Kim

**Affiliations:** ^1^Division of Life Sciences, Korea University, Seoul, South Korea; ^2^Department of Plant Resources, National Institute of Biological Resources, Incheon, South Korea

**Keywords:** Orchidaceae, plastome evolution, gene loss, IR contraction/expansion, genome rearrangement

In the original article, there was a mistake in the legend for Figure 4 as published. The word “**terrestrial**” at the end of the legend for Figure 4A should be “**epiphytic**.” The correct legend appears below.

Figure 4. Relationships between plastome lengths and gene numbers. **(A)**: Terrestrial orchids show a wider range of variation than epiphytic orchids. **(B)**: Mycoheterotrophic orchids show a wider range of variation than photosynthetic orchids. **(C,D)**: Plastome lengths are more strongly correlated with LSC lengths than IR lengths in both mycoheterotrophic and photosynthetic orchids.

The authors apologize for this error and state that this does not change the scientific conclusions of the article in any way. The original article has been updated.

