# Supplementary material for: Plastome Evolution and Phylogeny of Orchidaceae, With 24 New Sequences
Source: Front Plant Sci. 2020 Feb 21;11:22. doi: 10.3389/fpls.2020.00022 (PMC7047749; doi:10.3389/fpls.2020.00022)

Epidendroideae
Collabieae

Supplementary Figure S1. Plastome circle map of 24 newly sequences Orchidaceae species.

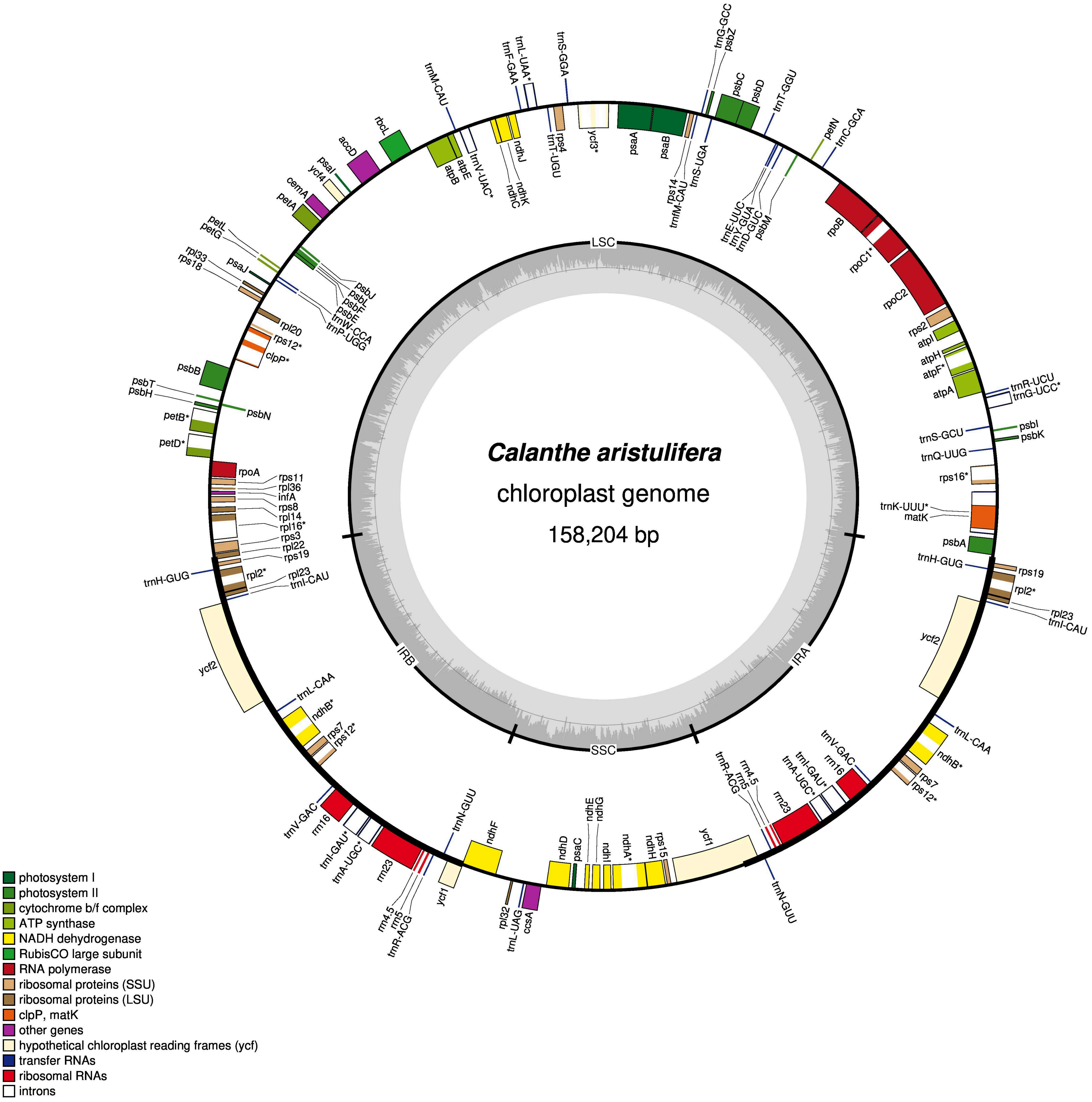

Epidendroideae
Collabieae

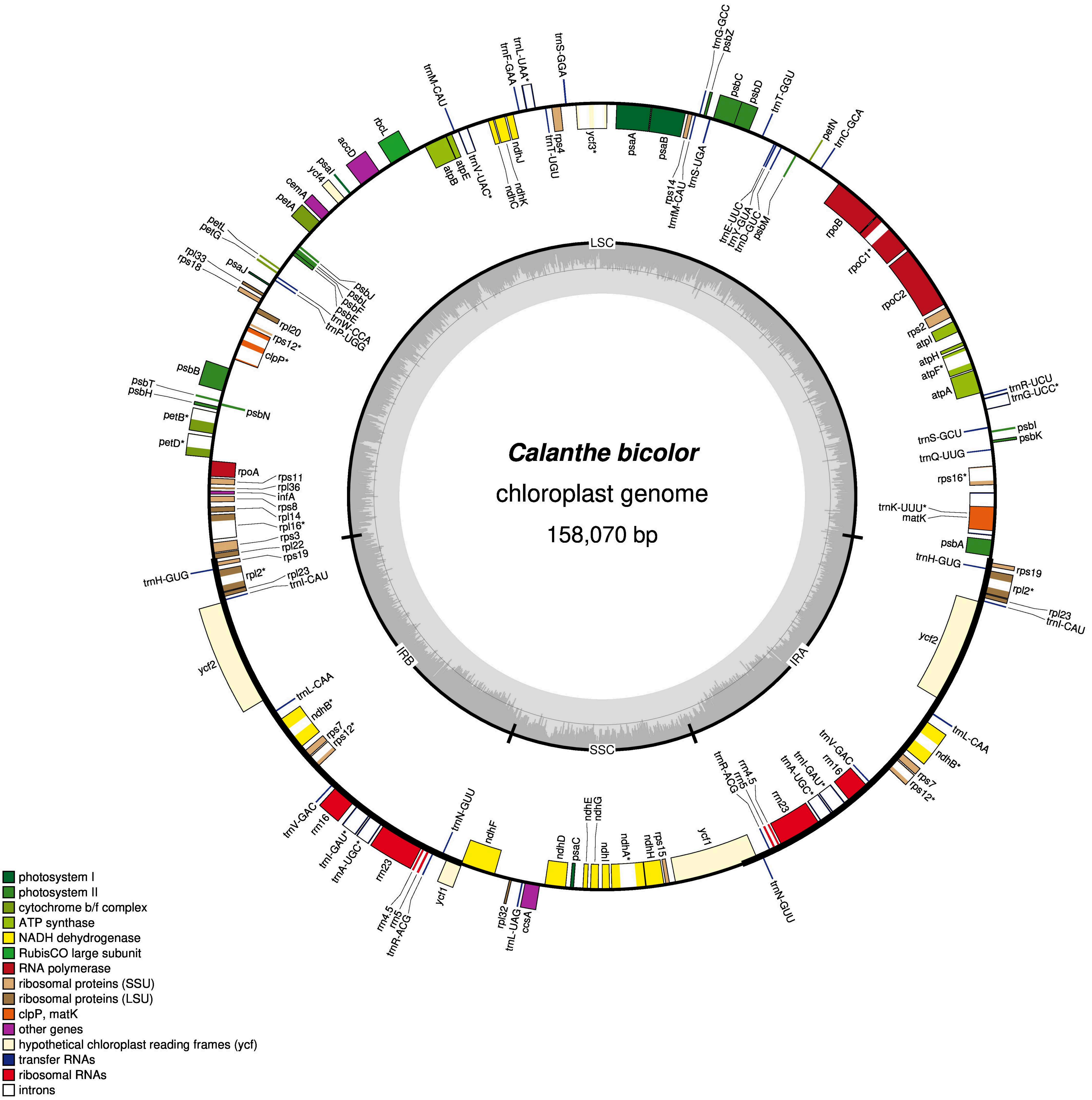

Epidendroideae
Cymbidieae

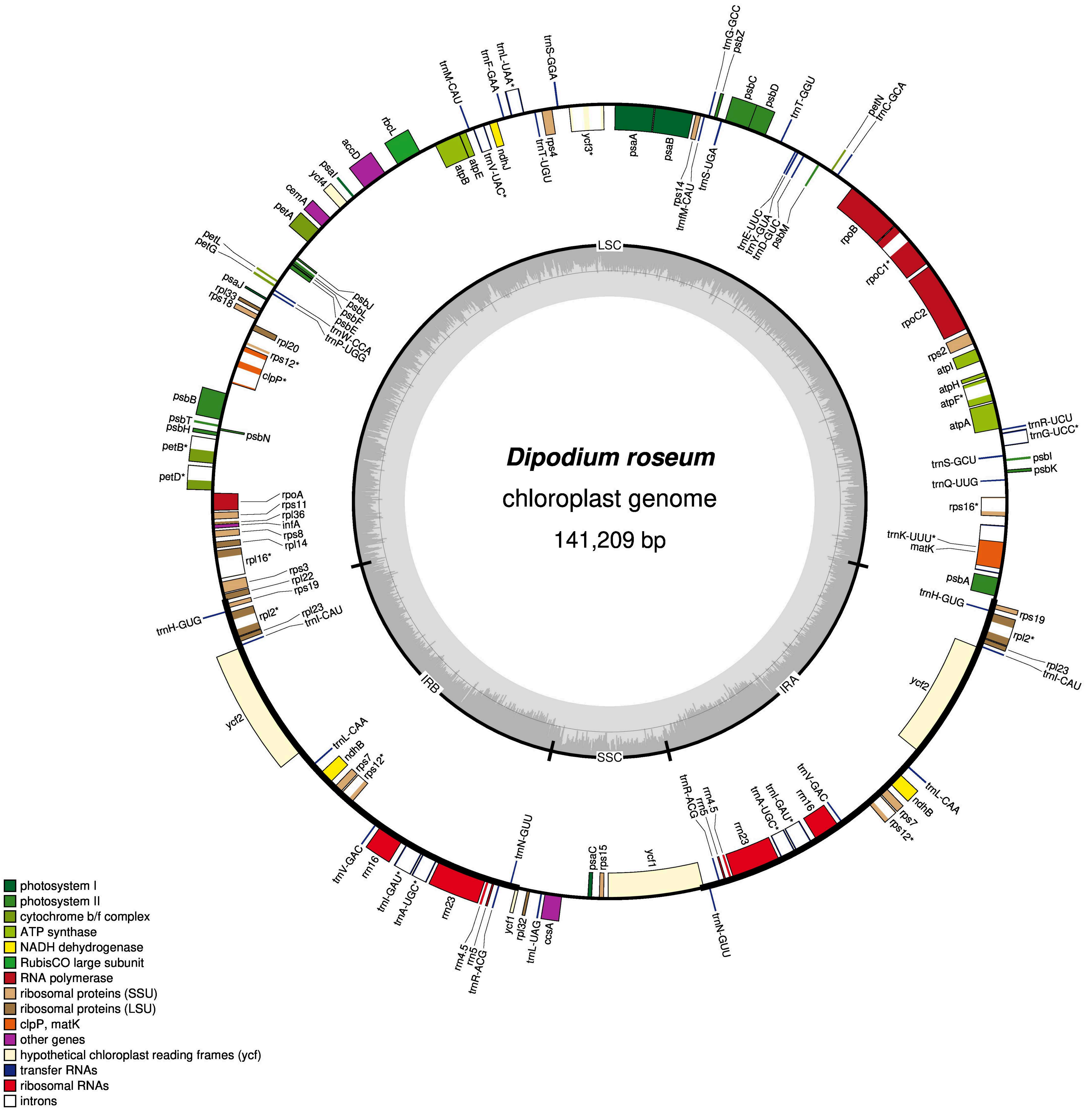

Epidendroideae
Epidendreae

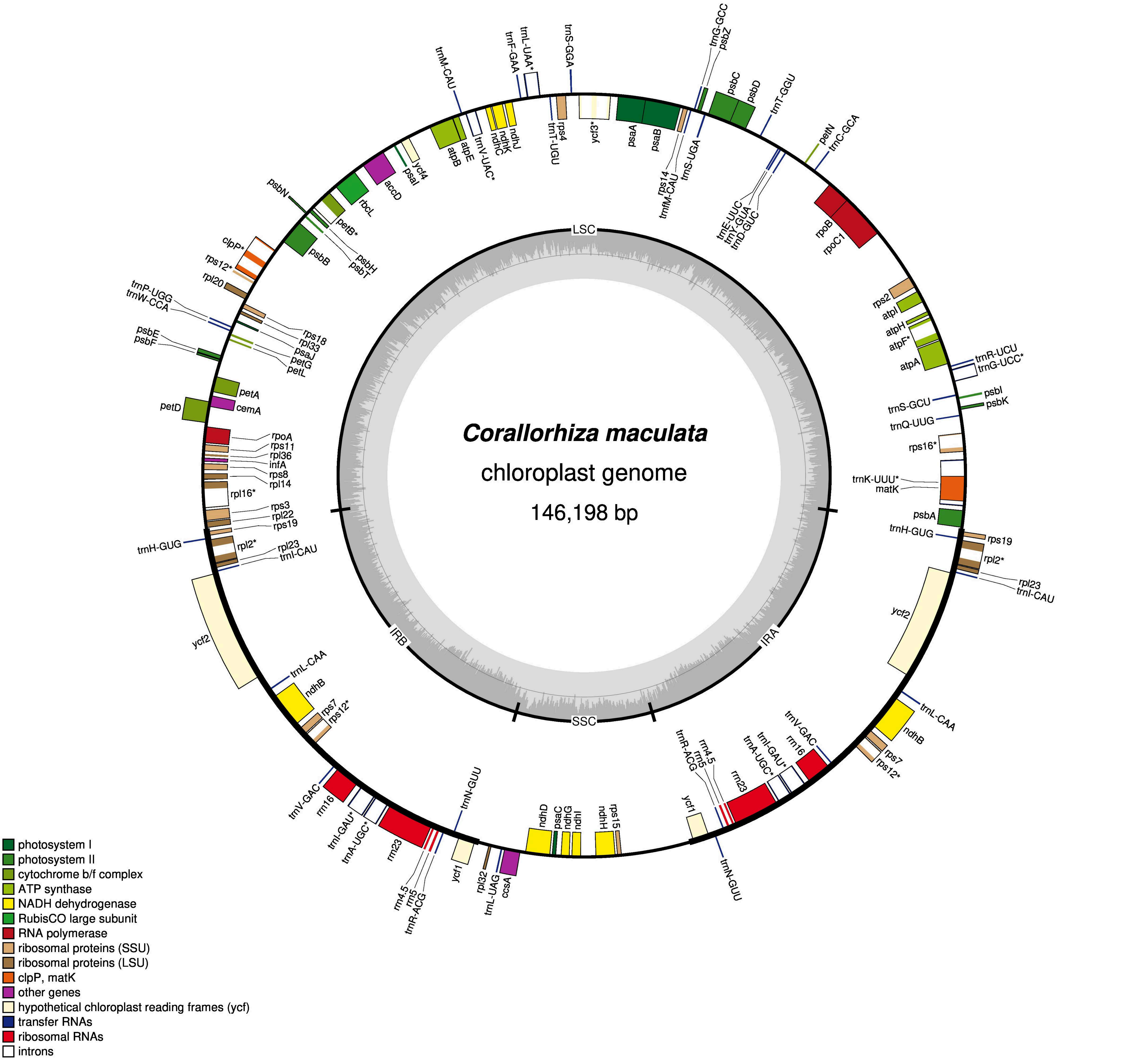

Epidendroideae
Epidendreae

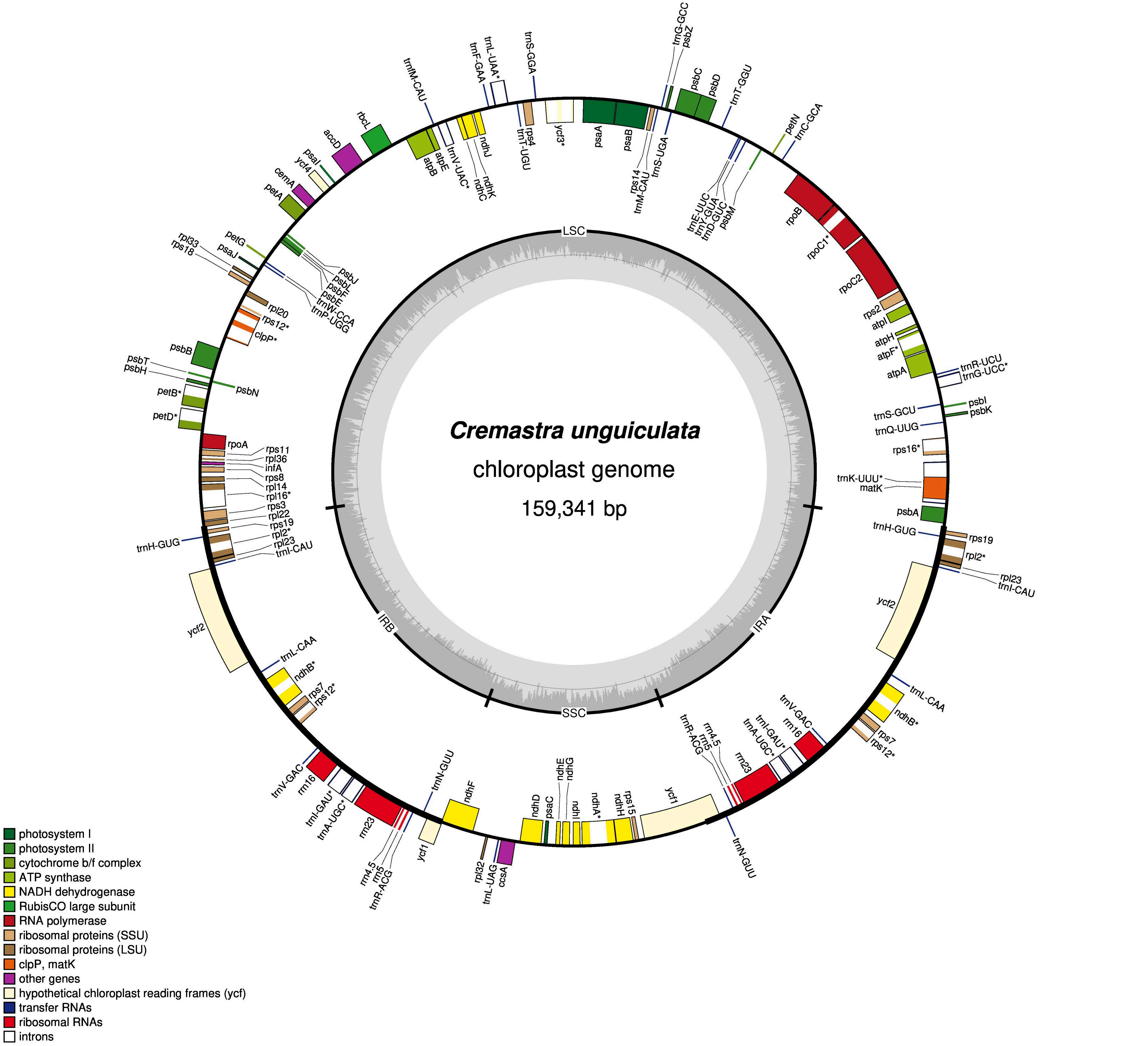

Epidendroideae
Epidendreae

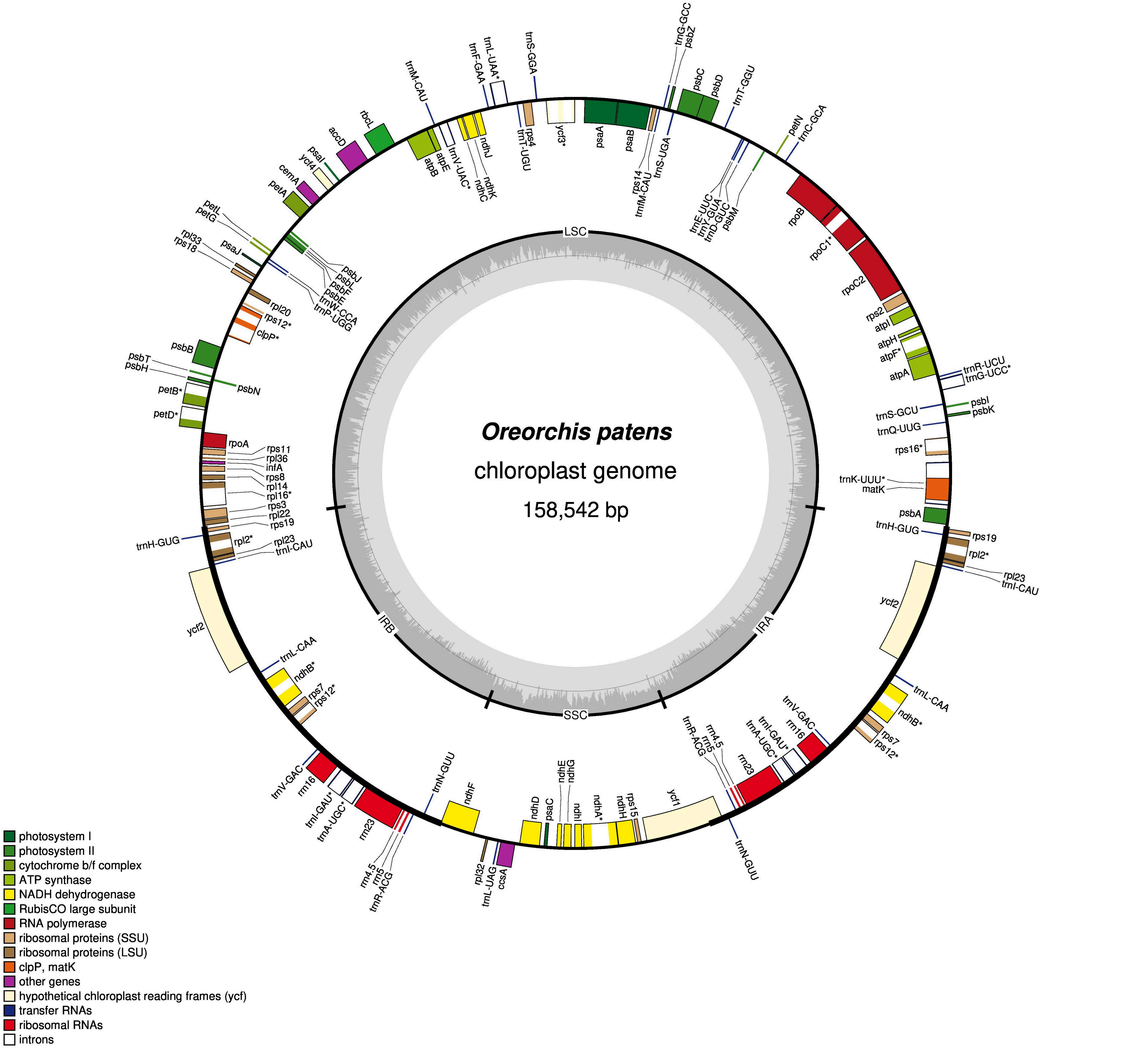

Epidendroideae
Gastrodieae

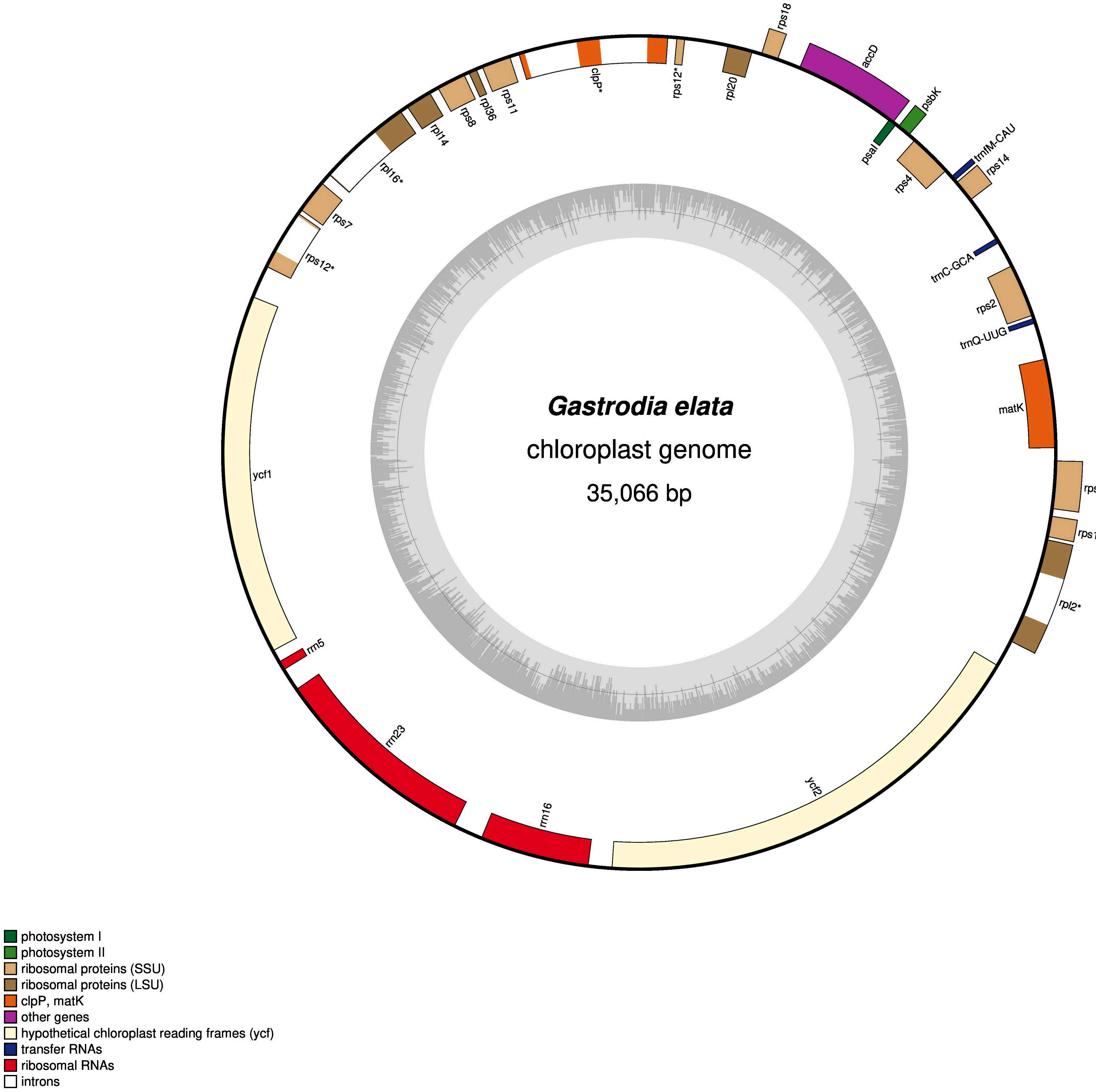

Epidendroideae
Malaxideae

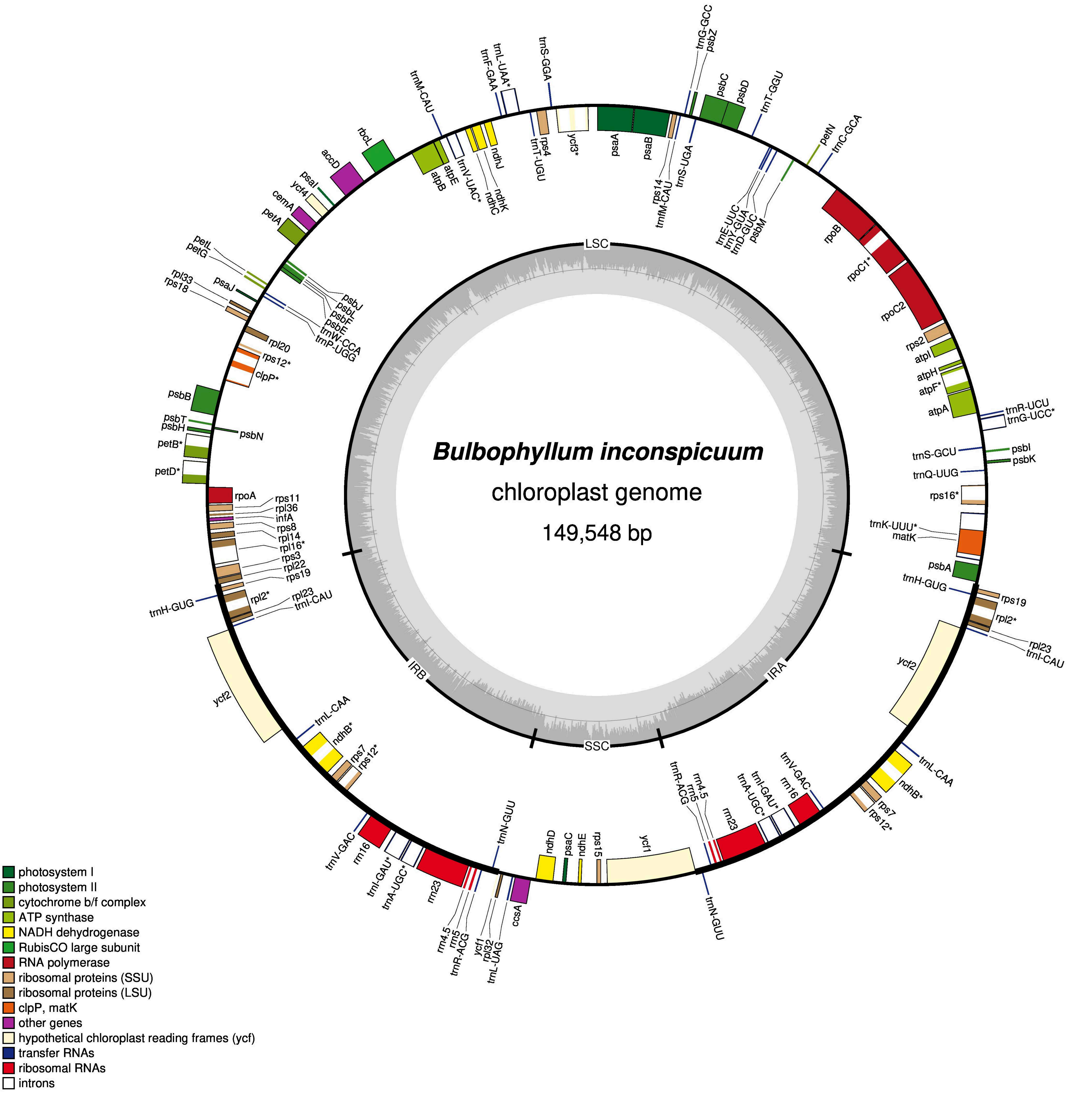

Epidendroideae
Malaxideae

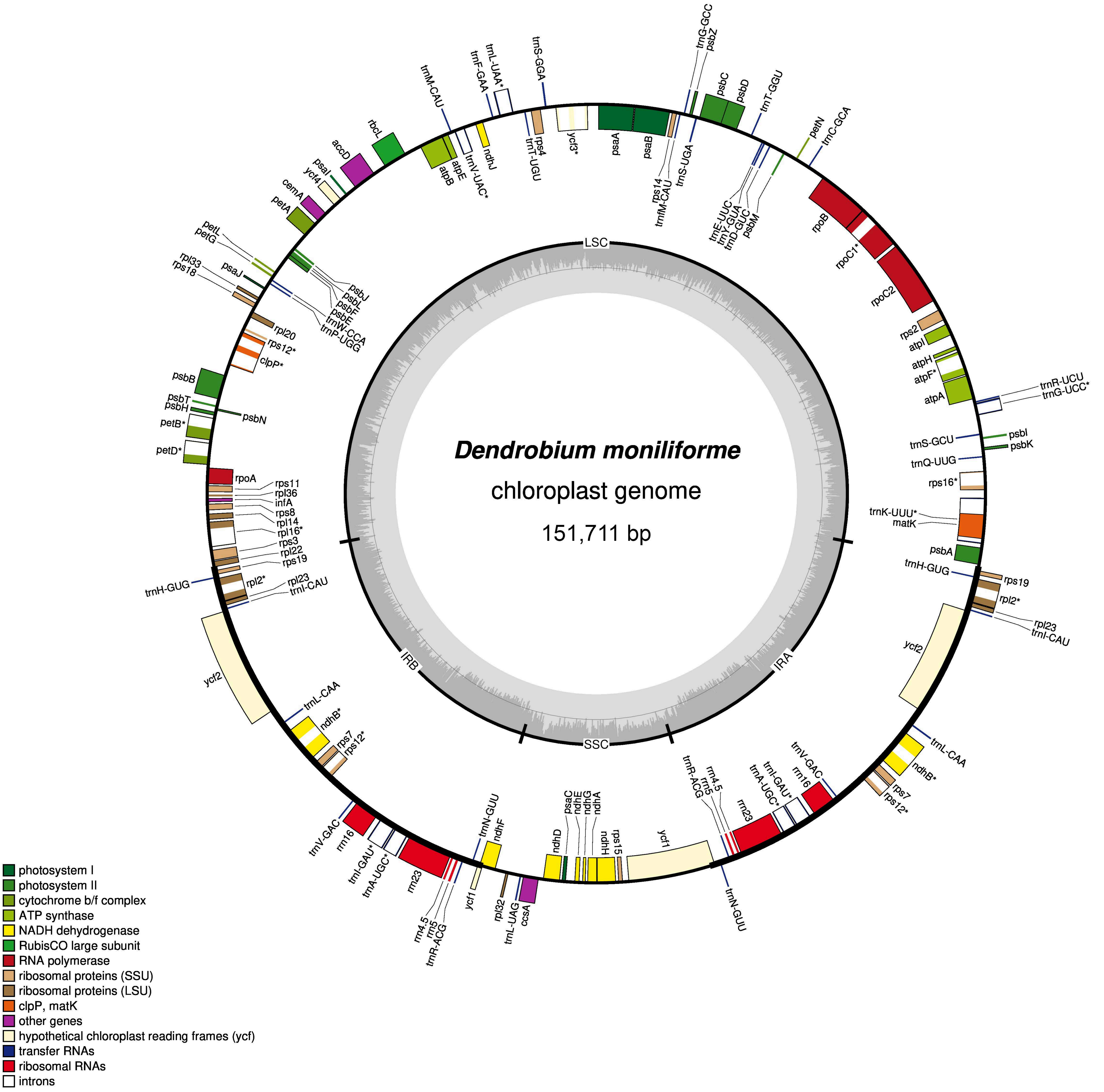

Epidendroideae
Vandeae

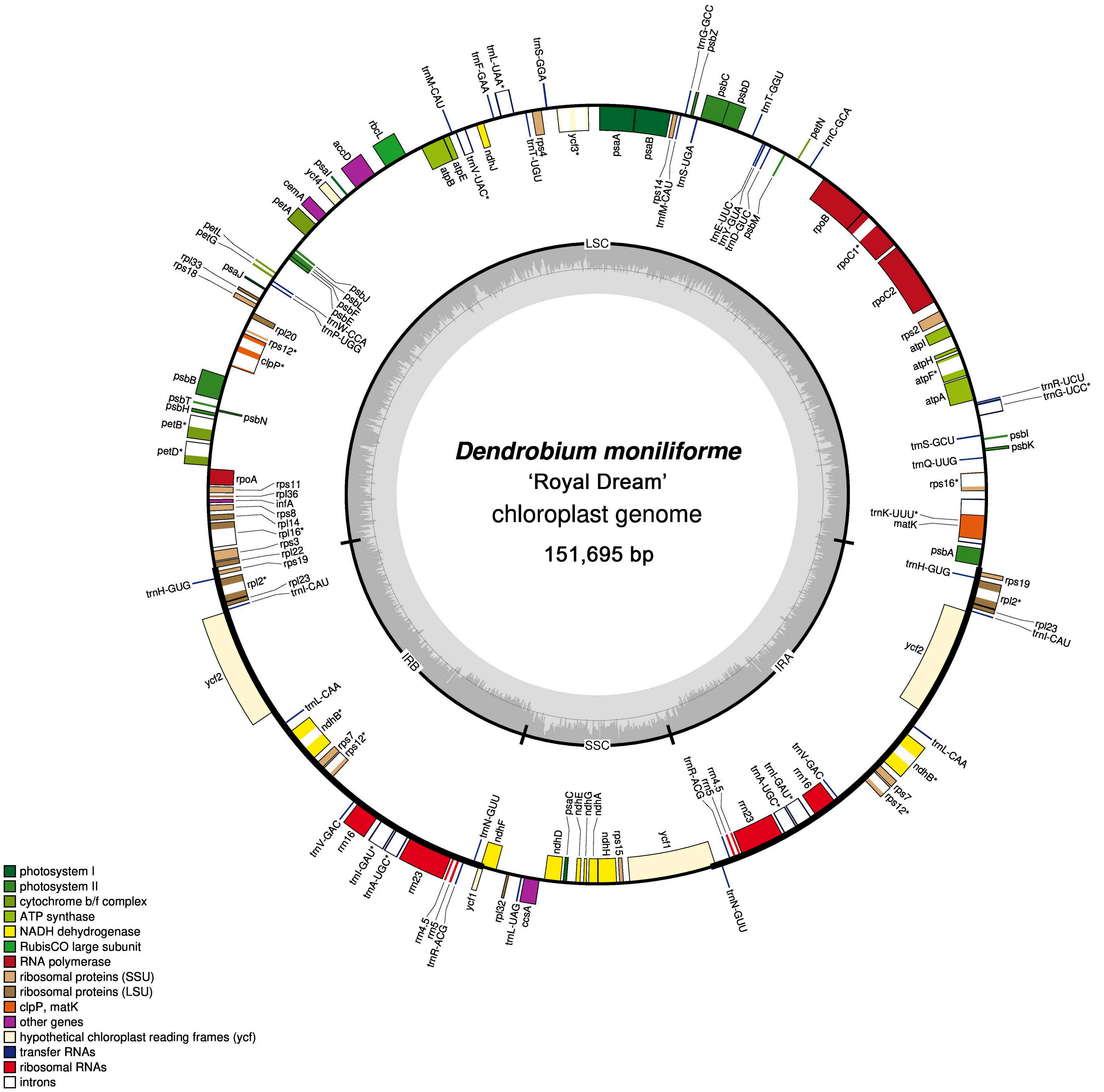

Epidendroideae
Malaxideae

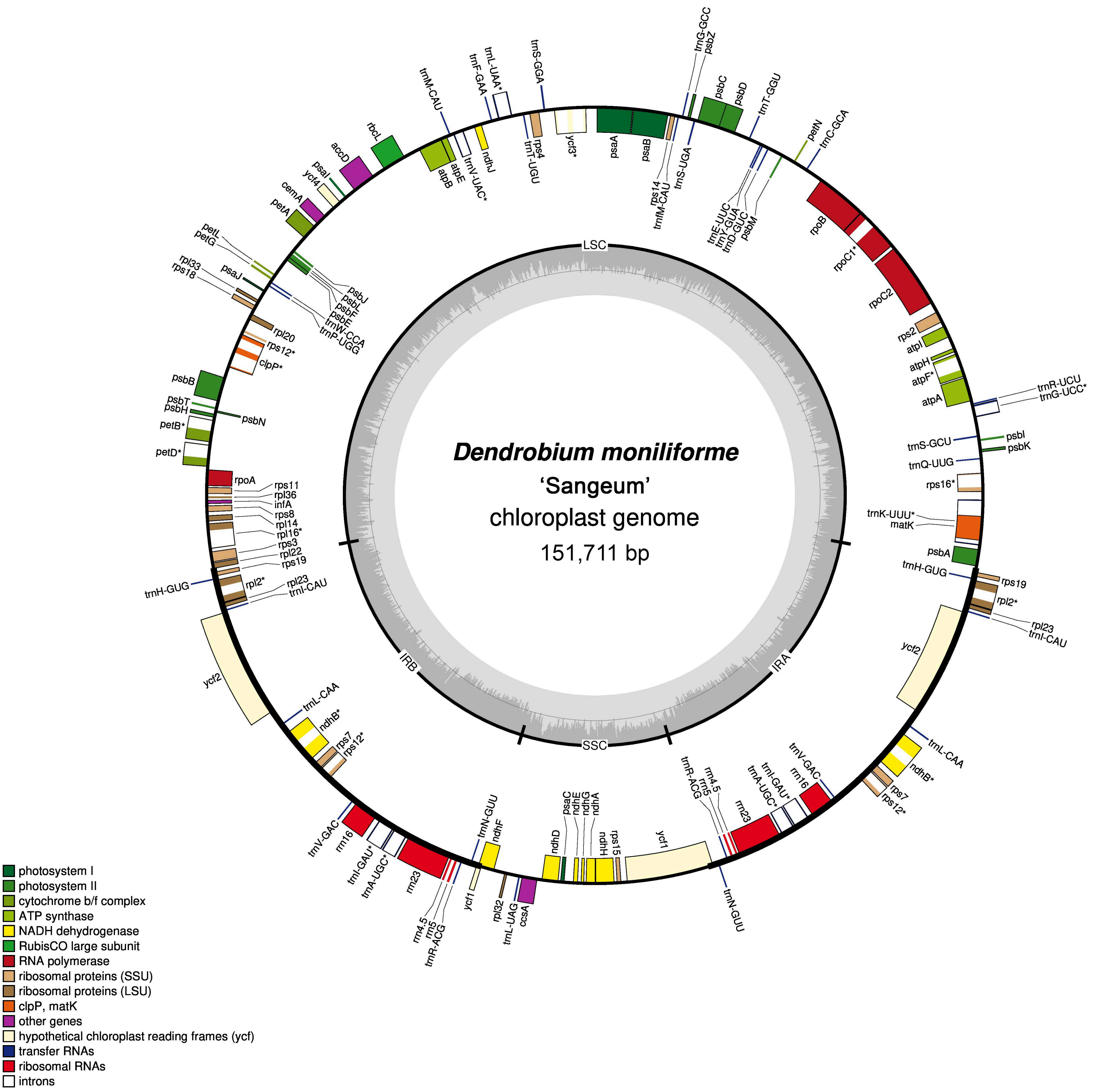

Epidendroideae
Malaxideae

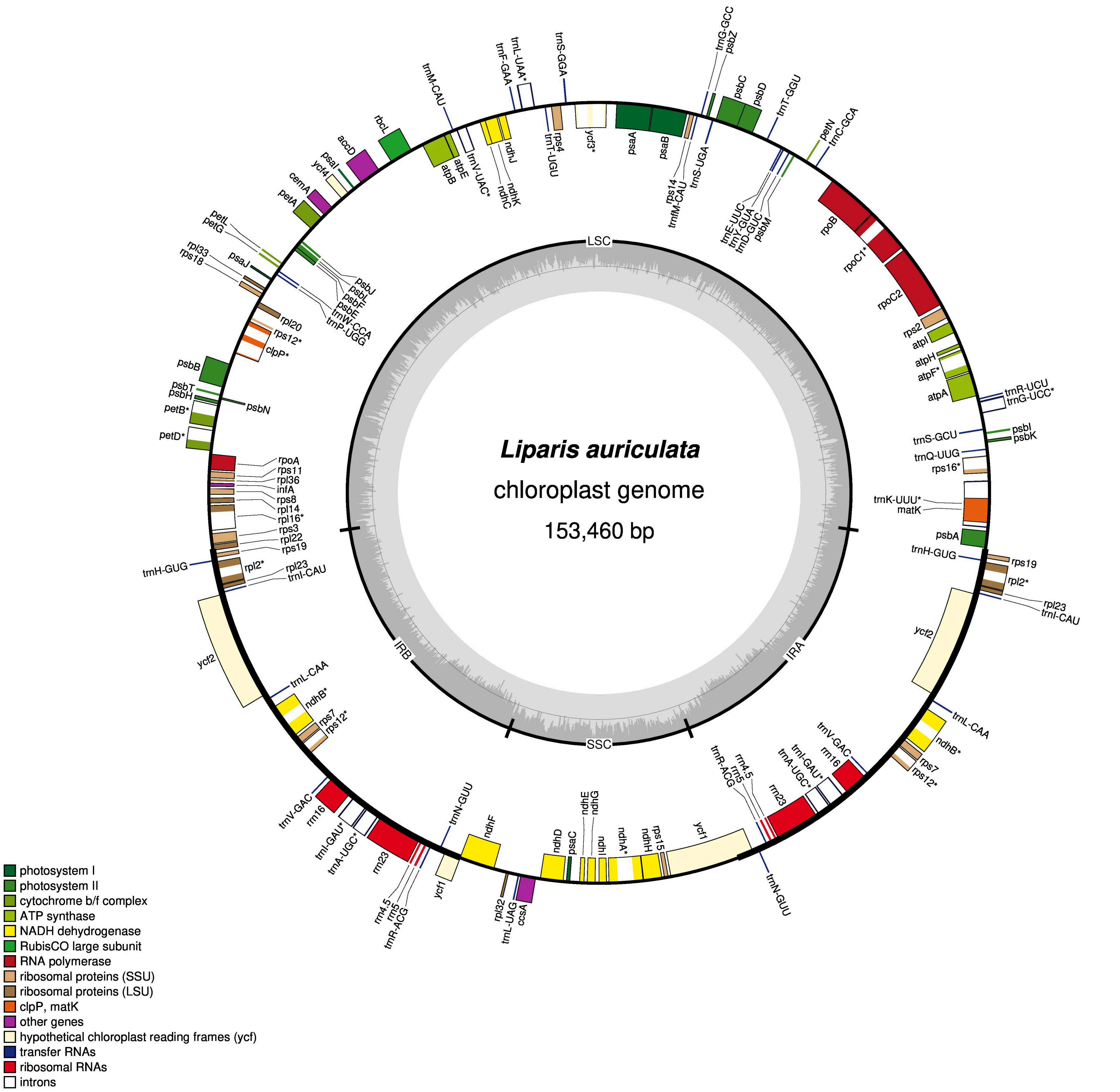

Epidendroideae
Malaxideae

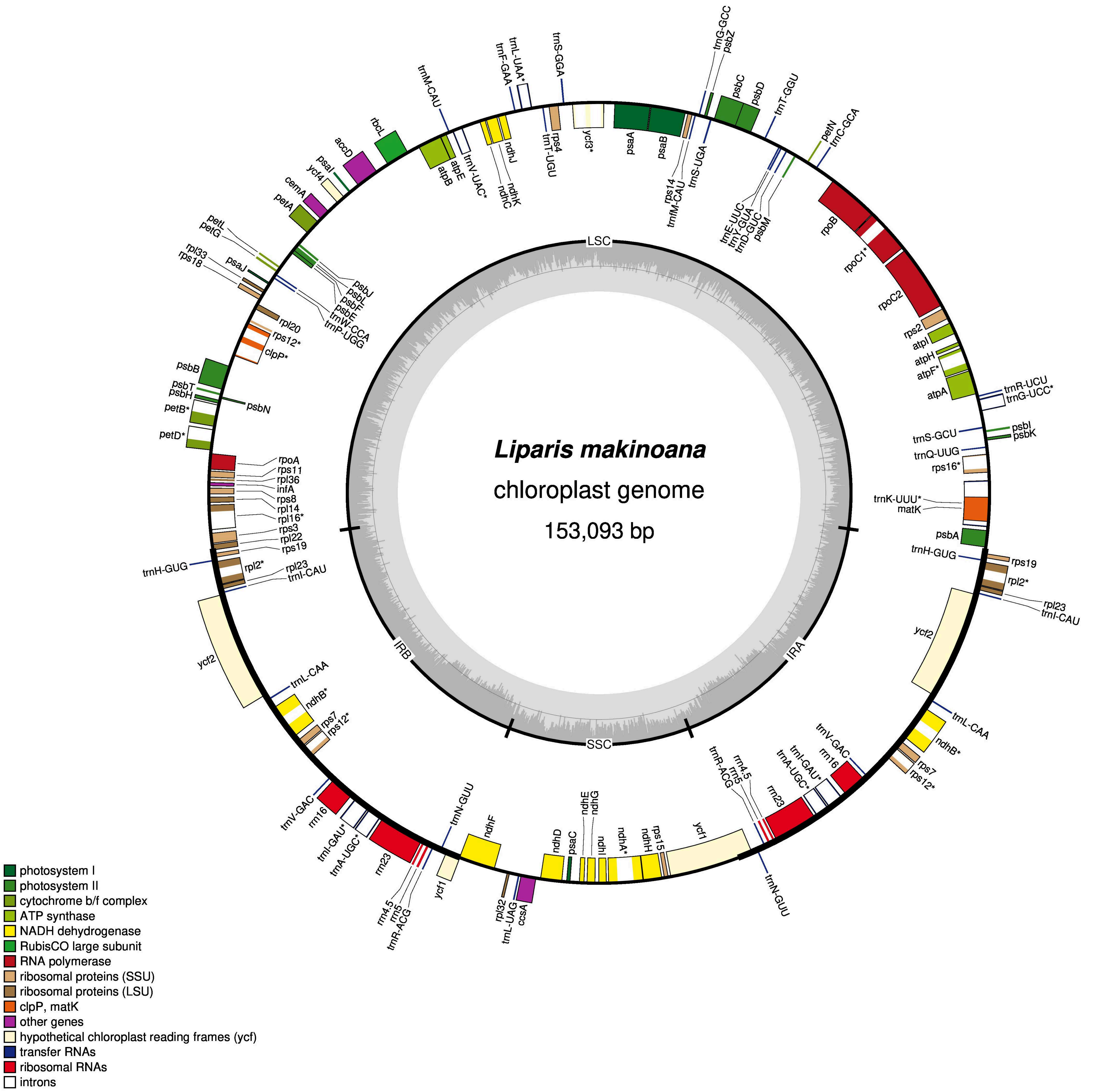

Epidendroideae
Neottieae

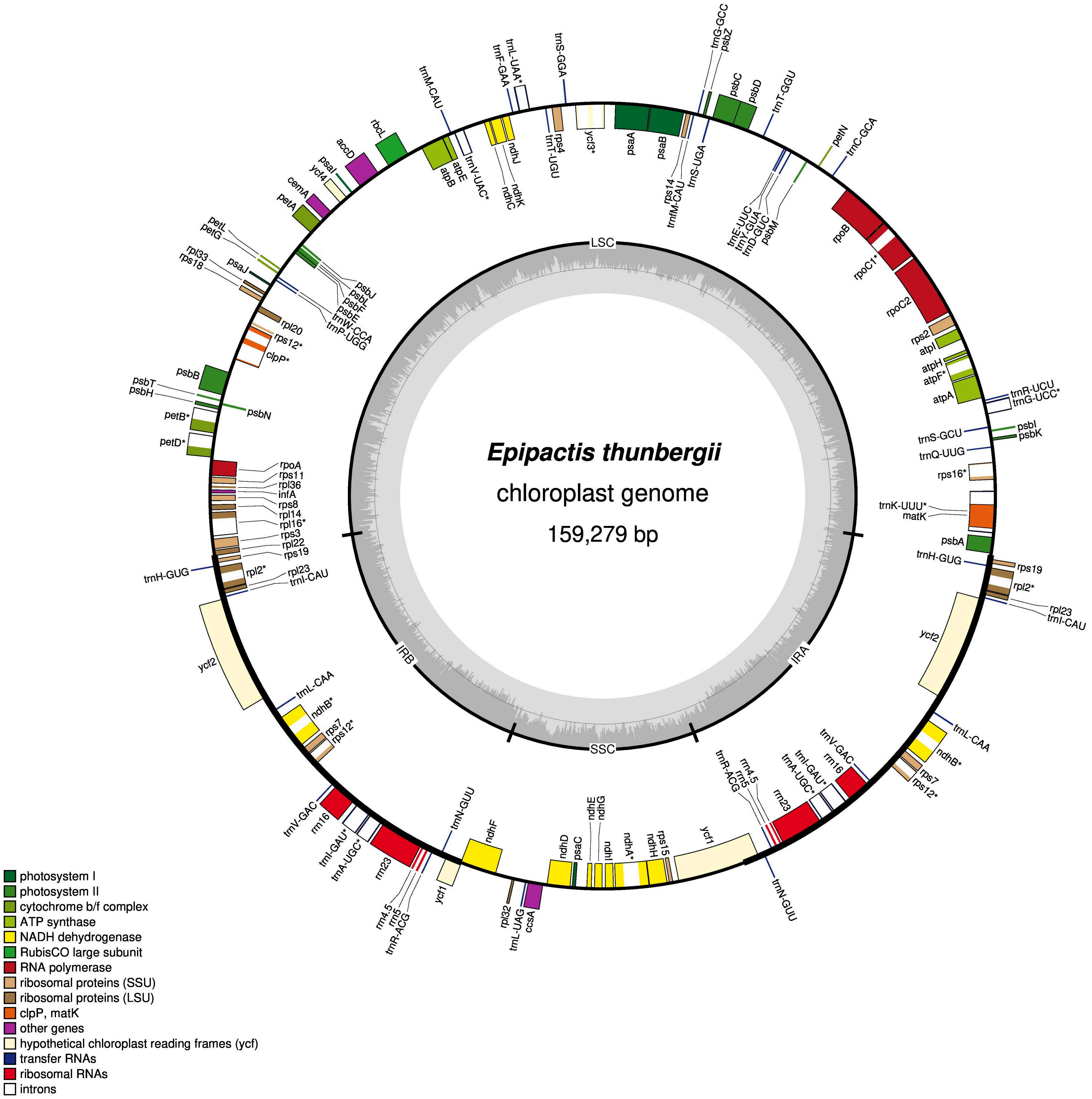

Epidendroideae
Vandeae

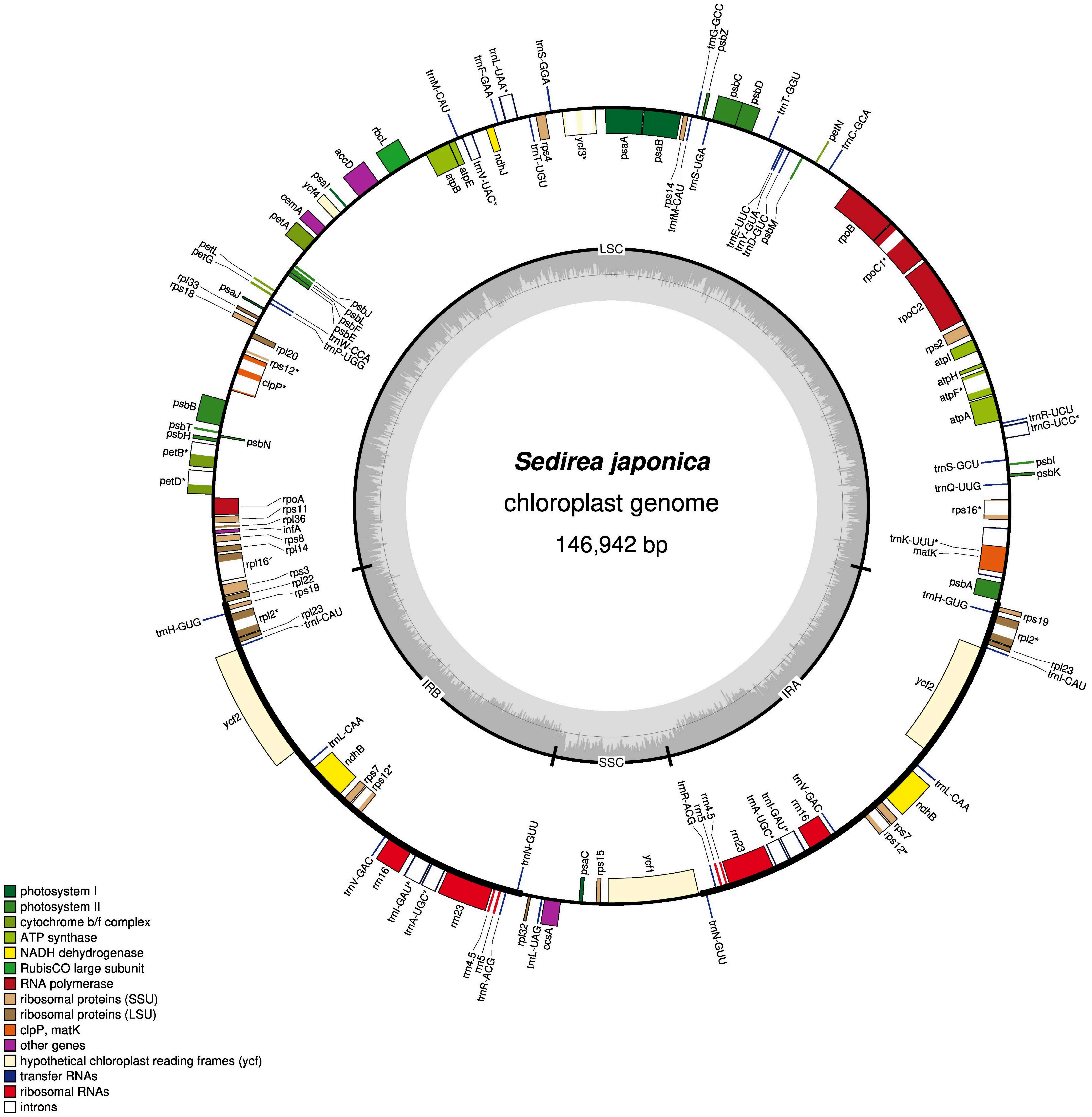

Orchidoideae
Cranichideae

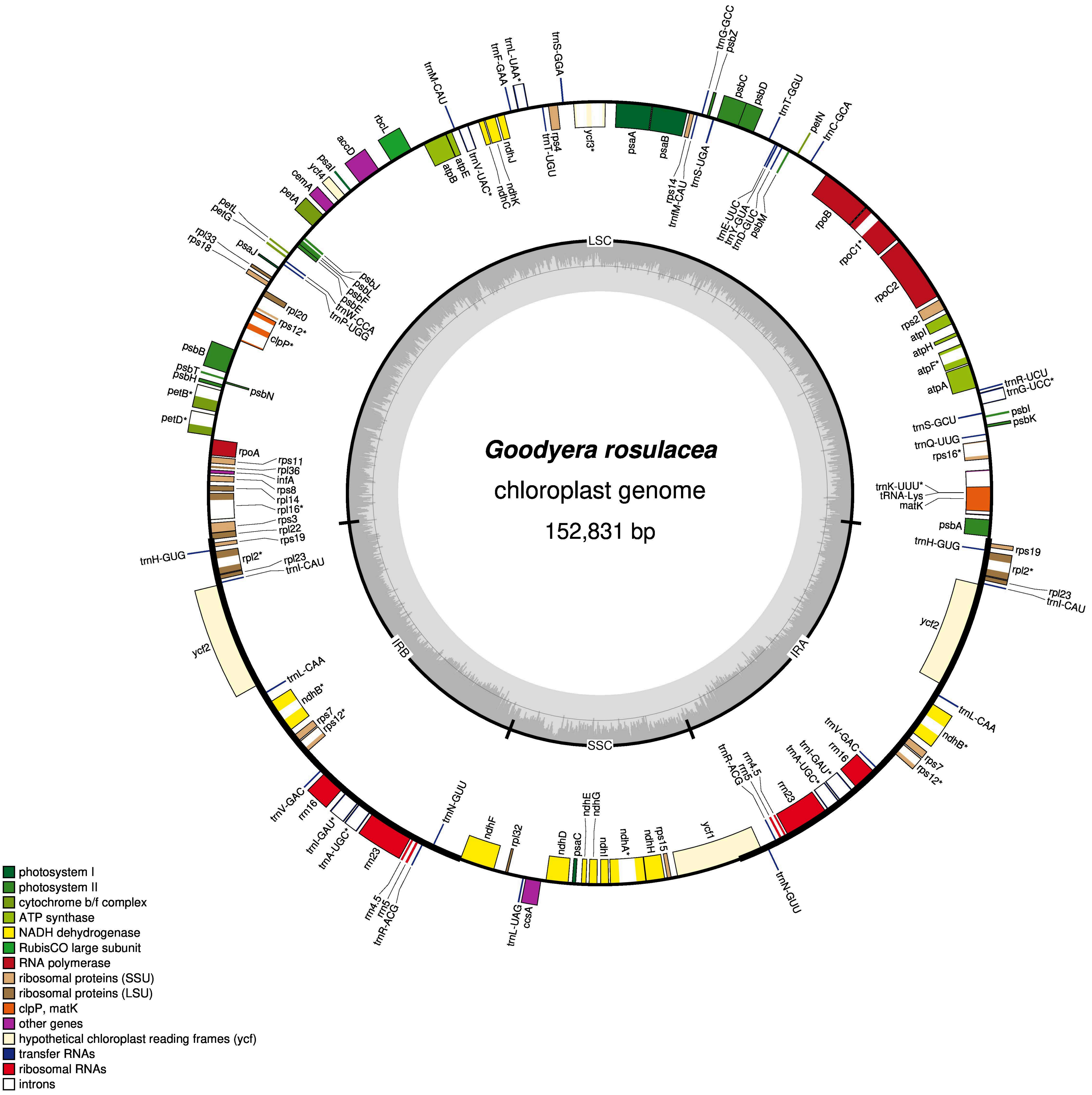

Orchidoideae
Cranichideae

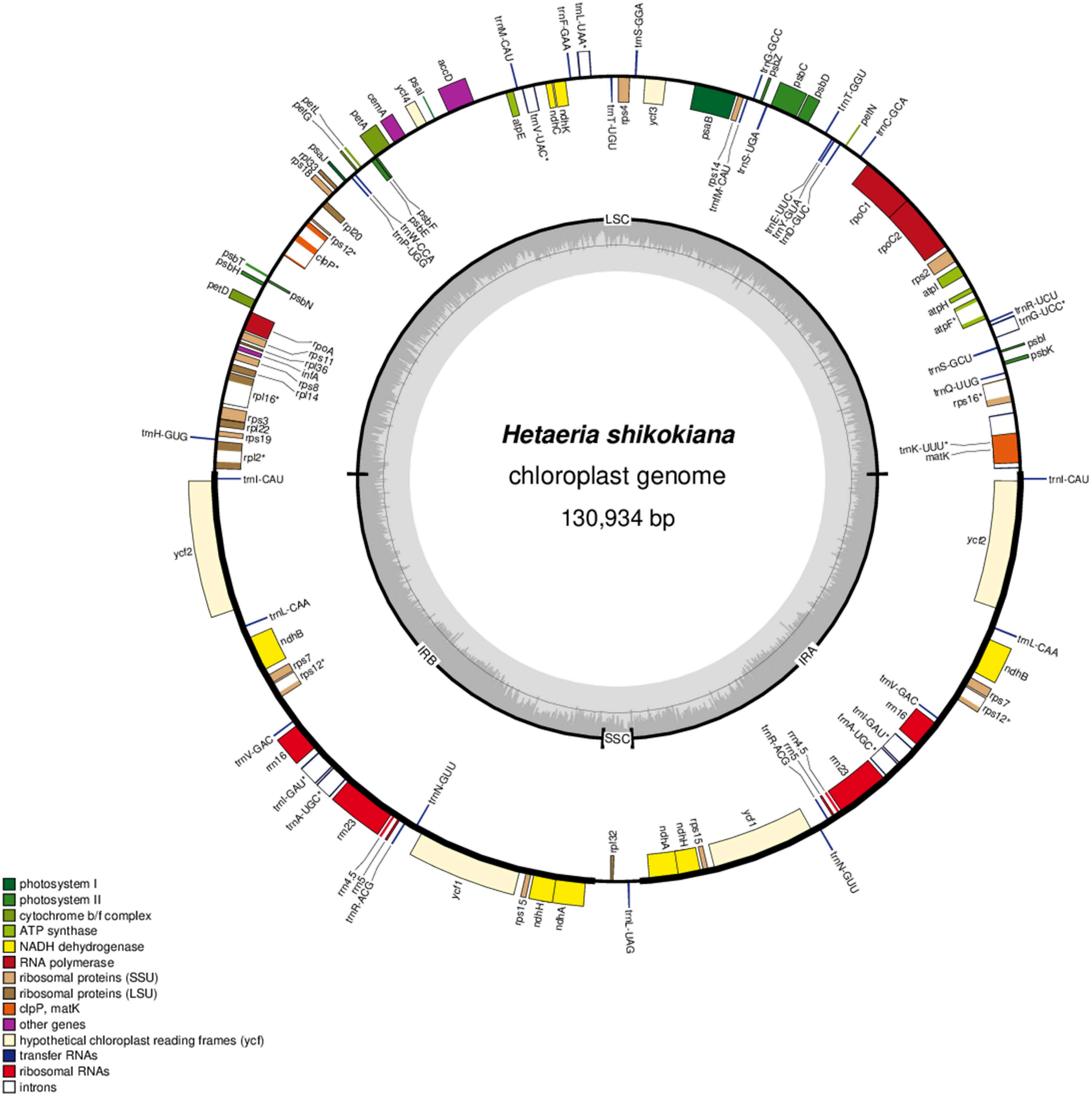

Orchidoideae
Orchideae

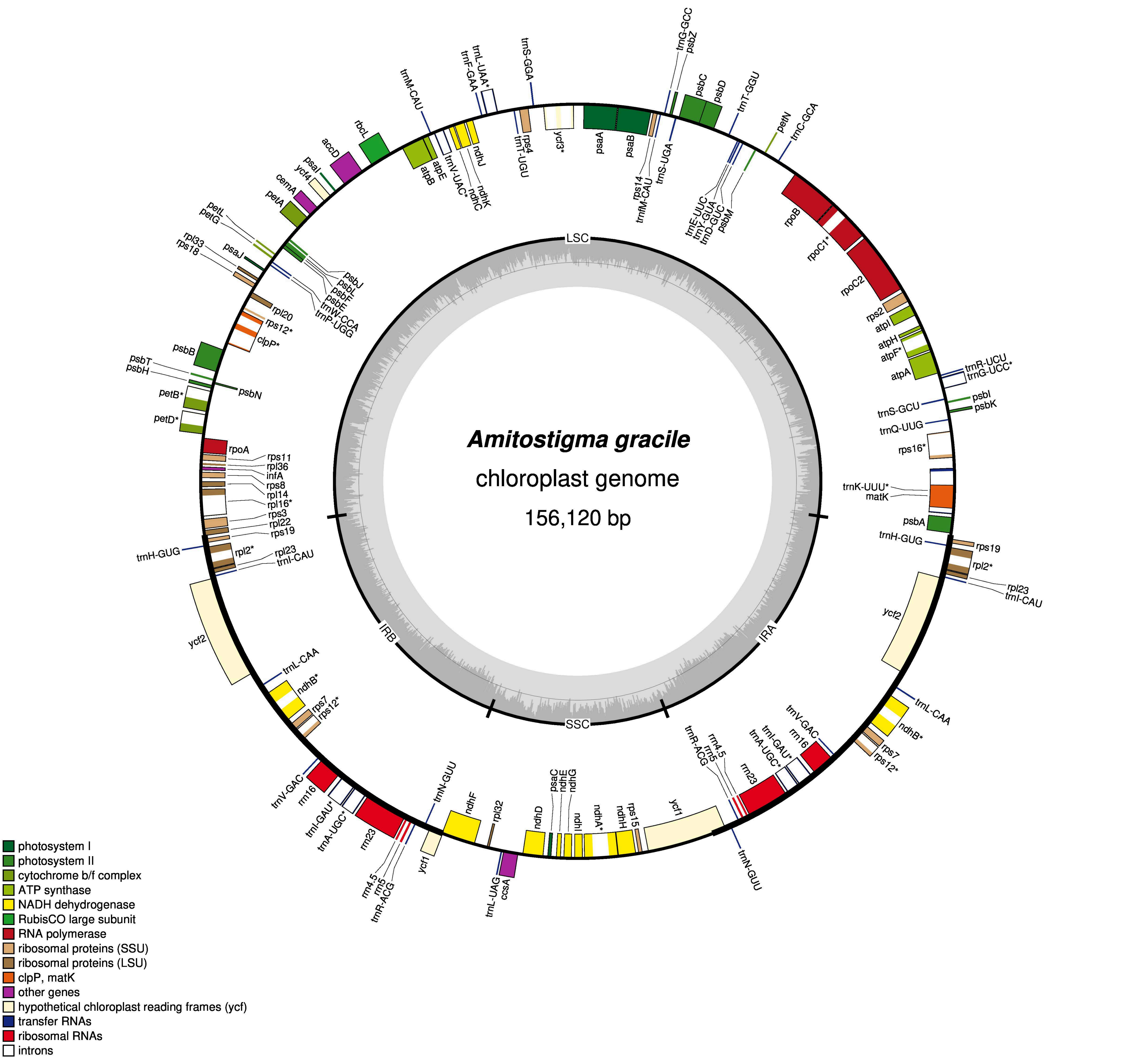

Orchidoideae
Orchideae

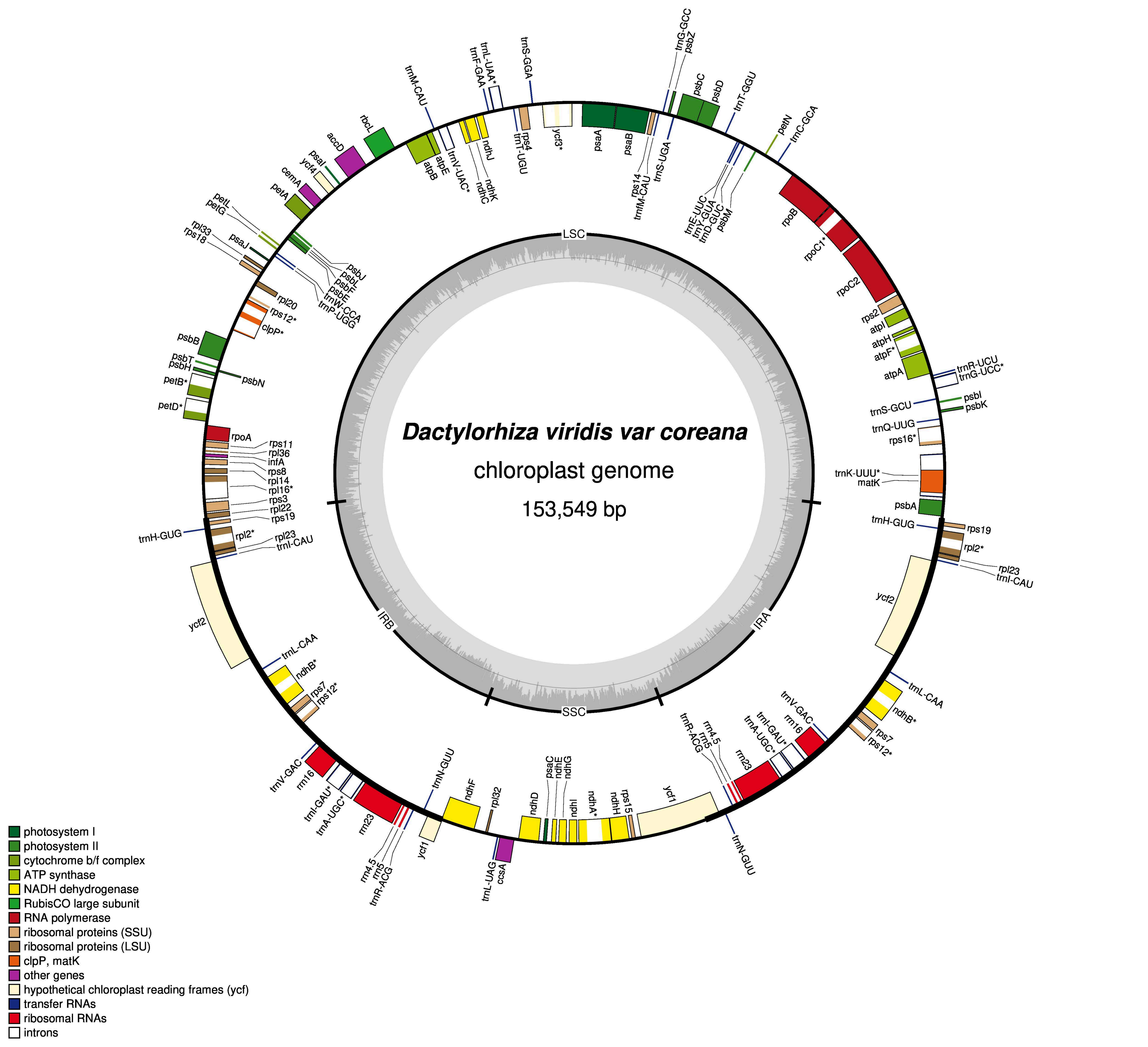

Orchidoideae
Orchideae

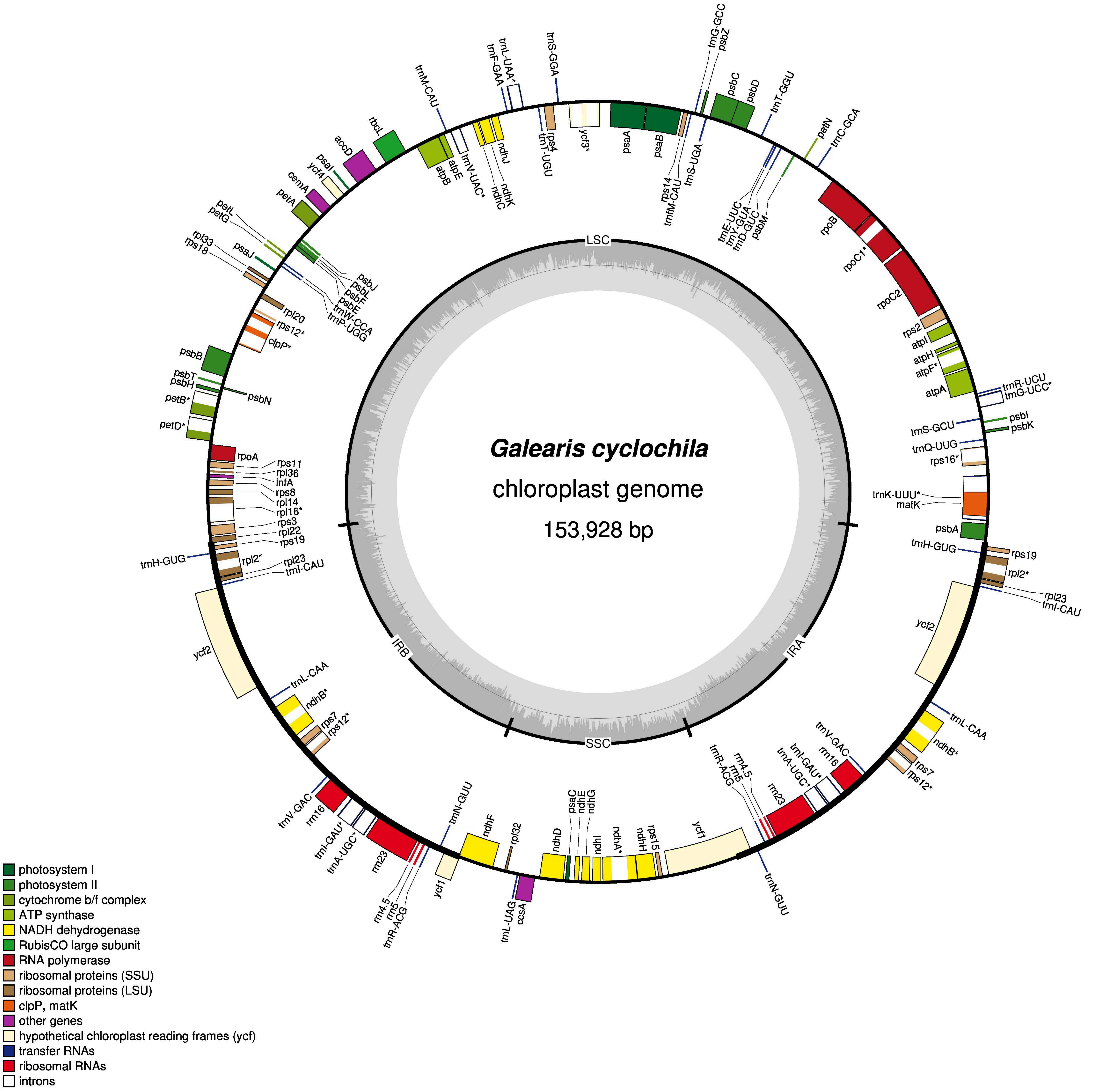

Orchidoideae
Orchideae

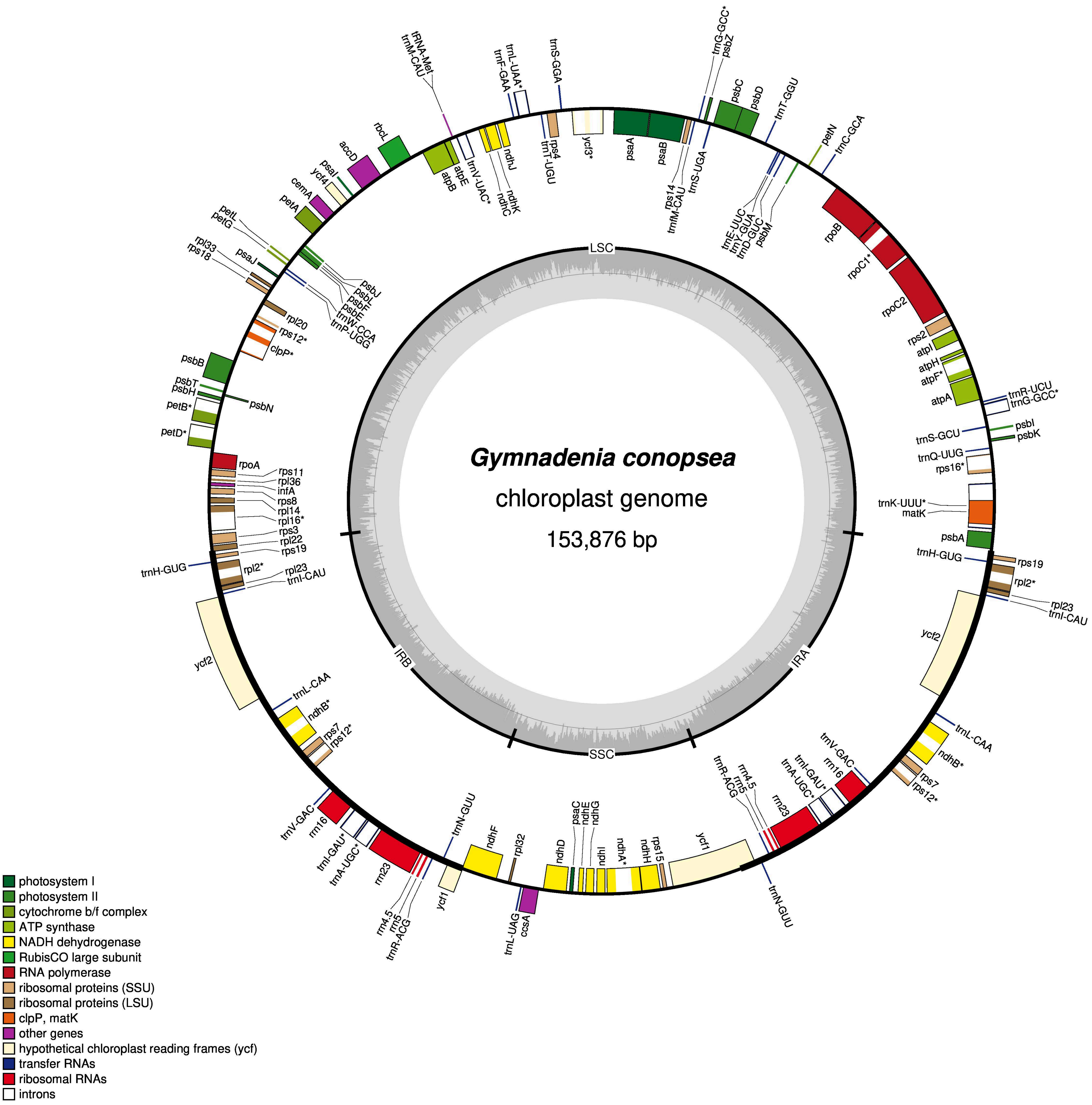

Orchidoideae
Orchideae

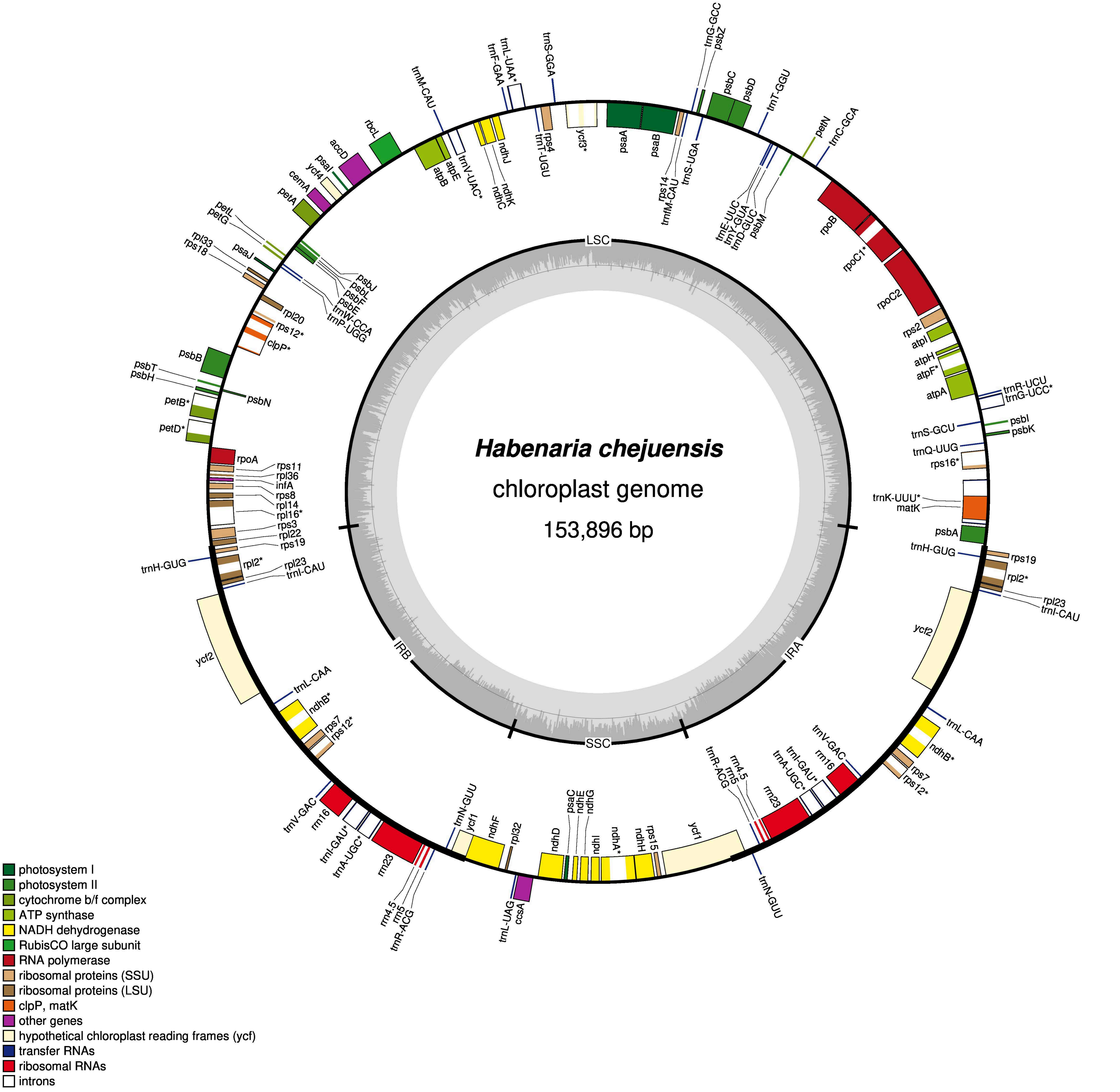

Orchidoideae
Orchideae

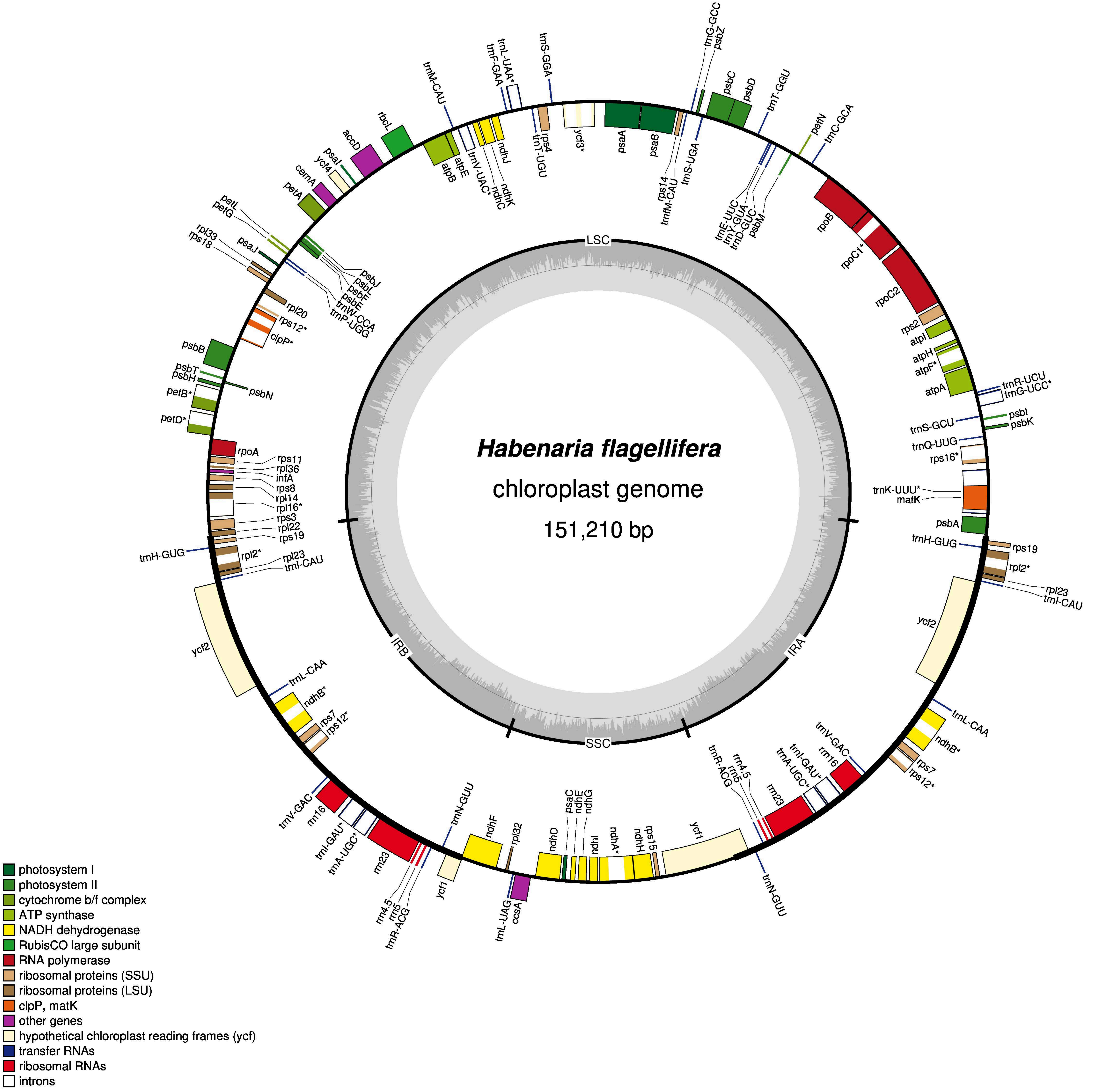

Orchidoideae
Orchideae

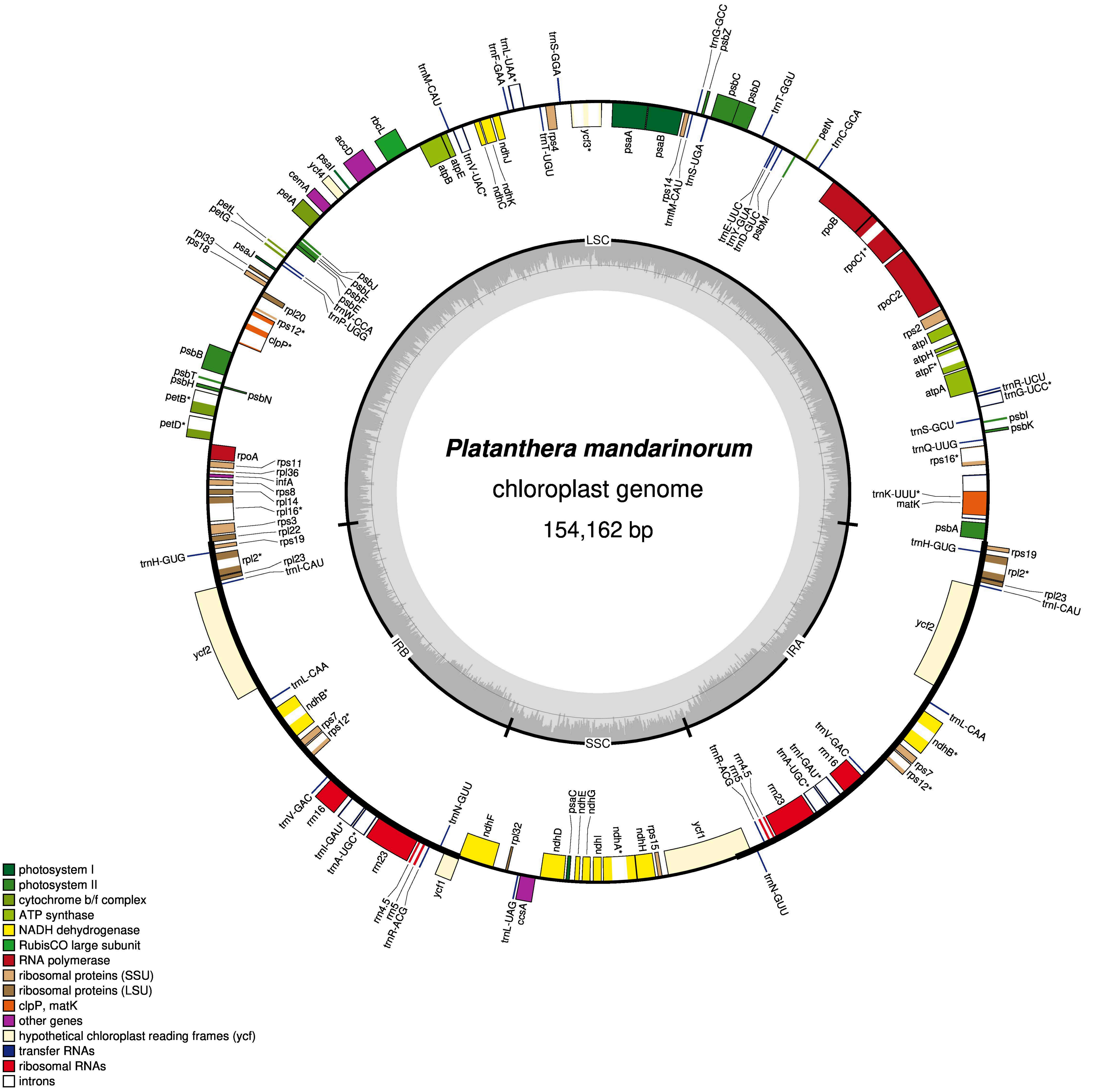

Supplement: Supplementary file 1 [file DataSheet_1.pdf]
