# Supplementary material for: Plastome Evolution and Phylogeny of Orchidaceae, With 24 New Sequences
Source: Front Plant Sci. 2020 Feb 21;11:22. doi: 10.3389/fpls.2020.00022 (PMC7047749; doi:10.3389/fpls.2020.00022)

Supplementary Figure S2. IR-SC junction comparison among forty-two plastome sequences generated in the laboratory.

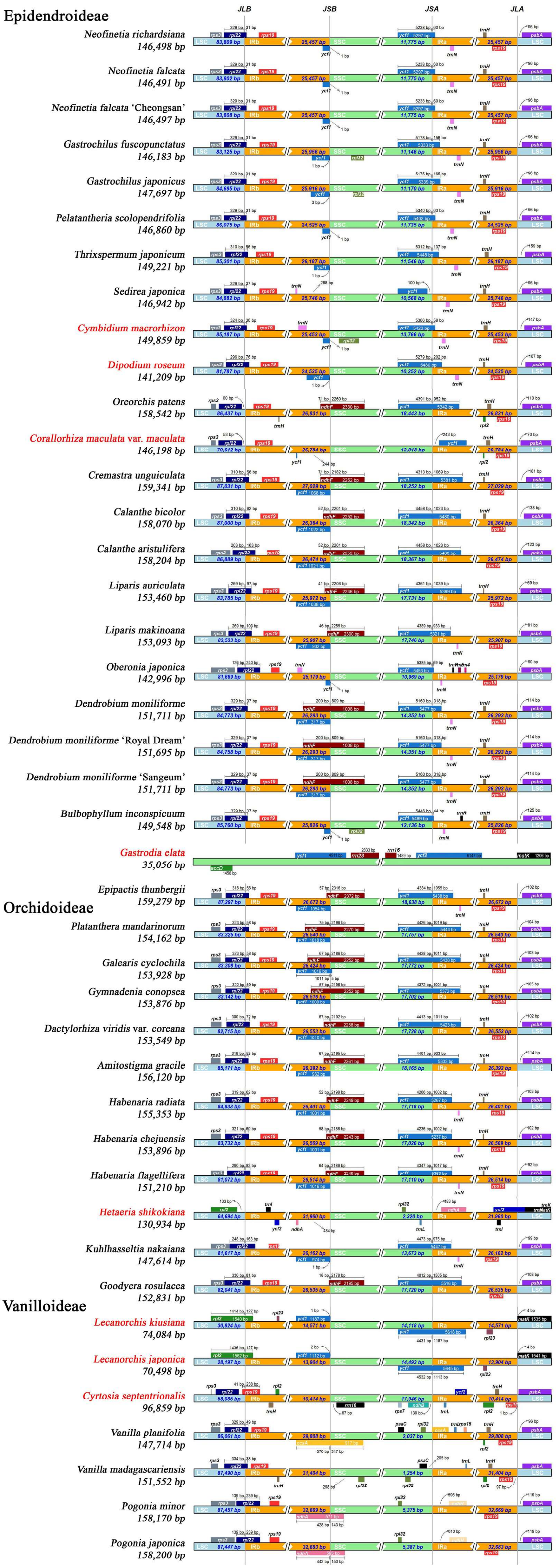

Supplement: Supplementary file 2 [file DataSheet_2.pdf]
