# Supplementary figures and images for: Plastome Evolution and Phylogeny of Orchidaceae, With 24 New Sequences
Source: Front Plant Sci. 2020 Feb 21;11:22. doi: 10.3389/fpls.2020.00022 (PMC7047749; doi:10.3389/fpls.2020.00022)

Supplementary Figure S3. A maximum likelihood tree made using 83 genes among 129 species.

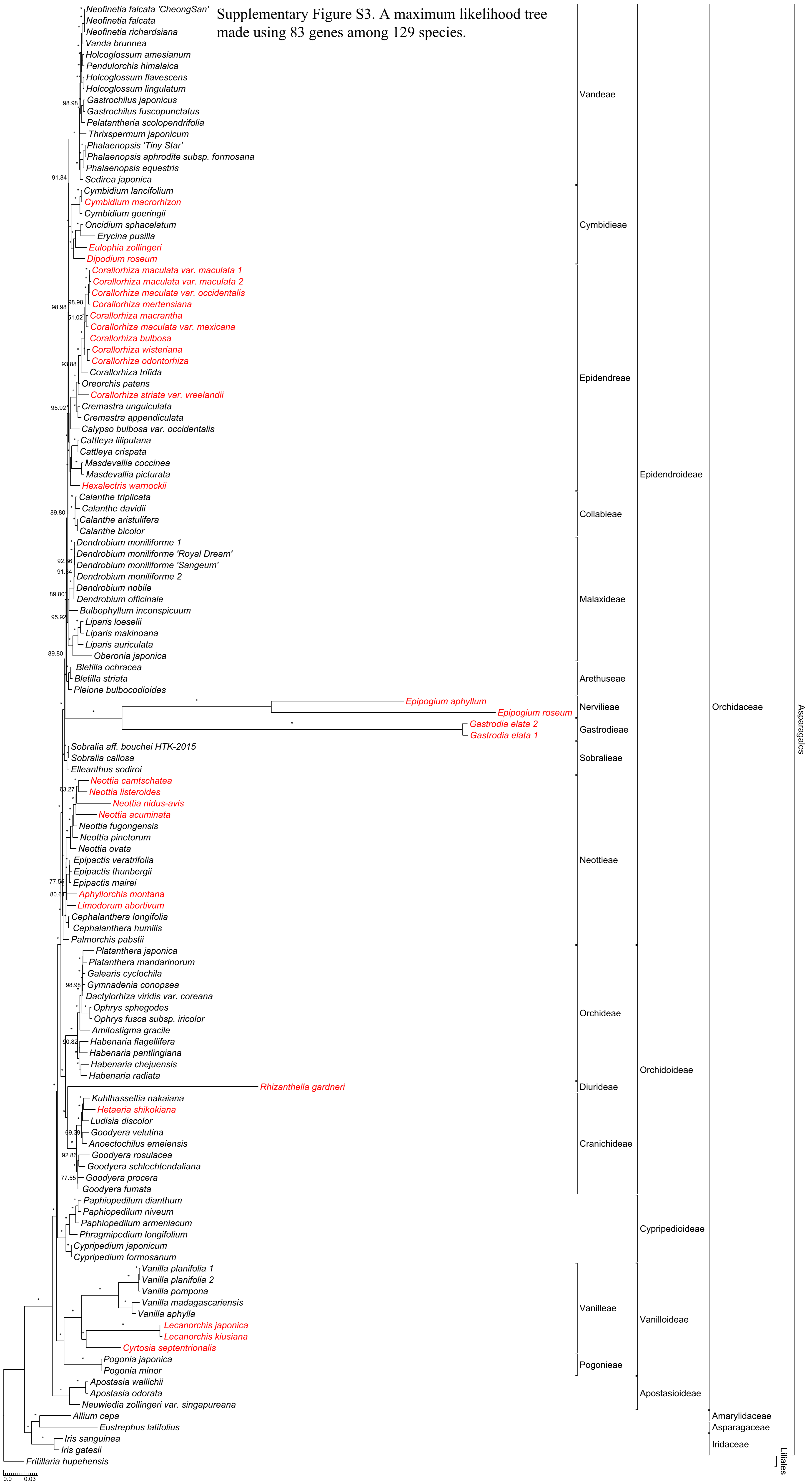

Supplement: Supplementary file 3 [file DataSheet_3.pdf]

Supplementary Figure S4. A Bayesian inference tree made using 83 genes among 129 species.

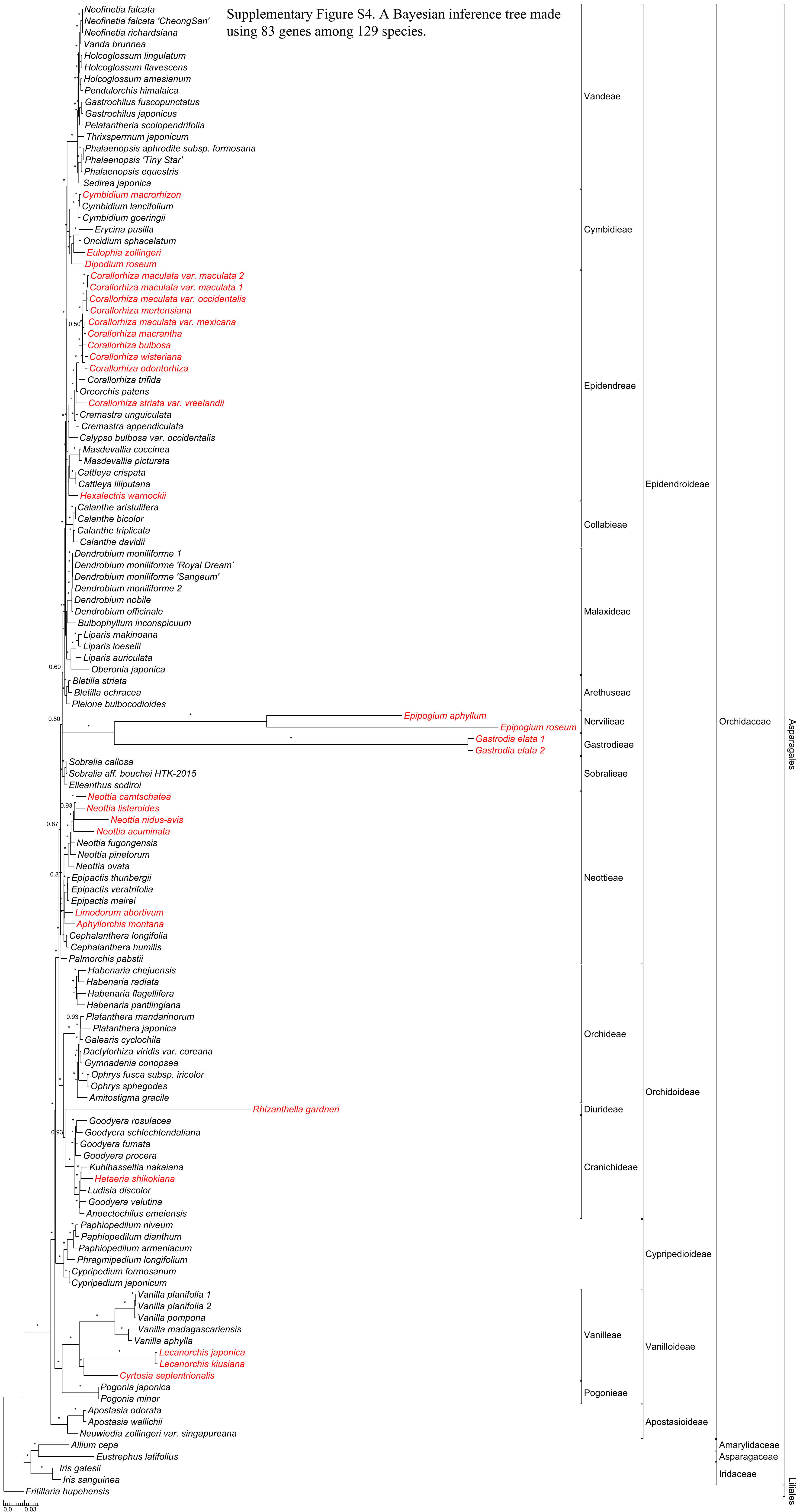

Supplement: Supplementary file 4 [file DataSheet_4.pdf]
